# Comprehensive proteogenomic characterization reveals clinically relevant molecular subtypes associated with medulloblastoma progression

**DOI:** 10.1038/s12276-026-01732-0

**Published:** 2026-06-05

**Authors:** Seong-Min Park, Kyung-Hee Kim, Jong Hyuk Yoon, Fulvio D’Angelo, Seung Ah Choi, Chan Il Kim, Harim Koo, Seungmin Park, Hyondeog Kim, Sreeja Sundara Raj, Sung Soo Kim, Ae Kyung Park, Eun Jung Koh, Seong-Ik Kim, Yu-Mi Shim, Lee Kwang-Hoon, Eric Eunshik Kim, Ji Hoon Phi, Yeon Suk Jo, Do Hyun Nam, Daehee Hwang, Do Young Hyeon, Sunghyun Huh, Hyung Joon Kwon, Seokjun Ha, Sanha Park, Hyeji Shin, Jeong Taik Kwon, Heon Yoo, Ho-Shin Gwak, Michael D. Taylor, Bong Jin Park, Jason K. Sa, Youngwook Kim, Antonio Iavarone, Sung-Hye Park, Seung-Ki Kim, Jong Bae Park

**Affiliations:** 1 Department of Cancer Biomedical Science, Graduate School of Cancer Science and Policy, National Cancer Center, Goyang, Gyeonggi Republic of Korea; 2 Proteomics Core Facility, Research Core Center, Research Institute, National Cancer Center, Goyang, Gyeonggi Republic of Korea; 3 Department of Applied Chemistry, School of Science and Technology, Kookmin University, Seoul, Republic of Korea; 4 Neurodegenerative Diseases Research Group, Korea Brain Research Institute, Daegu, Republic of Korea; 5 Sylvester Comprehensive Cancer Center, University of Miami Miller School of Medicine, Miami, FL USA; 6 Division of Pediatric Neurosurgery, Pediatric Clinical Neuroscience Center, Seoul National University Children’s Hospital, Seoul, Republic of Korea; 7 Department of Medical Science Convergence, Graduate School of Medical Science, University of Ulsan, Ulsan, Korea; 8 Department of Pharmacy, School of Pharmacy and Institute of New Drug Development, Jeonbuk National University, Jeonju, Republic of Korea; 9 Department of Neurosurgery, Seoul National University Hospital, Seoul National University College of Medicine, Seoul, Republic of Korea; 10 Department of Pathology, Seoul National University Hospital, Seoul National University College of Medicine, Seoul, Republic of Korea; 11 Department of Brain-Cognitive Science, Daegu-Gyeongbuk Institute of Science and Technology (DGIST), Daegu, Republic of Korea; 12 AIMEDBIO, Songpa-gu, Seoul, Republic of Korea; 13 School of Biological Sciences, Seoul National University, Seoul, Republic of Korea; 14 Department of Neurosurgery, Chung-Ang University Hospital, Chung-Ang University College of Medicine, Seoul, Republic of Korea; 15 Research Institute and Hospital of National Cancer Center, Goyang, Republic of Korea; 16 The Arthur and Sonia Labatt Brain Tumour Research Centre, The Hospital for Sick Children, Toronto, Ontario Canada; 17 Developmental and Stem Cell Biology Program, The Hospital for Sick Children, Toronto, Ontario Canada; 18 Department of Medical Biophysics, University of Toronto, Toronto, Ontario Canada; 19 Department of Laboratory Medicine and Pathobiology, University of Toronto, Toronto, Ontario Canada; 20 Texas Children’s Cancer and Hematology Center, Houston, TX USA; 21 Department of Pediatrics — Hematology/Oncology, Baylor College of Medicine, Houston, TX USA; 22 Department of Neurosurgery, Baylor College of Medicine, Houston, TX USA; 23 Department of Neurosurgery, Texas Children’s Hospital, Houston, TX USA; 24 Department of Surgery, University of Toronto, Toronto, Ontario Canada; 25 Dan L Duncan Comprehensive Cancer Center, Baylor College of Medicine, Houston, TX USA; 26 Department of Neurosurgery, Kyung Hee University College of Medicine, Seoul, Republic of Korea; 27 Department of Biomedical Informatics, Korea University College of Medicine, Seoul, Republic of Korea; 28 Department of Biomedical Sciences, Korea University College of Medicine, Seoul, Republic of Korea; 29 Department of Neurological Surgery, University of Miami Miller School of Medicine, Miami, FL USA; 30 Neuroscience Research Institute, Seoul National University College of Medicine, Seoul, Republic of Korea; 31 Center for Multi-omics, Medical Science Research Institute, Kyung Hee University College of Medicine, Seoul, Republic of Korea

**Keywords:** Cancer microenvironment, Inflammation, Cancer metabolism, Autophagy

## Abstract

Current treatment strategies for medulloblastoma remain ineffective owing to extensive tumor heterogeneity. We generated five platforms of omics data including liquid chromatography and mass spectrometry-based proteome and performed integrated multi-omic characterization to improve the conventional molecular classification of medulloblastoma. We identified seven refined distinct subtypes. The sonic hedgehog (SHH) group was reclassified into two subgroups, SHHα and SHHβ, whereas group 4 was divided into three subgroups, G4α, G4β, and G4γ. SHH and group 4 subtypes exhibit two distinct neuronal differentiation trajectories: granular neuron and unipolar brush cell differentiation (SHHβ and G4γ, respectively), both of which associated with more favorable clinical outcomes. Furthermore, we uncovered unique proteomic and kinomic properties that conferred increased treatment vulnerabilities to targeted therapeutic interventions against each of the three medulloblastoma subtypes associated with poor clinical outcomes. We demonstrated the therapeutic potential of exploiting these vulnerabilities by utilizing a proteasome inhibitor and subtype-specific agents, including CDK1/2, PARP, CLK1, and MET inhibitors. Mechanistic insights were further elucidated through in-depth proteome analyses. Our study qualifies the use of proteomic signatures and activation of neuronal differentiation trajectories to tailor selective therapeutic opportunities for distinct subgroups of patients with medulloblastoma.

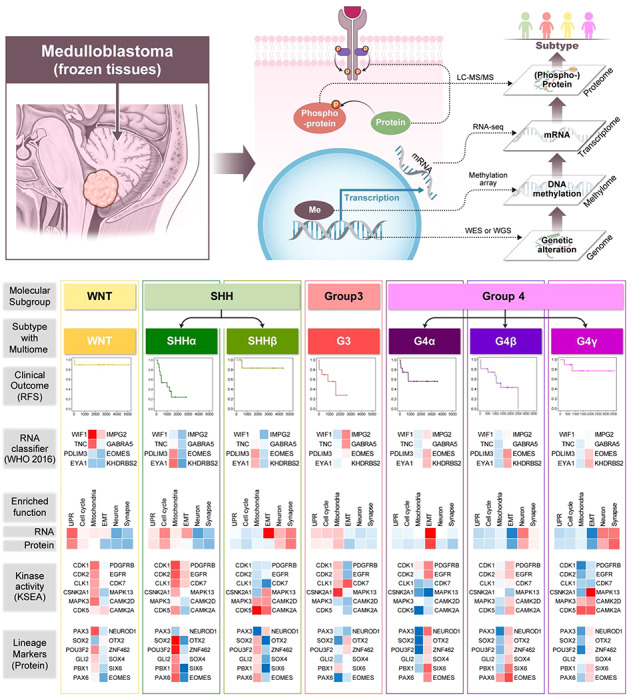

## New subtypes of medulloblastoma reveal targeted treatment paths

Medulloblastoma, a severe pediatric brain cancer, still relies on chemotherapy and radiotherapy post-surgery, leading to significant side effects and poor long-term survival rates. This study addresses the need for personalized treatments by conducting a comprehensive multi-omics analysis on medulloblastoma specimens, including genomics, transcriptomics, methylomics, proteomics, and phosphoproteomics. Researchers identified seven distinct subtypes, refining the traditional four-subgroup classification and revealing subtype-specific progression patterns and therapeutic targets. Notably, the sonic hedgehog (SHH) and G4 groups were further subdivided into SHHα and SHHβ and G4α, G4β, and G4γ, with SHHβ and G4γ demonstrating improved prognosis associated with enhanced neuronal differentiation. The study underscores the importance of proteogenomic insights, indicating that targeting subtype-specific pathways may lead to more effective therapeutic strategies. Future directions include the development and clinical evaluation of targeted therapies based on these findings to advance precision medicine for patients with medulloblastoma. This summary was initially drafted using artificial intelligence, then revised and fact-checked by the author.

## Introduction

Medulloblastoma is a representative malignant pediatric brain cancer for which the standard of care after surgery still involves conventional chemotherapy and radiotherapy, with significant toxicity and long-term morbidities^1^. Furthermore, patients with medulloblastoma frequently experience tumor recurrence toward mostly incurable stages of the disease; according to previous studies, the overall 3-year survival after relapse is 18% and the 5-year survival is only 6% (refs. ^2,3^). Thus, there is an unmet requirement for more personalized therapeutic approaches in patients with medulloblastoma.

Various omics studies have been performed with the aim of improving the molecular diagnosis of medulloblastoma. Four distinct molecular subgroups of medulloblastoma have been defined based on genomic, transcriptomic, and methylomic features (Wingless and Int-1 (WNT), sonic hedgehog (SHH), group 3, and group 4), which are now accepted as the international consensus classification of medulloblastoma^4^. The WNT and SHH subgroups are characterized by genomic mutations that activate the genetic drivers of respective pathways, including CTNNB1, PTCH1, and TP53 mutations, whereas groups 3 and 4 lack notable mutations and are instead defined by unique transcriptomic signatures^5^. By applying methylome analysis, the four subgroups were further subdivided into multiple subtypes^6^. Despite the wealth of molecular data, translation into clinical benefit remains limited. Immune profiling has indicated that, unlike many other pediatric brain tumors, medulloblastomas are generally immune cold, although subgroup-specific differences in immune infiltration and microenvironmental composition have been described^7^. Beyond DNA and RNA profiling, proteogenomic analyses have recently expanded this classification. A landmark international consortium study integrated quantitative proteomics, phosphoproteomics, and genomics from more than 170 medulloblastomas, identifying six proteome subtypes — pWNT, pSHHs, pSHHt, pG3, pG3myc, and pG4 — each with specific biological processes and prognostic implications^8^. These findings highlight the added value of proteome-level resolution for refining medulloblastoma classification and for linking subgroup identity to actionable pathways.

Although the molecular hallmarks of medulloblastoma at diagnosis opened new possibility for the use of integrated multi-omics data toward precision medicine applications, these studies failed to exhibit favorable impact for medulloblastoma treatment. In this study, we explored the molecular profiles of medulloblastoma specimens by conducting an integrated multi-omics analysis including genomics, transcriptomics, methylomics, global proteomics, and phosphoproteomics — on a large cohort of medulloblastoma specimens with longitudinal sampling. By performing integrated proteogenomic analyses, we identified new subtypes, investigated the difference in progression patterns among the subtypes, and identified progression-related biomarkers and subtype-specific therapeutic targets. Our findings provide the refined molecular background necessary to accelerate the development of precision therapy for patients with medulloblastoma.

## Materials and methods

The detailed methods can be found in the additional Supplementary material.

### Proteome analysis

Tandem mass tag (TMT)-labeled peptides prepared for global proteome and phosphoproteome analysis were resuspended with 0.1% formic acid in water, separated using an Ultimate 3000 RSLCnano system (Thermo Fisher Scientific, San Jose, CA, USA), and analyzed using a Q Exactive HF-X hybrid quadrupole-Orbitrap mass spectrometer or a Q-Exactive plus hybrid quadrupole Orbitrap mass spectrometer (Thermo Fisher Scientific).

Raw files of tandem mass spectra were converted into mzML files using the msConvert program (version 3.0, https://bio.tools/msconvert). The mzML spectral data were mapped to the human UniProt database (UP000005640.fas, https://www.uniprot.org/) and quantified using the FragPipe pipeline (version 14.0), including MSFragger, Philosopher, and TMT-integrator program (version 3.1.1, https://msfragger.nesvilab.org). The expression ratios of filtered proteins were globally normalized with the quantile normalization method using the R (version 4.0) limma package or MATLAB Bioinformatics Toolbox (version R2021a).

## Results

### Multi-omics analysis stratifies patients with medulloblastoma into seven subtypes

We conducted multi-omics profiling of 123 medulloblastomas, including primary and recurrent tumors, from 102 patients (Supplementary Fig. 1). Our analysis involved five omics platforms: genomics, transcriptomics, global proteomics, phosphoproteomics, and methylomics (Supplementary Fig. 1). Using liquid chromatography and mass spectrometry (LC-MS/MS) and TMT labeling, we generated global proteomic and phosphoproteomic data from 140 samples across 116 tumor tissues. The global proteomic analysis identified and quantified 12,963 proteins in at least one tumor sample and 8,409 unique proteins were detected across all samples. The phosphoproteomic analysis identified 47,233 unique phosphosites, in which 8,094 proteins were detected in at least one sample and 3,763 phosphosites and 3,592 proteins across all samples. After filtering proteins or phosphosites that contained more than 30% missing values, the final output resulted in 10,124 proteins, 9,992 phosphosites and 3,715 phosphoproteins for global proteome and phosphoproteome, respectively. Additionally, we generated genomic, methylomic, and transcriptomic data for 109, 106, and 112 tumor tissues, respectively, alongside genomic data from 80 matched blood. Two patients were excluded owing to histological reclassification as astroblastoma. The comprehensive summary of the sample and data status is presented in Supplementary Fig. 1.

Previous studies defined four molecular subgroups of medulloblastoma, WNT, SHH, group 3, and group 4, based on genomic, transcriptomic, and methylomic data^4,5^. We assessed whether our multi-omics data reproduced the conventional subgroups with an extended data set. Unsupervised clustering methods, including hierarchical and non-negative matrix factorization from transcriptomic and methylomic data from primary tumors, consistently identified the reported four subgroups (Supplementary Figs. 2 and 3). Consensus clustering across *k*-values (2–8) and multiple filtering thresholds (MAD 10–30%) yielded stable solutions, with cophenetic correlations supporting four major clusters. For methylation, we applied stringent probe filtering (excluding SNP-containing CpGs and low-variance probes) before consensus clustering. These analyses confirmed that our data set faithfully represents the known subgroup structure of medulloblastoma.

**Fig. 1:**
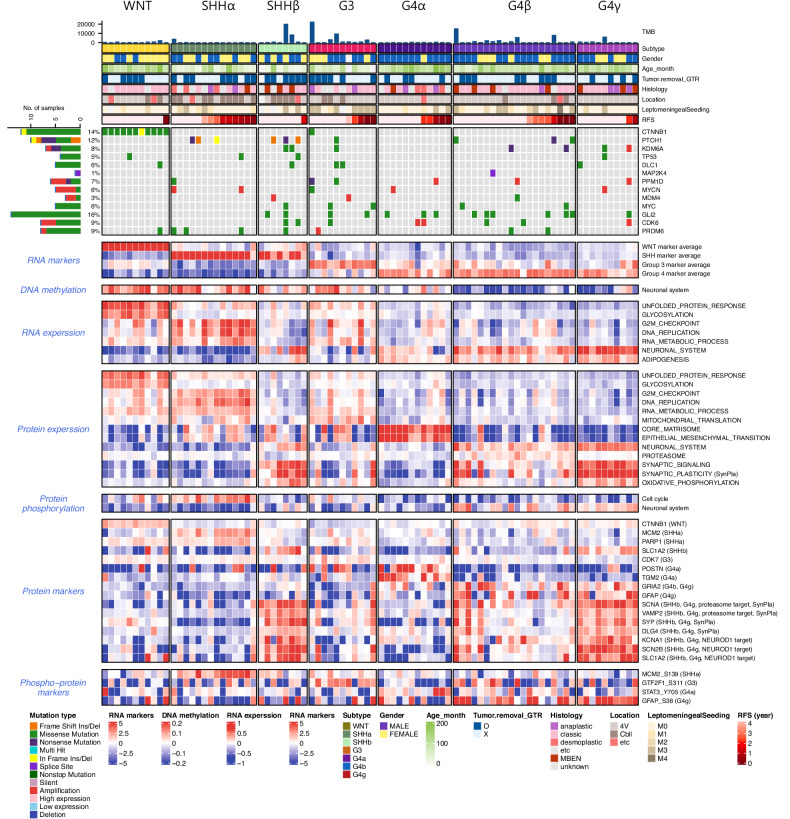
Comprehensive multi-omic profiling and markers of primary medulloblastoma subtypes. Detailed representation of the genomic, methylomic, transcriptomic, proteomic, and phosphoproteomic profiles across different medulloblastoma subtypes (WNT, SHHα, SHHβ, G3, G4α, G4β, and G4γ). The upper sections highlight the frequency of genomic alterations, including point mutations, amplifications, and deletions. The lower sections display RNA markers, DNA methylation patterns, RNA expression, protein expression, and protein phosphorylation levels, indicating subtype-specific molecular signatures and pathway activations. SHH, sonic hedgehog.

To identify a more granular and clinically relevant molecular resolution of medulloblastoma classification, we applied an integrative approach that combined the multi-omics data from primary tumors^9^ (Fig. 1). This revealed a classification similar to, but more refined than, the canonical four subgroups (Supplementary Fig. 3 and Fig. 1). Our multi-ome clusters were consistent with the conventional four molecular subgroups, but differed from the clusters obtained by similarity network fusion clustering with proteome (Supplementary Fig. 3). Notably, our multi-ome clusters showed significant associations with clinical relevance (Fig. 1). The SHH group was further subdivided into two distinct subtypes, whereas group 4 was resolved into three subtypes, each associated with markedly different prognostic outcomes. Within the SHH group, we identified two distinct subtypes, including SHHα and SHHβ, with SHHβ demonstrating high similarity to group 4 (Supplementary Figs. 2 and 3). Owing to the limited number of samples, further subclassification of group 3 was not feasible. Conversely, we uncovered distinct variability within the group 4 tumors, identifying three dominant subtypes, including G4α, G4β, and G4γ. To validate our proteogenomic classification, we compared our results using a public data set^6,10^ with other omics platform (Supplementary Figs. 4 and 5). Our subtypes showed strong overall correlation with previously defined proteome subgroups, including concordance of SHH subdivisions and separation of group 4 into multiple clusters (Supplementary Fig. 4). Our subtype analysis revealed known clinical characteristics: G4a exhibited a high male predominance, whereas the WNT subtype was found primarily in older children. Some discordance was observed in SHH assignments, highlighting biological complexity and methodological differences between proteome-based taxonomies.

Whole-exome and whole-genome sequencing of our cohort identified subtype-specific driver aberrations that are consistent with prior studies^5,11^. WNT tumors carried CTNNB1 mutations and chromosome 6 monosomy, whereas SHH tumors harbored PTCH1 and SUFU alterations, with occasional germline variants. We identified germline ELP1 and SUFU variants within SHH subtypes, as well as three BRCA2 variants in our cohort. The characterization of transcriptome of our classified subtypes confirmed that patterns established four subgroup-defining marker gene sets^12^ (Fig. 1 and Supplementary Fig. 6).

**Fig. 2:**
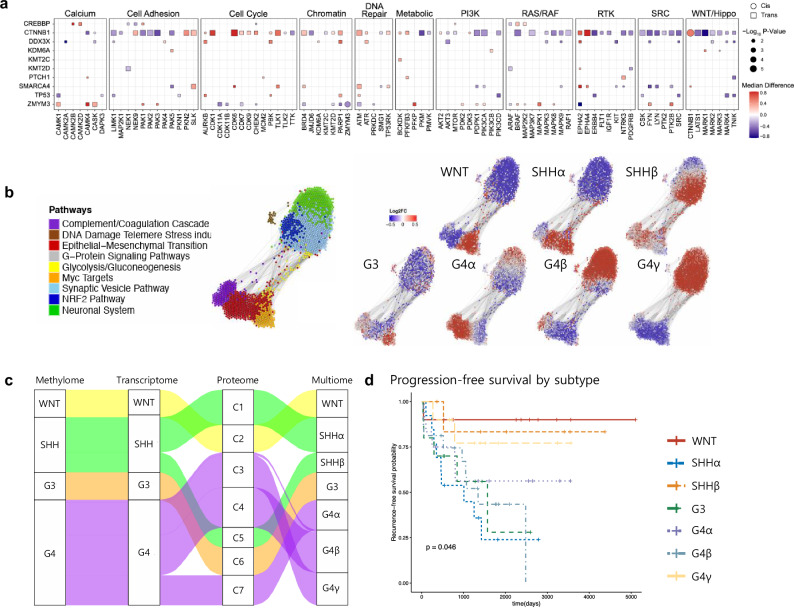
Characterization of seven subtypes. **a** The functional impact of genomic alterations on protein abundance. **b** Visualization of protein function and expression patterns for each medulloblastoma subtype using a protein–protein correlation network. The networks are color-coded by distinct pathways, the activity of which are higher in each specific molecular subgroup. **c** Sankey diagram illustrating the migration of samples through clustering of different molecular layers. **d** Kaplan–Meier curve depicting progression-free survival for patients stratified by medulloblastoma subtype (*P* = 0.046 by stratified log-rank test). RTK, receptor tyrosine kinase; SHH, sonic hedgehog.

Next, we systematically investigated the functional impact of genomic alterations on protein abundance at both *cis*-acting and *trans*-acting levels (Fig. 2a). Notably, *cis*-effects were most prominent for CTNNB1 alterations, which led to significantly increased protein abundance in WNT and cell-cycle pathways. According to the WHO Classification of Tumours of the Central Nervous System (5th edition), CTNNB1 mutation is the defining molecular hallmark of WNT-activated medulloblastoma and is recognized as an essential diagnostic criterion. By contrast, loss-of-function mutations in ZMYM3, a chromatin-associated transcriptional regulator, were associated with marked reduction in cell-cycle and chromatin-related pathway activities, suggesting impaired protein stability and translation, whereas it promoted increased calcium channel and PI3K signaling pathways.

The functional profiles of the proteins for the subtypes are summarized in Fig. 1 and Supplementary Fig. 7. Then, we investigated the proteomic profiles of each subtype based on a protein–protein interaction network. Proteins that were co-expressed clustered into functionally coherent modules (Fig. 2b). Projecting the abundance of subtype-specific proteins onto the network revealed a clear pattern in which uniquely enriched proteins were localized within functionally relevant clusters. For example, G4α-specific proteins were predominantly concentrated in epithelial–mesenchymal transition (EMT) and coagulation cluster regions, whereas G4γ subtype proteins were concentrated within the neuronal system in the network (Fig. 2b). We further validated such findings using public proteogenomic data sets, which showed similar functional clustering patterns (Supplementary Fig. 8). These results suggest that subtype-specific proteins undergo distinct functional modulation within the protein*–*protein interaction network. SHHβ tumors shared functional signatures with G4γ, including axon guidance and synaptic processes, whereas SHHα tumors were enriched for cell-cycle-related pathways. These results align with recent proteomic subgroup descriptions^10^. Conversely, the SHHα subtype was enriched with cell-cycle-related functions^13^.

We tracked the re-clustering of samples across different molecular layers (Fig. 2c and Supplementary Fig. 3). Although the four conventional subgroups were consistently maintained in the methylome and transcriptome clusters, proteome-based classification identified seven distinct multi-omic clusters. Survival analysis based on progression-free survival (PFS) rate demonstrated that the WNT, SHHβ, and G4γ subtypes were associated with favorable clinical outcomes, whereas other subtypes invariably showed worse survival probabilities (*P* = 0.046, Fig. 2d). Overall, these findings suggest that the newly defined proteogenomic-driven subtypes mark clinical relevance in medulloblastoma progression.

### Medulloblastoma subtypes are associated with neuronal differentiation

**Fig. 3:**
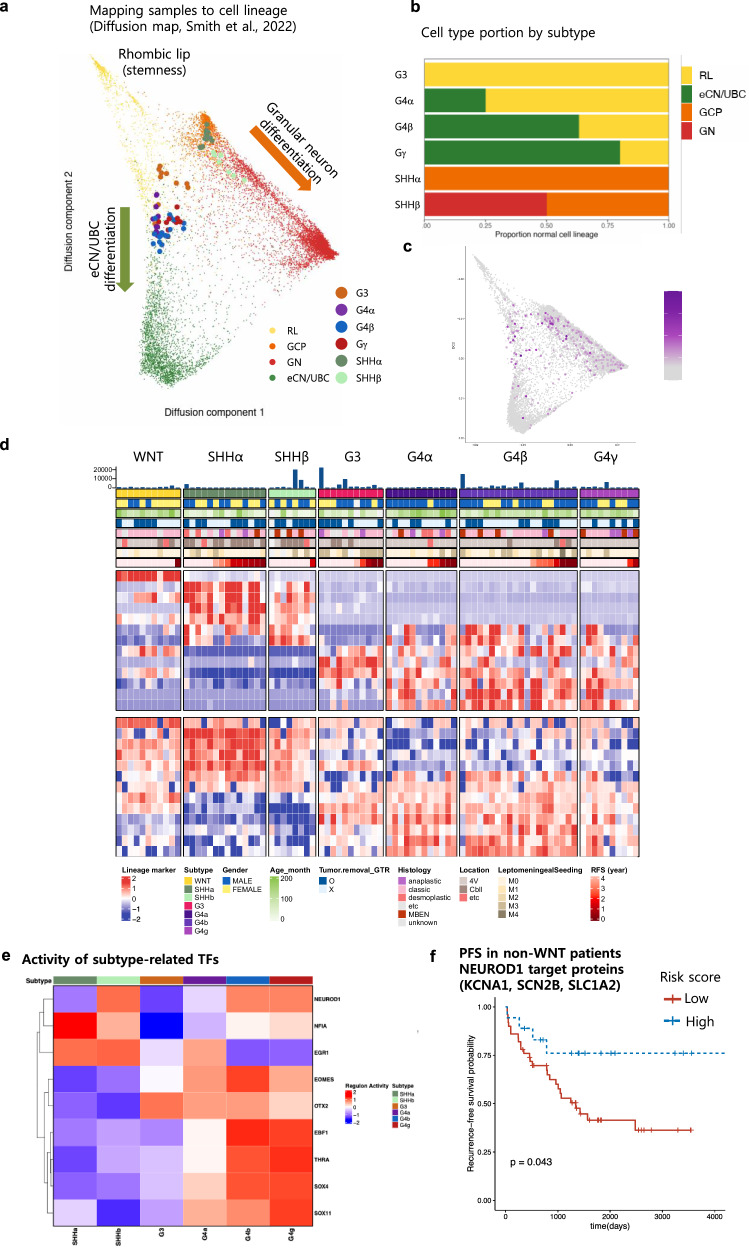
Medulloblastoma subtypes in the context of normal cerebellar development and differentiation. **a** Mapping of medulloblastoma subtypes on single-cell RNA-sequencing data of normal cerebellar cellular differentiation using a Diffusion map. The plot shows different medulloblastoma subtypes with respect to normal neuronal differentiation from RL (stemness) to GN/UBC differentiation. **b** Proportion of neuronal cell types within each medulloblastoma subtype. The bar chart visualizes the composition of neuronal cell lineages (RL, GCP, eCN/UBC, and GN) across different medulloblastoma subtypes. **c** Expression patterns of a representative neuronal differentiation marker (NEUROD1) on Diffusion map. **d** Expression patterns of representative lineage markers across primary medulloblastoma subtypes. **e** Activity of subtype-related TFs calculated with the target protein expression. **f** Progression-free survival (PFS) difference by NEUROD1 target protein expression in patients with non-WNT medulloblastoma (*P* = 0.043 by log-rank test). eCN, excitatory cerebellar nuclei neuron; GCP, granule cell progenitor; GN, granular neuron; RL, rhombic lip; SHH, sonic hedgehog; TF, transcription factor; UBC, unipolar brush cell.

We next mapped our bulk samples onto single-cell RNA-seq developmental trajectories from the human cerebellum^14,15^ (Fig. 3a). Diffusion mapping of rhombic lip (RL), granule cell progenitor (GCP), granular neuron (GN), and unipolar brush cell (UBC) lineages identified two major axes of differentiation. SHHα and SHHβ tumors localized along the GN axis, with SHHβ tumors positioned closer to differentiated GN states. Group 3 tumors localized near RL, consistent with their progenitor-like state, whereas G4α, G4β, and G4γ tumors aligned along the UBC axis, with a gradient of differentiation (Fig. 3a). These mappings of bulk data support a developmental continuum across subtypes and also integrate well with previous single-cell-based medulloblastoma studies^8^. We also mapped the 12 WHO subtypes of our patient samples onto the Diffusion map of the single-cell RNA-sequencing (scRNA)-seq data (Supplementary Fig. 9). These subtypes were also aligned along the GN and UBC axes. On the basis of the Diffusion map, we calculated the proportion of neuronal cell types within each medulloblastoma subtype (Fig. 3b). The results showed that G3 tumors were exclusively composed of RL-type cells, whereas SHHα samples were identified with GCP-type cells. Conversely, SHHβ tumors included a mixture of GCP and differentiated GN-type cells. The three group4 subtypes showed a gradient pattern composed of both RL-type and UBC-type cells. To validate these findings, we used scRNA-seq data to calculate the proportions of cell lineage types in medulloblastoma tissues^16^ (Supplementary Fig. 10). We also calculate the proportions of cell lineage type of each patient (Supplementary Fig. 11). The consistency in cell-type distributions across both data sets supports the robustness of our lineage tracing analyses. Our proteogenomic classification provides a refined resolution of the current consensus system, offering novel fine-tuning beyond the four canonical subgroups. In direct comparison with the clinically implemented WHO classification — which is primarily based on DNA methylation profiling — our integrative multi-omics approach demonstrates superior alignment with the developmental biological origins of these tumors. In particular, our analysis resolves SHH tumors into two distinct entities, SHHα and SHHβ, with clearer separation than afforded by methylation-based clustering alone. This refined distinction is biologically meaningful, as the SHHα and SHHβ subtypes map to different points along the GN progenitor differentiation trajectory and exhibit divergent clinical outcomes. Furthermore, the improved resolution achieved by our classification underscores its clinical relevance, offering a framework that better captures both the developmental lineage and prognostic heterogeneity of medulloblastoma. Conventional SNFCC-based clustering of our multi-omic data set also produced mixed results when mapped to developmental trajectory (Supplementary Figs. 4 and 5).

Previous studies showed that medulloblastoma subgroups are characterized by unique expression patterns of transcription factors (TFs) known to regulate neuronal development^17,18^. We visualized the transcriptional patterns of representative lineage-specific TFs on the Diffusion map (Fig. 3c and Supplementary Fig. 12). The TF profiles revealed distinct features for each medulloblastoma subtype: (1) OTX2-positive cells were aligned along the eCN/UBC axis and overlapped with Group 3 and Group 4 medulloblastoma tumors; (2) GLI2-positive cells were localized along the GN axis and overlapped with SHH medulloblastoma tumors; (3) NEUROD1-positive cells were associated with non-WNT medulloblastoma tissues. We further examined the RNA and protein expression patterns of these lineage-specific TFs based on the new subtypes, identifying subtype-specific expression profiles (Fig. 3d). For instance, PAX3 characterized the WNT subgroup, GLI1 and GLI2 were predominantly active in the SHH subgroup, and OTX2 constituted the unique transcriptional profiles of groups 3 and 4. We then nominated the TF profile that best characterizes the regulons of each subtype (Fig. 3e). NEUROD1 activity was strongly enriched in SHHβ, G4β, and G4γ, consistent with their more differentiated states.

As we found that NEUROD1-positve cells overlapped with non-WNT medulloblastoma tumors on the Diffusion map, we sought to explore the significance of this association. NEUROD1 is a well-known TF that regulates neuronal differentiation^14^. Using publicly available ChIP-seq data, we defined NEUROD1 target genes, which were highly expressed in SHHβ and G4γ (ref. ^19^) (*P* = 0.0078, Supplementary Fig. 13a). Next, we calculated correlation coefficients between NEUROD1 RNA and protein expression levels with its target genes (Supplementary Fig. 13b). Comparative analysis of the correlation coefficients between the neuronal subtype-related proteins and other target genes revealed that the abundance of the neuronal subtype-related proteins was significantly correlated with NEUROD1 expression compared with other target genes (Supplementary Fig. 13b,c). We next calculated the activity of subtype-related TFs in each subtype based on the expression of their target genes (Fig. 3e). Notably, NEUROD1 activity was high in neuronal subtypes, SHHβ, G4β, and G4γ. Additionally, NEUROD1 RNA expression conferred favorable clinical outcomes in patients with non-WNT medulloblastoma (SHHα, SHHβ, G3, G4α, G4β, and G4γ) (Supplementary Fig. 13d,e). PFS analysis further showed that the expression of NEUROD1 target was significantly associated with improved prognosis in patients with non-WNT medulloblastoma (Fig. 3f). This trajectory analysis of our medulloblastoma subtypes using scRNA-seq is consistent with previous findings in the SHH subgroup^13^. Collectively, these analyses highlight that medulloblastoma subtypes reflect specific developmental trajectories and regulon activities. The proteogenomic subtypes we identified not only align with lineage differentiation but also carry prognostic value, linking neuronal maturation state with clinical outcome.

### Proteogenomic analyses unveil targets and markers for SHH subtypes

**Fig. 4:**
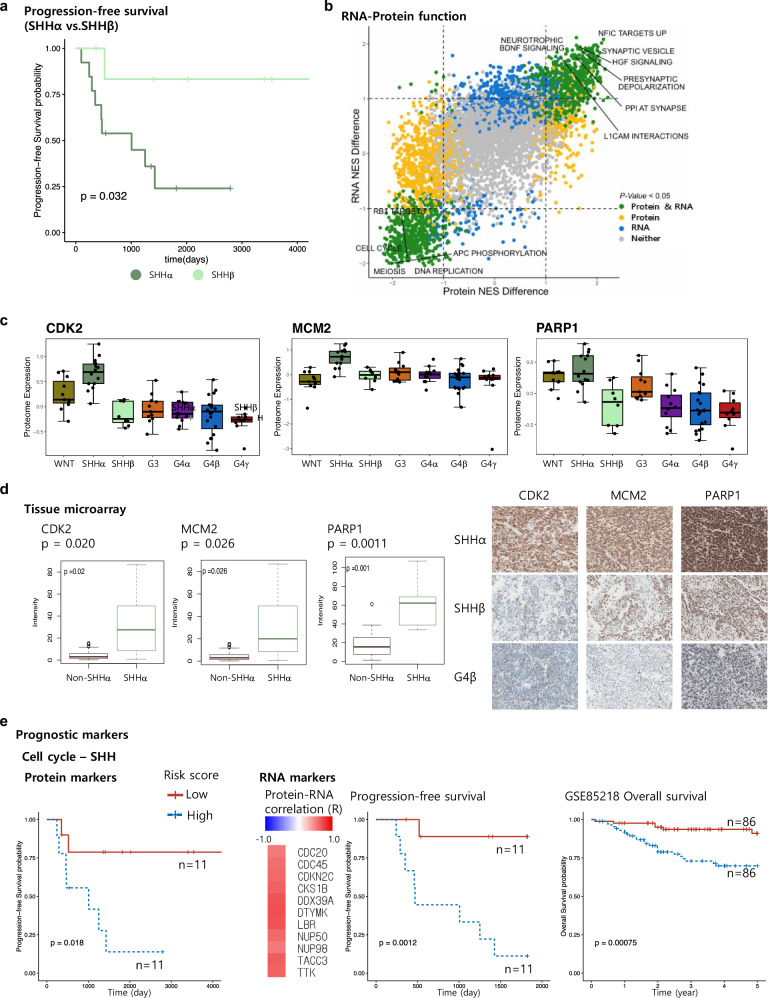
Comparative analysis of SHHα and SHHβ subtypes in RNA and protein expression (prognosis). **a** Progression-free survival difference between SHHα and SHHβ subtypes. **b** Selection of RNA and protein functions enriched in SHHα and SHHβ subtypes (normalized enrichment score (NES)). **c** Expression levels of representative proteins in SHHα subtype. **d** Validation of SHHα-specific protein expression using tissue microarray. **e** Key prognostic protein and RNA markers exhibiting high correlation, identified using the Cox proportional hazards model. BDNF, brain-derived neurotrophic factor; SHH, sonic hedgehog; CDK2, cyclin-dependent kinase 2; MCM2, mini-chromosome maintenance complex component 2; PARP1, poly(ADP-ribose) polymerase 1 .

The two newly defined subtypes, SHHα and SHHβ, exhibited distinct clinical outcomes in patients with SHHβ, demonstrating a significantly better prognosis compared with the SHHα group (Fig. 4a). Functional analysis showed that RNA and proteins implicated in cell cycle and DNA replication accumulated in the SHHα subtype, whereas neuronal proteins were expressed in the SHHβ tumors. Moreover, the expression levels of proteins and corresponding transcripts within these functional categories were highly correlated (Fig. 4b and Supplementary Fig. 14). Specific cell-cycle-related proteins, including CDK2, MCM2, and PARP1, were identified as representative SHHα markers (Fig. 4c and Supplementary Fig. 15). To determine whether these proteins could serve as reliable biomarkers within clinical frameworks, we sought to validate their abundance in histology sections of medulloblastoma from tissue microarray (Fig. 4d and Supplementary Fig. 16). Consistent with our findings, a significant upregulation of CDK2, MCM2, and PARP1 was observed in the SHHα subtype (Fig. 4d).

Using a Cox proportional hazard (CoxPH) model, we identified several core proteins with subtype-specific functions that were significantly associated with poor prognosis in patients with medulloblastoma (Fig. 4e). We then leveraged the results of our correlation analysis to select RNA markers that affect protein functions. Using selected RNA markers, we calculated a risk score using the CoxPH model and performed survival analysis. As a result, we discovered that 11 RNA markers representing cell cycle and metabolism categories were significantly associated with poor prognosis of patients with SHH medulloblastoma (cell cycle: *P* = 0.01, *n* = 21; RNA metabolism: *P* = 0.001, *n* = 21) (Fig. 4e). To validate these findings, we analyzed the overall survival from a public data set (GSE85217), which also showed that the same RNA markers were significantly associated with poor prognosis in patients with SHH medulloblastoma (cell cycle: *P* = 0.001, *n* = 198; RNA metabolism: *P* = 0.001, *n* = 198) (Fig. 4e). Considering that ~90% of patients with relapsed medulloblastoma eventually succumb to the disease, it is reasonable to assume that PFS outcomes closely reflect overall survival^1–3^. Collectively, the prognostic subtype-specific markers identified through our integrated multi-omics analysis emerged as candidate biomarkers for clinical applications. They may also be useful to stratify patients with medulloblastoma following identification of subtype-specific vulnerabilities.

### Phosphoproteome analyses characterize SHHα-specific kinases

**Fig. 5:**
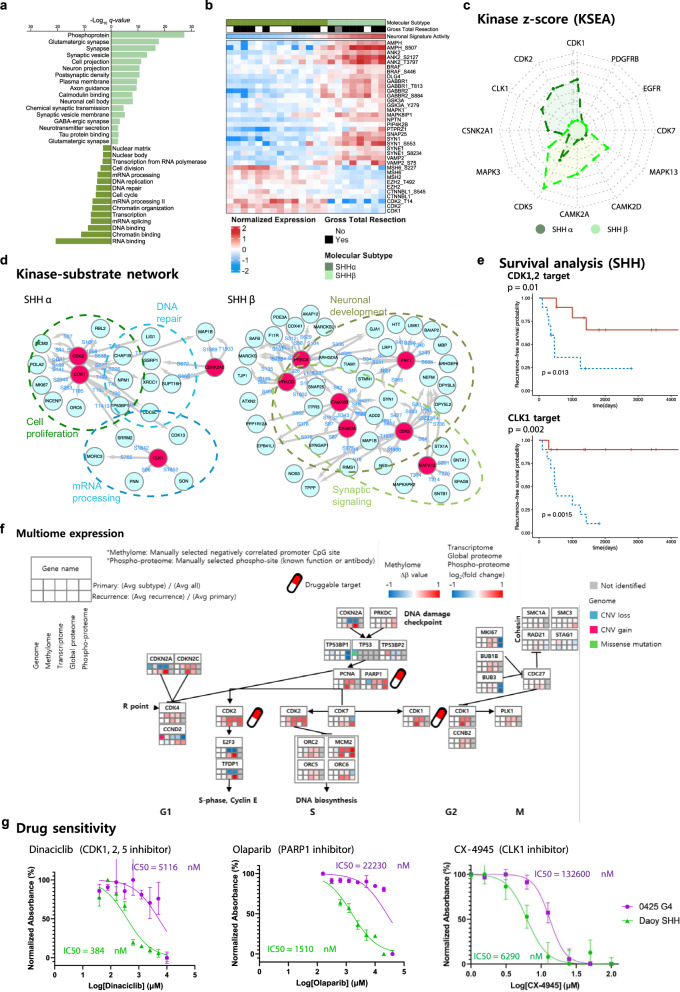
Difference between SHHα and SHHβ subtypes in protein phosphorylation (therapeutics). **a** Enriched function of phosphorylated proteins in SHHα and SHHβ subtypes. **b** Representative phosphorylated proteins in SHHα and SHHβ subtypes. **c** Different kinase activity between SHHα and SHHβ (KSEA *z*-score). **d** Kinase–substrate network in SHHα and SHHβ subtypes. **e** Associations between progression-free survival and the phosphorylation of subtype-specific kinase target proteins (CoxPH model, CDK1, 2 targets: *P* = 0.01, CLK1 targets: *P* = 0.002 by log-rank test). **f** Multi-ome profile on cell-cycle pathway in SHHα subtype. **g** Comparison of drug sensitivity between SHH and group 4 cell lines (green: SHH (Daoy) cell line, purple: group 4 (0425) cell line). CNV, copy number variant; KSEA, kinase–substrate enrichment analysis; PARP, poly(ADP-ribose) polymerase; SHH, sonic hedgehog.

Our multi-omics approach integrated several layers of molecular profiles, including the phosphoproteome. We initially compared the phosphoproteome between SHHα and SHHβ subtypes (Fig. 5a, b and Supplementary Fig. 17a). In the SHHα subgroup, both cell cycle and DNA replication-related phosphoproteins, such as CDK2 and EZH2, were markedly enriched, whereas the SHHβ tumors exhibited enrichment of synapse-related phosphoproteins, including SYN1 and GABBR1. To identify subtype-specific phosphoproteins and upstream kinases, we performed a kinase activity-based phosphoproteomic analysis (Fig. 5c, d). Kinase–substrate enrichment analysis (KSEA) revealed distinct subtype-specific kinases and their substrate phosphoproteins (Fig.5c). Using these representative kinase–substrate interactions, we constructed subnetwork modules highlighting the core subtype-specific kinases and their target substrates with their known functional roles (Fig. 5d). The resulting subnetworks were centered around key kinases, forming subtype-specific protein functional groups. Specifically, CDK1/2 and their substrates constituted a cell-cycle-related module in the SHHα subtype, whereas CLK1 and its substrates composed an mRNA processing-related module. Next, we evaluated whether the identified functional modules were associated with medulloblastoma progression (Fig. 5e). We used the CoxPH model to generate a risk score based on the phosphorylation levels of CDK1/2 and CLK1 target proteins. Patients were then stratified into high-risk and low-risk groups based on this score, and statistical significance was assessed using Kaplan–Meier plots and the log-rank test. This analysis aimed to examine the association between the phosphorylation of CDK1/2 and CLK1 target proteins and patient prognosis. Previous analyses showed that phosphorylation of these target proteins is elevated in SHHα tumors. The current analysis demonstrates that increased phosphorylation of these proteins is statistically associated with poor prognosis. We found that the phosphorylation of proteins modulated by subtype-specific kinases, such as CDK1/2 and CLK1, was significantly associated with PFS in each subgroup. These findings indicate that targeted inhibition of subtype-specific kinases may regulate the phosphorylation of key effector proteins, potentially altering disease progression and offering new therapeutic opportunities.

We further examined the multi-omic profiles of core proteins in SHHα medulloblastoma within known signaling pathways (Fig. 5f). Core proteins, including CDK1, CDK2, and PARP1, were predominantly involved in the cell-cycle pathway. Interestingly, although most core proteins lacked somatic gene alterations, except for TP53, CCND2, and CDKN2A, they showed substantial RNA and protein expression changes, indicating transcriptional regulation of corresponding pathways. Given their continuous upregulation during disease progression and their enzymatic activities as kinase or DNA-damage response regulators, we selected CDK1, CDK2, and PARP1 as potential therapeutic targets for the SHHα subtype. Additionally, phosphorylation of CDK1 and PARP1 was consistently elevated during progression, indicating functional activation of these enzymes. We speculate that inhibition of these core kinases could disrupt the subtype-specific kinome and downregulate related protein functions within the subnetwork. We also identified positive correlations between kinase protein expression and target substrate phosphorylation, further supporting the potential for targeted kinase inhibition to modulate subtype-specific signaling pathways (Fig. 5g and Supplementary Fig. 17b). These results highlight that subtype-specific kinase targeting may alter the phosphorylation state of effector proteins, which drive medulloblastoma progression and constitute promising therapeutic implications.

### Synaptic signaling proteins are associated with good prognosis of medulloblastoma

**Fig. 6:**
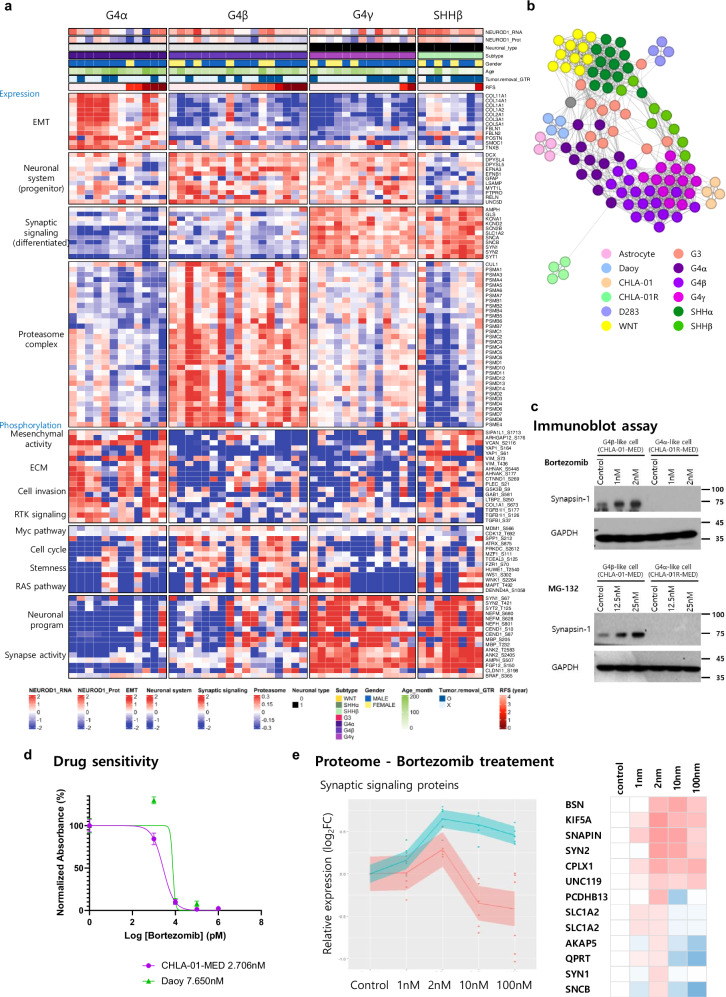
Proteomic features of group 4 subtypes. **a** Representative protein expression and phosphorylation patterns of G4α, G4β, G4γ, and SHHβ. **b** Identification of subtypes within medulloblastoma cell lines in sample–sample correlation network using proteome data. **c** Recovery of a neuronal differentiation marker (Synapsin-1, Syn1) by proteasome inhibitor treatment (immunoblot assay). **d** Proteasome inhibitor sensitivity of a group 4 cell line (CHLA-01-MED cell, G4β-like). **e** Expression patterns of representative synaptic signaling proteins by dose-dependent bortezomib treatment (liquid chromatography and mass spectrometry). ECM, extracellular matrix; EMT, epithelial–mesenchymal transition; FC, fold change; RTK, receptor tyrosine kinase; SHH, sonic hedgehog.

Our multi-omics clustering and functional annotation revealed that proteins implicated in neuronal and synaptic functions accumulated at high levels in three medulloblastoma subtypes, SHHβ, G4β, and G4γ (Fig. 1). Previous studies using proteomic data reported that SHHβ tumors contained high levels of glutamatergic synaptic proteins and were more similar to tumors in group 4 than those in the SHHα subtype^10^. Accordingly, our unsupervised clustering showed that the SHHβ subtype clustered with group 4 tumors, particularly the G4γ subgroup, and neuronal proteins were enriched in both groups compared with G4α and G4β tumors (Fig. 6a). The neuronal proteins elevated in SHHβ were primarily involved in synaptic signaling and were typically expressed in differentiated neural cells (Fig. 6a). Principal component analysis further distinguished group 4 patients into three subtypes, G4α, G4β, and G4γ (Supplementary Fig. 18a). Interestingly, the two neuronal subtypes, SHHβ and G4γ, both enriched in synaptic signaling proteins, were significantly associated with favorable prognosis in non-WNT medulloblastoma (Supplementary Fig. 18b, *P* = 0.015 by log-rank test). Moreover, risk scores based on a CoxPH model using 11 synaptic transmission proteins showed a significant association with a better clinical outcome (Supplementary Fig. 18c, *P* = 0.0006 by log-rank test). Together, these findings suggest that tumor cells expressing synaptic transmission proteins belong to more differentiated and less aggressive tumors that may require more conservative treatment.

We also found that the expression of the proteasome complex was higher in the G4β subtype, suggesting that differences among G4α, G4β, and G4γ subtypes may arise not from transcriptional regulation but from post-transcriptional mechanisms, such as translation and proteasomal degradation of synaptic signaling proteins (Fig. 6a). Next, we sought to validate experimentally the aforementioned findings. Toward this aim, we first determined the proteogenomic subtypes of medulloblastoma cell lines using LC-MS/MS-based proteome analysis (Fig. 6b). By comparing the proteomic profiles of medulloblastoma cell lines with those of the seven medulloblastoma subtypes we have identified, we assigned each cell line to a specific subtype. Next, we investigated whether proteasome inhibition regulated synaptic signaling (Fig. 6c). Immunoblot assays showed that treatment with the proteasome inhibitor bortezomib regulated the expression of Synapsin-1 (Syn1), a protein marker of synaptic signaling and neuronal differentiation. Specifically, Syn1 was not expressed in the G4α medulloblastoma cell line CHLA-01R-MED but was more abundant in the G4β/γ cell line CHLA-01-MED. Upon treatment with bortezomib, Syn1 further accumulated toward markedly higher levels, indicating that proteasome inhibition upregulated synaptic proteins. Finally, we assessed the therapeutic potential of proteasome inhibition in medulloblastoma. We measured the drug sensitivity of bortezomib in the G4β/γ medulloblastoma cell line (CHLA-01-MED) and showed that these cells were highly sensitive to nanomolar concentrations of bortezomib (Fig. 6d). Using LC-MS/MS-based proteome analysis, we investigated expression patterns of synaptic signaling proteins in response to varying concentrations of bortezomib in the G4β/γ medulloblastoma cell line (Fig. 6e). Our analysis revealed two distinct expression profiles among synaptic signaling proteins. In one group, protein expression increased following proteasome inhibition by Bortezomib. Conversely, the second group displayed upregulation at lower bortezomib concentrations but was downregulated at higher concentrations. These findings suggest that proteasome inhibition may restore synaptic signaling function, potentially leading to a less aggressive medulloblastoma subtype but it needs detailed treatment control for dose and concentration. However, precise control over bortezomib dosage and concentration is essential to optimize treatment outcomes.

### A receptor tyrosine kinase has therapeutic potential for group 4 patients

**Fig. 7:**
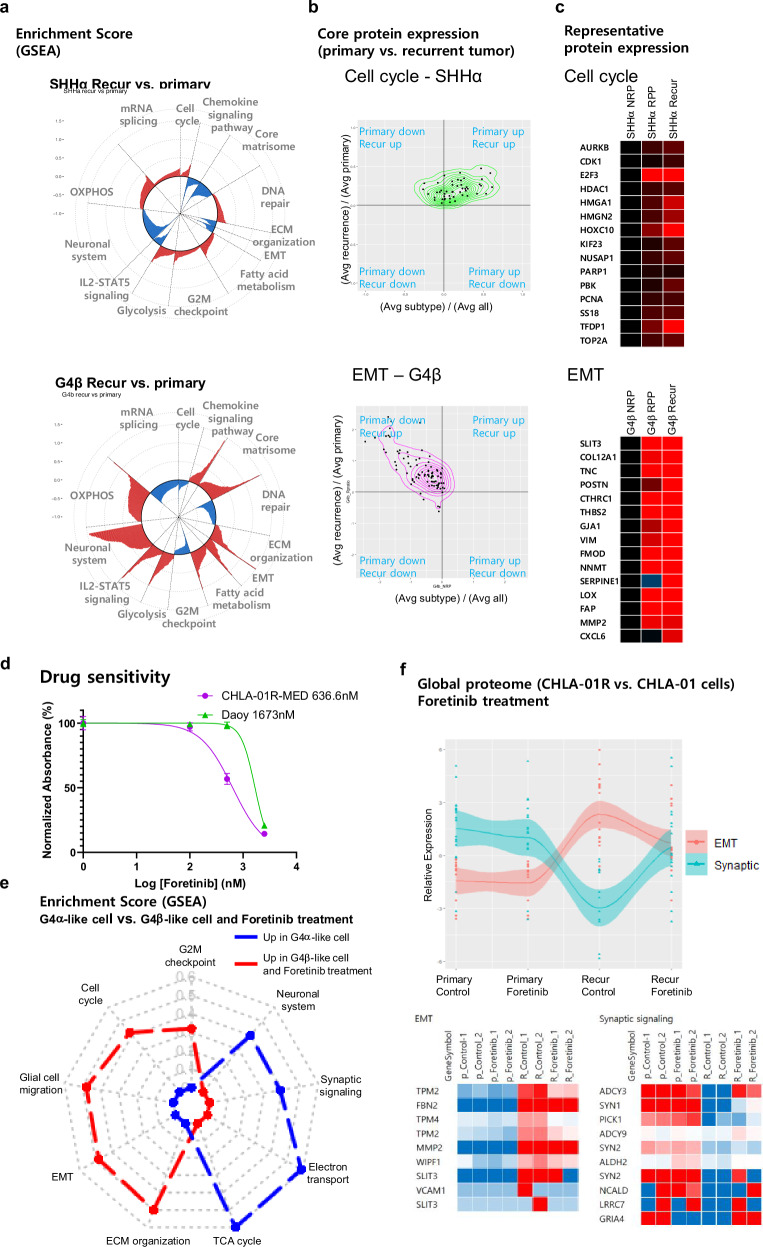
Subtype-specific protein changes during recurrence of medulloblastoma. **a** Changes (GSEA enrichment score) in enriched protein functions during recurrence. **b** Distribution of functional core protein expression (*x*-axis: change in primary tumors, *y*-axis: change during recurrence). **c** Expression patterns of representative core proteins during recurrence. **d** MET inhibitor sensitivity of a group 4 cell line (CHLA-01R-MED cell, G4α-like, recurrent). **e** Enriched functions of medulloblastoma cell lines with MET inhibitor treatment. **f** Protein function changes by MET inhibitor treatment (p: CHLA-01 cell (primary) and R: CHLA-01R cell (recurrence)). ECM, extracellular matrix; EMT, epithelial–mesenchymal transition; NRP, non-recurrent primary tumor; OXPHOS, oxidative phosphorylation; Recur, recurred tumor; RPP, recurrent-potent primary tumor; SHH, sonic hedgehog; TCA, tricarboxylic acid.

To identify the underlying mechanisms sustaining group 4 medulloblastoma, we focused on phosphoproteome data. First, we compared the phosphoproteomic landscape among G4α, G4β, and G4γ subtypes and identified subtype-specific phosphorylation activities linked to distinct functional pathways (Supplementary Fig. 19). In the G4α subtype, we found enrichments of proteins involved in EMT and receptor tyrosine kinase (RTK) signaling functions and transforming growth factor-β. The G4β tumors exhibited activation of Myc pathway and cell-cycle functions, including MDM1, SPP1, and ATRX. The G4γ subtype was characterized by synaptic activity and neuronal differentiation functions, including SYN1, SYN2, SYT2, and MBP. To further delineate group 4-specific phosphoproteins and their upstream kinases, we performed a kinase activity-based phosphoproteome analysis (KSEA) (Supplementary Fig. 19). By mapping representative kinase–substrate interactions, we constructed G4-specific subnetwork modules and annotated their functional roles (Supplementary Fig. 19). We found that G4α subtype was driven by RTK–JAK–STAT signaling, with PDGFRB and JAK2 at its core, whereas G4γ subtype was associated with neuronal functions, regulated by calcium/calmodulin-dependent protein kinases (CAMKs), protein kinase C isoforms (PRKCs), and mitogen-activated protein kinases (MAPKs). To evaluate the therapeutic potential of targeting these pathways, we evaluated the pharmacological sensitivity of RTK inhibitors (Fig. 7d). In particular, we tested the effect of foretinib, a Met kinase inhibitor, in the G4α medulloblastoma cell line CHLA-01R-MED. These experiments showed that the G4α medulloblastoma cell line was sensitive to nanomolar concentrations of foretinib, indicating that RTK inhibitors may be used as promising therapeutic strategies for patients with group 4 (G4α) medulloblastoma.

### Medulloblastoma subtypes show different patterns of recurrence

Tumor recurrence remains therapeutically unresolved in medulloblastoma owing to its complex evolutionary trajectory. To investigate its dynamic process, we conducted longitudinal molecular profiling of medulloblastoma by analyzing 10 cases with matched primary and recurrent tumor pairs (Supplementary Fig. 20). Initially, we compared the genome alterations between primary and recurrent tumors (Supplementary Fig. 20). Unsupervised hierarchical clustering and cell–cell correlation network analysis of longitudinal medulloblastoma revealed extensive intertumoral heterogeneity, defined by clustering of matched primary and recurrent tumors from the same patients (Supplementary Figs. 21 and 22). This finding indicates that the molecular difference between primary and recurrent tumor is marginal, and recurrence is not associated with a major subtype switch. Next, we categorized primary tumors into two groups: non-recurrent primary (NRP) tumors from patients without recurrence, and recurrent-potent primary (RPP) tumors from patients with recurrence. Using proteomic data, we calculated the average protein expression levels in NRPs, RPPs, and recurrent tumors and analyzed protein expression patterns that changed with recurrence (Supplementary Fig. 23). The results showed that most RPPs and their corresponding recurrent tumors exhibited consistent changes in protein expression, with upregulated proteins often being subtype-specific.

To investigate recurrence-associated changes across medulloblastoma subtypes, we analyzed recurrence-related protein functions (Fig. 7a and Supplementary Fig. 24). Each subtype of primary tumor was specifically enriched in specific protein functions, including cell-cycle relation in the SHHα subtype, EMT and extracellular matrix reorganization functions in the G4α subtype, and neuron-related functions were enriched in the G4β and SHHβ subtypes (Fig. 1). We specifically focused on the recurrence dynamics of SHHα and G4β as they were the dominant recurrent subtypes. By comparing the protein functions between NRP and RPP, we identified recurrence-specific protein functions (Fig. 7a and Supplementary Fig. 24). In the SHHα subtype, the primary tumors were enriched in the cell cycle, mRNA processing, and DNA repair functions, which became progressively more enriched as SHHα tumors progressed from NRP to recurrent tumors (Fig. 7a). By contrast, the G4β subtype exhibited a shift from neuron-related functions in primary tumors to EMT and extracellular matrix activities in recurrent tumors, thus converting to G4α-like phenotype. Next, we examined core protein expression dynamics in relation to primary and recurrence (Fig. 7b, c and Supplementary Fig. 25). We hypothesized two independent axes: the *x*-axis represents the expression of each core protein in the primary subtype, and the *y*-axis represents the expression changes at recurrence. Plotting core proteins related to cell cycle, EMT, and neuronal function based on expression values revealed that cell-cycle functions, including the accumulation of CDK1 and MCM2, were enriched in the SHHα subtype, whereas the core proteins related to EMT functions, including the accumulation of POSTN and TGM2, were observed in the G4β subtype (Fig. 7b, c and Supplementary Fig. 25). These results suggest that both EMT and neuronal functions are essential for recurrence in the group 4 subgroup, and a deficiency in these functions may need to be compensated during tumor relapse. Moreover, primary-recurrent pairs were clustered together, indicating that the neuronal cell types remained stable based on transcriptome data.

Finally, we sought to experimentally validate our findings in two group 4 medulloblastoma cell lines: G4β-like (or G4γ-like), CHLA-01-MED, and G4α-like, CHLA-01R-MED. The CHLA-01-MED cell line was characterized by neuronal and synaptic signaling functions, whereas the CHLA-01R-MED cell line was characterized by EMT-associated characteristics. Notably, these two cell lines are primary-recurrence pairs derived from the same patient. Constructing a cell-to-cell interaction network with proteome data allowed us to further refine the subtyping of medulloblastoma cell lines (Supplementary Fig. 26). Kinase-substrate network analysis revealed that MET serves as a central hub, coordinating multiple oncogenic programs including RTK–JAK–STAT signaling and focal adhesion. To uncover the therapeutic effects of Met kinase inhibition shown in Fig. 7d, we performed LC-MS/MS-based proteome analysis after treating the medulloblastoma cell lines with Foretinib (Fig. 7d–f and Supplementary Fig. 27). Strikingly, our results highlighted a dual function of Met inhibition on group 4 medulloblastoma. In the aggressive G4α-like medulloblastoma cells, EMT functions were suppressed. Moreover, synaptic signaling function, which was initially low in G4α-like cells, was restored to the levels observed in G4β-like or G4γ-like medulloblastoma cells. This suggests that Met inhibition not only reduced EMT activity but also promotes differentiation toward a neuronal subtype. Consequently, we propose that targeting proteome and kinome to modulate neuronal differentiation represents a promising therapeutic strategy for treating medulloblastoma.

## Discussion

Many omics studies have classified medulloblastoma into four subgroups, WNT, SHH, group 3, and group 4, which currently represent the global standard of medulloblastoma classification^4^. By integrating proteomics data with other existing omics data, several reports described new subtypes, each of which is sustained by subtype-related pathways and activities proposed as potential therapeutic targets^10,20^. In spite of many attempts to integrate proteomics data, the existing global standard has not been improved. This is due to relatively limited numbers of samples, the scale of the multi-omics analysis platform, and inconsistency in analysis methods. We generated data sets from five omics platforms, including proteomics data from ~120 tumor samples of medulloblastoma from ~90 patients. In our study, the numbers of patients, samples, and data types are larger than those of previous proteomics-integrating medulloblastoma omics study. Moreover, our sample collection contains not only primary tumors but also matched recurrent tumors. We compared our results with those of other medulloblastoma studies, validated their results, and identified new perspectives and types of data with more statistical power than other proteogenomic studies. Our analyses have conceptual novelty: (1) defining functionally distinct subtypes, (2) identifying targetable pathways for each subtype and linking them to candidate drugs, and (3) demonstrating developmental origins of subtypes through integration with neurodevelopmental trajectories. Our study provides a more advanced framework for understanding medulloblastoma biology and for therapeutic translation compared with previous studies.

Our findings enhance the reliability of existing results. First, our unsupervised clustering of methylomic and transcriptomic data well matched the conventional four subgroups, WNT, SHH, and groups 3 and 4 (Supplementary Figs. 4 and 5). By integrating proteomic data, we subdivided the SHH subgroup into SHHα and SHHβ subtypes. Archer et al. also subdivided the SHH subgroup into SHHα and SHHβ subtypes, which showed similar functional enrichment in the proteome, such as RNA processing, MYC targets, and DNA repair in SHHα, and synaptic signaling and axon guidance in SHHβ^10^. They also found that SHHβ was closer to group 4 than SHHα in proteome clustering. Similarly, according to our unsupervised clustering of proteomic data, SHHβ was most closely related to the G4β subtype (Supplementary Fig. 2). The similarity between our data and those reported in previous studies provides an accurate independent validation to our new findings. The detection of the SHHβ subtype characterized by a synaptic signature at the multi-omic level is not novel; however, it demonstrates that our analysis is consistent with previous findings^10,21^. Our primary concern lies in the fact that both SHHb and G4g subtypes are associated with good prognosis of recurrence, suggesting that patients within these groups may benefit from de-escalation of therapeutic intensity. Conversely, for the cell-cycle-driven SHHa subtype, we propose specific cell-cycle-targeting drugs and have validated the subtype-specific drug response in our study.

We were able to extend the scope of our investigation to recurrence in medulloblastoma as we generated omics data from matched recurrent tumor tissues. Although the prognosis of relapsed patients is substantially poorer than for patients with primary tumors, most proteogenomic studies of medulloblastoma have been restricted to primary tumors. By analyzing the functions that were enriched during recurrence, we found that the trends in protein functions were different among subtypes, especially between SHH and group 4 (Fig. 7). In the SHHα subtype, the protein functions of primary tumors were enriched in cell-cycle-related functions, including G2/M checkpoint, DNA replication, and repair, and these functions are further enriched during recurrence. We note that our study analyzed markedly more samples classified as group 4 medulloblastoma than were available in the study of Archer et al. Therefore, we were able to examine group 4 more closely and subdivide it into the G4α, G4β, and G4γ subtypes. The protein expression profiles of G4α showed a significant enrichment for EMT-related functions, whereas G4β and G4γ proteomes were enriched in neuron-related functions. At recurrence, the functional proteomic profile of G4β switched to the activation of EMT-related functions (Fig. 7). From these findings, we suggest that medulloblastoma recurrence is marked by a convergence toward mesenchymal transition.

Additionally, we compared the average gene expression differences between primary and recurrent tumor samples in medulloblastoma and glioblastoma (GBM) (Supplementary Fig. 28). The left half of the plot represents medulloblastoma, whereas the right half represents GBM. Each dot represents the average difference in gene expression levels (absolute values) between primary and recurrent tumor samples for individual genes. The radial distance from the center indicates the magnitude of the difference, with greater distances representing larger changes in average expression. The colors differentiate between the two tumor types, with medulloblastoma shown in green and GBM in red. The circular layout visually compares the expression variability and the magnitude of gene expression alterations between the two tumor types.

Performing lineage tracing analysis with public scRNA-seq data, we have identified that medulloblastoma subtypes display two distinct lineage-specific neuronal differentiation patterns (Supplementary Fig. 29): GN differentiation associated with the SHH subtype and UBC differentiation linked to group 4. Along the GN differentiation axis, the SHHβ subtype with more favorable prognostic outcomes exhibits a higher degree of neuronal differentiation compared with SHHα. The transcriptional regulatory mechanisms involve well-established lineage-related TFs, notably NEUROD1. By contrast, along the UBC differentiation axis, transcriptomic data alone cannot distinctly separate the three subtypes of group 4. However, proteomic and kinomic features successfully differentiate G4γ with better prognostic outcomes, from G4α and G4γ. Similar to SHHβ, G4γ shows a higher degree of neuronal differentiation compared with G4α and G4γ, but the regulatory mechanisms involve proteomic and kinomic features, such as proteasome activity and RTK signaling. This suggests that multi-omic regulatory compensation, rather than transcriptome alone, is pivotal for neuronal differentiation and prognosis, highlighting the importance of proteome and kinome in these processes.

Proteins are more functionally meaningful molecules than DNA or RNA because they directly serve as enzymes, receptors, structural elements such as the cytoskeleton, and more. If alterations in DNA or RNA are not linked to protein functions, they may not drive phenotypic changes. In comparing the G4β and G4γ subtypes, we did not identify significant transcriptomic differences. However, several synaptic signaling proteins, which are highly expressed in SHHβ, characterize the G4γ subtype in group 4. These synaptic signaling proteins promote neuronal differentiation marker proteins, including Syn1. Synaptic signaling proteins are downregulated except in the G4γ subtype and significantly associated with good prognosis for patients with medulloblastoma. Similarly, proteasome activity is a regulatory mechanism that cannot be detected through genomic or transcriptomic analyses alone. This highlights the importance of proteogenomic analyses in diagnosis, as they can reveal critical regulatory processes that are overlooked by other methods.

Another important aspect of MS-based proteome analysis is its ability to investigate post-translational modifications. The G4α subtype, known for its poor prognosis, can be characterized by global proteome and phosphoproteome analyses, revealing EMT functions and RTK signaling pathways. By using activity-based phosphoproteomic analysis and kinase–substrate network, we identified subtype-specific kinases and corresponding inhibitors targeting a central kinase within a kinome module. We demonstrated that these selected kinase-specific inhibitors can effectively inhibit medulloblastoma cell growth in a subtype-specific manner. Furthermore, our proteome analyses revealed a dual therapeutic mechanism of Met kinase inhibition. This mechanism not only inhibits EMT, a critical process in tumor progression, but also enhances synaptic signaling recovery, thereby promoting a less aggressive neuronal subtype of medulloblastoma. This highlights the utility of activity-based phosphoproteomic analysis in therapeutic target selection.

Proteome-based prognostic markers and potential therapeutic targets can be strategically paired. For instance, when RNA markers related to cell-cycle functions predict a poor prognosis in patients with SHH medulloblastoma, Cdk1 and Cdk2 inhibitors can be beneficial. Similarly, when EMT function-related protein markers indicate a poor prognosis in patients with group 4 medulloblastoma, Met inhibitors are advantageous. Given that many small-molecule drugs and libraries targeting kinases have been developed — and that most kinases can be targeted with known drugs — our method for identifying prognostic markers and specific kinase inhibitor targets can be highly effective for repositioning known kinase inhibitors.

Through multi-omics analysis, we identified several subtype-specific prognostic marker–therapeutic target combinations. We suggest that multi-omics approaches, including proteomic and phosphoproteomic analyses, will accelerate diagnostics and therapeutics by enhancing the efficiency of drug and marker development.

## Supplementary information

Supplementary Information

## Data Availability

Raw omics data have been deposited in public repositories. Proteomic data have been deposited in the Proteomic Data Commons (PDC, https://proteomic.datacommons.cancer.gov/pdc/edu/; ID PDC000522, PDC000523, PDC000524, and PDC000525). DNA methylation idat files have been deposited to the Gene Expression Omnibus (GEO; ID GSE209668). Raw sequencing (fastq) files have been deposited into the SRA database (https://www.ncbi.nlm.nih.gov/sra) under project ID PRJNA862984, PRJNA863327, PRJNA864070, and PRJNA865394.
