# Supplementary material for: Comprehensive proteogenomic characterization reveals clinically relevant molecular subtypes associated with medulloblastoma progression
Source: Exp Mol Med. 2026 Jun 5;58(6):1885–99. doi: 10.1038/s12276-026-01732-0 (PMC13323399; doi:10.1038/s12276-026-01732-0)

Supplementary methods

Tissue cryopulverization

The frozen tissue samples were weighed and washed with cold phosphate-buffered saline (PBS) to remove contaminating blood, placed into tissueTUBEs (Covaris, Woburn, MA), snap-frozen in liquid nitrogen and pulverized using a cryoPREP Tissue Disruption system (CP02, Covaris). The pulverized tissue powder was aliquoted (10-20 mg) for DNA, RNA and protein extraction.

DNA and RNA extraction and quality analysis

In the case of frozen tissues, genomic DNA was extracted from pulverized tissues using a MagNA Pure 24 Total NA Isolation kit with a MagNAPure 24 automated instrument (Roche, Switzerland) or Allprep DNA/RNA Mini Kit (Qiagen, Germany). In the case of blood samples, genomic DNA was extracted from buffy coat using Maxwell® 16 Blood DNA Purification Kit with Maxwell® 16 automated instrument (Promega, USA) or using Maxwell® RSC Buffy Coat DNA Kit with a Maxwell® RSC 48 automated instrument (Promega, USA) or using a QIAamp DNA Blood Mini kit (Qiagen, Germany). Purified gDNA was analyzed for concentration and purity with a Nanodrop 8000 (Thermo Fisher, USA). The integrity of the gDNA was analyzed with 1% gel electrophoresis. DNA quantitation was analyzed using a Qubit dsDNA BR Assay Kit (Thermo Fisher, USA).

In the case of frozen tissues, total RNA was extracted from pulverized tissues using an Allprep DNA/RNA Mini Kit with a QIAcube automated instrument (Qiagen, Germany) or an RNeasy Mini Kit with a QIAcube automated instrument (Qiagen, Germany). Purified RNA was analyzed for concentration and purity with a Nanodrop 8000 (Thermo Fisher, USA). For RNA quality analysis, RNA was analyzed using an RNA Nano 6000 Kit with a Bioanalyzer 2100 (Agilent, USA).

Whole-exome sequencing

The SureSelect Target Enrichment workflow is a solution-based system that utilizes ultralong 120-mer biotinylated cRNA baits to capture regions of interest, enriching them from an NGS genomic fragment library. To generate standard exome capture libraries, we used the Agilent SureSelectXT Low Input Target Enrichment protocol for the Illumina paired-end sequencing library with 1 μg of input gDNA. The DNA quantity and quality were measured by PicoGreen and agarose gel electrophoresis, respectively. We used 1 μg of each genomic DNA diluted with EB Buffer and sheared to a target peak size of 150–200 bp with the Covaris LE220 focused-ultrasonicator (Covaris, Woburn, MA) according to the manufacturer's recommendations. Fragmentation is followed by end repair and the addition of an 'A' tail. Agilent adapters were then ligated to the fragments. After assessing the ligation efficiency, the adapter-ligated product was PCR amplified. The final purified product was quantified with the TapeStation DNA ScreenTape D1000 platform (Agilent). For exome capture, 250 ng of DNA library was mixed with hybridization buffers, blocking mixes, RNase block and 5 μl of SureSelect all exon capture library, according to the standard Agilent SureSelect Target Enrichment protocol. The captured DNA was washed and amplified. Then, the final purified product was quantified by qPCR according to the qPCR Quantification Protocol Guide (KAPA Library Quantification kits for Illumina Sequencing platforms) and tested for quality with the TapeStation DNA ScreenTape D1000 platform (Agilent). Illumina utilizes a unique amplification reaction that occurs on the surface of the flow cell. A flow cell containing millions of unique clusters is loaded into the Illumina platform for automated cycles of extension and imaging. Sequencing-by-synthesis utilizes four proprietary nucleotides possessing reversible fluorophore and termination properties. Each sequencing cycle occurs in the presence of all four nucleotides, leading to higher accuracy than methods where only one nucleotide is present in the reaction mix at a time. This cycle is repeated one base at a time, generating a series of images, each

representing the extension of a single base at a specific cluster. The Illumina platform generates raw images and performs base calling through its integrated primary analysis software, RTA (Real Time Analysis). The base calling files, which are in binary format, were converted into FASTQ by the Illumina package bcl2fastq v2.20.0. The demultiplexing option (--barcode-mismatches) was set to 0.

Whole-genome sequencing

The samples were prepared according to the Illumina TruSeq Nano DNA library preparation guide. The libraries were sequenced using the Illumina NovaSeq6000 platform. Each sequenced sample was prepared according to the Illumina TruSeq Nano DNA sample preparation guide to obtain a final library with an average insert size of 300-400 bp. One hundred nanograms of genomic DNA was fragmented by the Covaris system, which generates dsDNA fragments with 3' or 5' overhangs. The dsDNA fragments with 3' or 5' overhangs were converted into blunt ends using an end repair mix. The 3' to 5' exonuclease removed the 3' overhangs, and the polymerase filled the 5' overhangs. Following end repair, the appropriate library size was selected using different ratios of the sample purification beads. A single 'A' nucleotide was added to the 3' ends of the blunted fragments to prevent them from ligating to one another during the adapter ligation reaction. A corresponding single 'T' nucleotide on the 3' end of the adapter provides a complementary overhang for the adapter to be ligated to the fragment. Multiple indexing adapters be ligated to the ends of the DNA fragments to prepare them for hybridization onto a flow cell. PCR was used to amplify the enriched DNA library for sequencing. PCR was performed with a PCR primer solution that anneals to the ends of each adapter. Quality control analysis of the sample library and quantification of the DNA library templates was performed by Macrogen. For cluster generation, the library was loaded into a flow cell, where the fragments were captured on a lawn of surface-bound oligos

complementary to the library adapters. Each fragment was then amplified into distinct, clonal clusters through bridge amplification. After cluster generation, the templates were submitted for sequencing. Illumina SBS technology utilizes a proprietary reversible terminator-based method that detects single bases as they are incorporated into DNA template strands. As all 4 reversible, terminator-bound dNTPs are present during each sequencing cycle, natural competition minimizes incorporation bias and greatly reduces the raw error rates relative to those of other technologies. The result is highly accurate base-by-base sequencing that virtually eliminates sequence-context-specific errors, even within repetitive sequence regions and homopolymers. The Illumina Platform generates raw images and performs base calling through an integrated primary analysis software called RTA (Real Time Analysis). The BCL/cBCL (base calls) binary is converted into FASTQ using the Illumina package bcl2fastq2-v2.20.0. The demultiplexing option (--barcode-mismatches) was set to a perfect match (value: 0).

Whole-transcriptome sequencing

The total RNA concentration was calculated by Quant-IT RiboGreen (Invitrogen, #R11490). To assess the integrity of the total RNA, samples were run on the TapeStation RNA ScreenTape platform (Agilent, #5067-5576). Only high-quality RNA preparations with RIN values greater than 7.0 were used for RNA library construction.

A library was independently prepared with 1 μg of total RNA for each sample by an Illumina TruSeq Stranded mRNA Sample Prep Kit (Illumina, Inc., San Diego, CA, USA, #RS-122-2101). The first step in the workflow involves purifying the poly-A-containing mRNA molecules using poly-T-attached magnetic beads. Following purification, the mRNA was fragmented into small pieces using divalent cations under elevated temperature. The cleaved RNA fragments were copied into first-strand cDNA using SuperScript II reverse transcriptase (Invitrogen, #18064014) and random primers. This was followed by second-strand cDNA

synthesis using DNA Polymerase I, RNase H and dUTP. These cDNA fragments were then subjected to an end-repair process, the addition of a single 'A' base, and adapter ligation. The products were then purified and enriched with PCR to create the final cDNA library.

The libraries were quantified using KAPA Library Quantificatoin kits for Illumina Sequecing platforms according to the qPCR Quantification Protocol Guide (KAPA BIOSYSTEMS, #KK4854) and qualified using the TapeStation D1000 ScreenTape platform (Agilent Technologies, # 5067-5582). Indexed libraries were then paired-end (2×100 bp) sequenced with an Illumina NovaSeq (Illumina, Inc., San Diego, CA, USA) at Macrogen Incorporated.

Methylome

DNA samples were checked for quality using a NanoDrop® ND-2000 UV–Vis spectrophotometer. Then, the samples were electrophoresed on agarose gels, and samples with intact genomic DNA showing no smearing on agarose gel electrophoresis were selected for the experiment. Intact genomic DNA was diluted to 50 ng/µl based on Quant-iT Picogreen (Invitrogen) quantitation. All prepared samples were bisulfite-converted according to the Zymo EZ DNA methylation kit protocols.

A total of 600 ng of input gDNA was required for bisulfite conversion. The conversion reagent was added, and the reaction mixture was incubated in a thermocycler for denaturation. CT-converted DNA was washed and desulfonated with desulfonation buffer. After desulfonation, the DNA was washed again and eluted in 12 µl elution buffer.

The whole-genome amplification process requires 250 ng of input bisulfite-converted DNA, MA1, which creates a sufficient quantity of DNA (1000X amplification) to be used on a single BeadChip in the Infinium methylation assay (Illumina RPM and MSM). After amplification, the product was fragmented using a proprietary reagent (FMS), precipitated with 2-propanol

(plus precipitating reagent; PM1), and resuspended in formamide-containing hybridization buffer (RA1). The DNA samples were denatured at 95°C for 20 min and then placed in a humidified container for a minimum of 16 h at 48°C, allowing the CpG loci to hybridize to the 50-mer capture probes.

Following hybridization, the BeadChip/Te-Flow chamber assembly was placed on the temperature-controlled Tecan Flowthrough Chamber Rack, and all subsequent washing, extension, and staining steps were performed by adding the reagents to the Te-Flow chamber.

For the allele-specific single-base extension assay, primers were extended with a polymerase and labeled nucleotide mix (TEM) and stained with repeated application of STM (staining reagent) and ATM (anti-staining reagent). After staining was complete, the slides were washed with low-salt wash buffer (PB1), immediately coated with XC4, and then imaged on the iScan System.

Protein extraction and peptide digestion

Tissue powder samples were solubilized in SDS solubilization buffer (5% SDS, 50 mM TEAB pH 8.5) using an S220 Focused-ultrasonicator (Covaris). Proteins were digested using S-Trap™ spin columns (Protifi, Huntington, NY) according to the manufacturer’s instructions. The samples were reduced by DTT and alkylated by iodoacetamide (IAA). After quenching the alkylation reaction, additional SDS and phosphoric acid were added so that the final concentration was 5% SDS and 1.2% phosphoric acid. The acidified samples were mixed with 90% methanol in 100 mM TEAB, loaded into S-Trap micro columns, and incubated with mass spectrometry-grade trypsin/LysC (Promega) for 3 h at 47°C. The eluted peptides were evaporated using a vacuum concentrator and cleaned up using C18 spin columns (Thermo Fisher Scientific, Rockford, IL).

TMT 11-plex labeling

Desalted peptide samples were reconstituted in 100 mM TEAB and labeled using TMT 11-plex reagents (Thermo Fisher Scientific). Each prepared TMT reagent was transferred to the peptide sample, and the mixture was incubated for 1 h, quenched by the addition of 8 μL of 5% hydroxylamine and then incubated for a further 15 min at room temperature. Differently labeled 11-plex peptides were pooled and dried using a vacuum concentrator.

Peptide fractionation by mid-pH reversed-phase liquid chromatography

The pooled 11-plex TMT-labeled sample was separated using an Agilent 1260 Infinity HPLC system (Agilent, Palo Alto, CA). An Xbridge C18 analytical column (4.6 mm × 250 mm, 130 Å, 5 um) and a guard column (4.6 mm × 20 mm, 130 Å, 5 um) were used for peptide separation. Solvents A and B were 10 mM triethylammonium bicarbonate (TEAB) in water (pH 7.5) and 10 mM TEAB in 90% acetonitrile (ACN, pH 7.5), respectively. Peptide fractionation was performed using a 120 min gradient at a flow rate of 500 μL/min as follows: 0% solvent B for 15 min, 0 to 5% solvent B over 10 min, from 5 to 35% solvent B over 60 min, from 35 to 70% solvent B over 15 min, 70% solvent B for 10 min, and from 70 to 0% solvent B over 10 min. A total of 96 fractions were collected, with one collected every minute from 15 to 110 min, and pooled into 24 noncontinuous peptide fractions (i.e., #1–#25–#49–#73, #2–#26–#50–#74, …, #24–#48–#72–#96) and dried using a concentrator.

Phosphopeptide enrichment using immobilized metal affinity chromatography (IMAC)

Ni-NTA agarose beads (Qiagen, Valencia, CA) were washed with deionized water and treated with 100 mM EDTA, pH 8.0, for 30 min with end-over-end rotation. The EDTA solution was removed, and the beads were washed with deionized water and treated with 10 mM aqueous $FeCl_3$ metal ion solution for 30 min with end-over-end rotation. After the removal of excess

metal ions, the beads were washed with deionized water, resuspended in 1:1:1 acetonitrile/methanol/0.01% acetic acid solution and aliquoted into microcentrifuge tubes. Fractionated peptide samples were resuspended in resuspension buffer (80% acetonitrile, 0.1% TFA). After washing the aliquoted Fe^{3+}-NTA beads with resuspension buffer, the resuspended peptide sample was added and incubated with end-over-end rotation for 30 min. The supernatant was collected and dried for future analysis, the beads were washed with resuspension buffer, and the remaining solution was discarded. The enriched phosphopeptide was eluted with 1:1:1 acetonitrile/2.5% ammonia–water/2 mM phosphate buffer, acidified with 10% TFA solution and dried using a vacuum concentrator.

LC–MS/MS analysis

TMT-labeled peptides prepared for global proteome and phosphoproteome analysis were resuspended with 0.1% formic acid in water, separated using an Ultimate 3000 RSLCnano system (Thermo Fisher Scientific, San Jose, CA, USA) and analyzed using a Q Exactive HF-X hybrid quadrupole-Orbitrap mass spectrometer or a Q-Exactive plus hybrid quadrupole Orbitrap mass spectrometer (Thermo Fisher Scientific, San Jose, CA, USA).

For the Q Exactive HF-X hybrid quadrupole-Orbitrap mass spectrometer, solvents A and B were 0.1% FA in water and 0.1% FA in acetonitrile, respectively. The peptides were loaded onto the trap column (Acclaum PepMapTM 100, 75 μm x 2 cm), separated by the analytical column (EASY-Spray column, PepMap^{TM} RSLC C18, 75 μm x 50 cm, Thermo Fisher Scientific) with a gradient of 5 to 24% solvent B for 150 min and 24 to 36% solvent B for 30 min (for the global proteome) or 5 to 24% solvent B for 170 min and 24 to 36% solvent B for 10 min (for the phosphoproteome) at a flow rate 0.3 μL/min. The Q Exactive HF-X Orbitrap mass analyzer was operated in a top 10 data-dependent method. Full MS scans were acquired

over the range m/z 350–2000 with a mass resolution of 120,000 (at m/z 200). The AGC target value was 3.00E+06. The ten most intense peaks with a charge state ≥2 were fragmented in the higher-energy collisional dissociation (HCD) collision cell with a normalized collision energy of 32, and tandem mass spectra were acquired in the Orbitrap mass analyzer with a mass resolution of 45,000 at m/z 200.

For a Q-exactive plus hybrid quadrupole Orbitrap mass spectrometer, peptides were loaded from an RS autosampler and separated with a linear gradient of ACN/water containing 0.1% formic acid with a flow rate of 300 nl/min. Chromatographic separation of peptides was achieved using the Ultimate 3000 RSLC nano system equipped with the same column setting with HF-X. The LC eluent was electrosprayed directly from the analytical column, and a voltage of 2.0 kV was applied via the liquid junction of the nanospray source. Peptide mixtures were separated with a stepwise gradient from 7% to 60% ACN over 105 min (7% to 25% min for 85 min & 25% to 60 min for 20 min). The analysis method consisted of a full MS scan with a range of 350–2000 m/z and data-dependent MS/MS (MS2) on the ten most intense ions from the full MS scan. The mass spectrometer was programmed to acquire data in data-dependent mode. The mass spectrometer was calibrated with the proposed calibration solution according to the manufacturer's instructions.

Genomic data analysis

Sequencing reads were aligned to the GRCh37/hg19 human reference genome using the Burrows–Wheeler Aligner (BWA) and further processed by GATK to remove low mapping quality reads and realign sequences around indels. To confirm that tumor and blood samples were derived from the same patient, we performed fingerprint analysis using NGSCheckMate, a model-based method that evaluates the correlation of the variant allele fractions at known SNP sites. Somatic SNVs and indels were identified by integrating the results from 3 variant

calling algorithms: TNhaplotyper, TNscope and TNsnv. For tumor samples without matched blood DNA, the somatic status of called mutations was assessed using a virtual normal panel from a set of 433 public samples from healthy, unrelated individuals sequenced to high depth in the context of the 1000 Genomes Project. Putative false positive calls were removed by applying a previously described in-house filtering pipeline. The mutation profiles of the longitudinal medulloblastoma pairs (10 primary and 10 recurrent matched samples) were built by combining the genetic variants identified in the two biopsies from each single patient, each of which was annotated as shared (occurring in both primary and recurrent samples) and primary or recurrent private (occurring exclusively in the primary or in the recurrent sample, respectively). To validate the sharing profile of mutations, the nucleotide at each mutant position was re-called from the raw sequences within both primary and recurrent samples from a single patient. Using this iterative approach, false negative calls were retrieved by identifying mutant reads at genetic positions that had been mis-called as wild type.

Somatic variants were annotated using AnnoVar, which aggregates information from genomic and protein resources in cancer and noncancer variant databases. Variants reported in the noncancer databases with a minor allele frequency ≥ 0.05 were classified as germline polymorphisms and excluded.

The functional effects of missense SNVs and in-frame indels were determined by an ensemble of multiple algorithms. Variants predicted as damaging by two or more algorithms were classified as pathogenic mutations.

Somatic copy number was estimated from WES and WGS by CNVkit. GISTIC2 analysis was then applied to integrate the results from individual patients and identify genomic regions that were recurrently amplified or deleted in the medulloblastoma cohort.

Transcriptomic data analysis

Raw fastq files were processed for adapter trimming using the Cutadapt program (ver. 2.9, https://cutadapt.readthedocs.io/en/stable/index.html). The processed files were aligned to the human reference genome (GRCh38) using the HISAT2 program (ver. 2.1.0, https://github.com/DaehwanKimLab/hisat2). The aligned sam files were converted to bam files and sorted by coordinate using the Samtools program (ver. 1.10, http://www.htslib.org). Duplicate reads were removed using Picard (ver. 2.22.1, https://github.com/broadinstitute/picard). The read counts for gene expression were computed using the HTseq program (ver. 0.11.3, https://htseq.readthedocs.io/en/master/). The gene expression values were calculated based on the fragments per kilobase of exon per million (FPKM) value. Genes that had greater than 30% missing values were discarded. The expression levels of the filtered genes were globally normalized with the Quantile normalization method using the R (ver. 4.0) limma package or MATLAB Bioinformatics Toolbox (ver. R2021a).

Methylomic data analysis

For scanning the Illumina 850k EPIC microarray, we used the iScan System, which is a two-color (532 nm/658 nm) confocal fluorescent scanner with 0.54 µm pixel resolution. The scanner excites the fluorophores generated during signal amplification/staining of the allele-specific (one-color) extension products on the BeadChips. The image intensities were extracted using Illumina's GenomeStudio software.

Proteomic data analysis

Raw files of tandem mass spectra were converted into mzML files using the msConvert program (ver. 3.0, https://bio.tools/msconvert). The mzML spectral data were mapped to the

human UniProt database (UP000005640.fas, https://www.uniprot.org/) and quantified using the FragPipe pipeline (ver. 14.0), including MSFragger, Philosopher and TMT-integrator program (ver. 3.1.1, https://msfragger.nesvilab.org). All identified proteins had an FDR of ≤1%, which was calculated at the peptide level. For the global proteome, the search parameters allowed for tryptic specificity of up to two missed cleavages, with methylthio-modifications of cysteine as a fixed modification and oxidation of methionine as a dynamic modification. The mass search parameters for -1, 0, +1, +2, and +3 ions included mass error tolerances of 20 ppm for precursor ions. For the phosphoproteome, the search parameters allowed for tryptic specificity of up to two missed cleavages, with modified serine, tyrosine, and threonine as variable modifications. The mass search parameters for 0, +1, +2, and +3 ions included mass error tolerances of 20 ppm for precursor ions. The protein expression was quantified with the isobaric TMT 11 option and normalized to the relative expression ratio against the global reference pool of each TMT 11 set. Protein sequences with greater than 30% missing values were discarded. The expression ratios of filtered proteins were globally normalized with the Quantile normalization method using the R (ver. 4.0) limma package or MATLAB Bioinformatics Toolbox (ver. R2021a).

Unsupervised clustering

For single-platform data clustering, we used two unsupervised clustering methods, hierarchical clustering and nonnegative matrix factorization (NMF) clustering. Hierarchical clustering was performed using the MEV program (ver. 4.9, https://sourceforge.net/projects/mev-tm4/). NMF clustering was performed using the MATLAB Bioinformatics Toolbox (ver. R2021a). The mean absolute deviation (MAD) of each gene was calculated, and the genes with the top 10%, 20%, and 30% MADs were selected as the core genes. By changing the MAD cutoff and the number of clusters (k-value = 2~8), the cophenetic correlation coefficient and silhouette

coefficient were calculated, and the clustering was optimized based on the determined coefficients.

For multiomic clustering, we constructed a binary matrix of the single-platform cluster to which each sample belonged (Supplementary Fig. 3). With the binary matrix, hierarchical clustering was performed using the MEV program. The alluvial plot was generated with the ggplot2 (ver. 3.3.5) package in R.

Selection of differential expression and functional annotation

For the transcriptomic and global proteomic data, differentially expressed genes and proteins were selected by Gene Set Enrichment Analysis (GSEA) (ver. 4.1.0, https://www.gsea-msigdb.org/gsea/index.jsp). The functions of core genes and proteins were annotated using GSEA and DAVID (https://david.ncifcrf.gov/).

For the phosphoproteomic data, the selection of differentially expressed phosphoproteins and quantitation of kinase activity were performed using the Kinase–Substrate Enrichment Analysis (KSEA) program (https://casecpb.shinyapps.io/ksea/). The kinase–substate network was generated using the Cytoscape program (ver. 3.8.0, https://cytoscape.org).

The heatmaps were drawn using Excel (Microsoft) and MEV. The density plots were drawn using the ggplot2 package in the R program.

Pathway analysis

Core pathways, genes, and proteins were selected from the Reactome (https://reactome.org/) and KEGG (https://www.genome.jp/kegg/) pathways in MSigDB (https://www.gsea-msigdb.org/gsea/msigdb). The pathways and multiome heatmaps were manually drawn using Excel and PowerPoint (Microsoft).

Integrative clustering analysis of medulloblastoma

Integrative clustering analysis of medulloblastoma using transcriptomic, proteomic, and DNA methylation datasets was done. Each omics layer was first pre-processed. To integrate the multi-omics profiles, the Similarity Network Fusion and Consensus Clustering (SNF-CC) was applied.Briefly, sample similarity networks were constructed independently for each omics modality based on pairwise Euclidean distances of selected features. These networks were then fused into a single unified similarity graph using the SNF algorithm, which iteratively integrates complementary information across omics layers while preserving shared structure. The fused similarity matrix was subsequently used for consensus clustering.

Comparison between our own classification (KNCC) and WHO classification

To classify the KNCC medulloblastoma cohort according to the current WHO classification, publicly available Cavalli et al. and Capper et al. DNA methylation reference datasets was utilized. β-values from KNCC tumor samples were integrated with the reference methylation data, followed by dimensionality reduction using t-SNE to visualize sample distribution across medulloblastoma subgroups. To further illustrate the concordance between KNCC tumors and reference-derived subgroups, alluvial plot was generated by mapping Cavalli/Capper subgroup assignments to WHO consensus subtypes, highlighting the classification flow of KNCC samples.

Characterization of medulloblastoma tumors within a developmental framework

To characterize KNCC medulloblastoma tumors within a developmental and molecular framework, KNCC transcriptomic profiles was integrated with the Aldinger et al. reference dataset comprising 366 marker genes representing major cerebellar lineages and

medulloblastoma subgroups. Expression values from KNCC tumors were projected onto the Aldinger diffusion map.Reference samples annotated as RL, GCP, GN, and eCN/UBC together with molecular subgroups (WNT, SHHα/β/γ, Group 3/4 I–VIII), were used to define developmental and WHO molecular contexts.

To investigate the developmental lineage contributions to KNCC medulloblastoma tumors, CIBERSORTx was applied for deconvolution. Aldinger et al. dataset was used as reference signature matrix, including rhombic lip (RL), granule cell precursors (GCP), granule neurons (GN), and excitatory cerebellar nucleus/unipolar brush cells (eCN/UBC). KNCC RNA-seq expression data were normalized and input into CIBERSORTx. The analysis estimated the relative proportions of developmental cell types for each tumor sample. The resulting cell-type fractions were aligned with molecular subgroup classifications (WNT, SHHα/β, Group 3, Group 4a–g).

Identification of transcription factors in KNCC medulloblastoma tumors

To identify the transcription factors in KNCC medulloblastoma tumors SCENIC R was done. Briefly, co-expression modules were first identified using GENIE3/GRNBoost2. Motif enrichment analysis was then performed to link transcription factors to their direct target genes, thereby defining regulons. Finally, regulon activity scores were calculated for each tumor using the AUCell algorithm, which quantifies the enrichment of regulon target genes within individual expression profiles. The resulting regulon activity matrix was visualized as a heatmap across medulloblastoma subtypes.

Comparison of transcriptomic variability between medulloblastoma and glioblastoma

To compare the transcriptomic variability between medulloblastoma (MB) and glioblastoma (GBM), differential expression matrices for both tumor types was used as a input. Only genes

shared across both datasets were retained for analysis. For each gene, the standard deviation (SD) of expression values was calculated separately in MB and GBM cohorts. Additionally, Levene's test was applied across tissues to estimate variance differences, and the F-statistic was recorded as a measure of dispersion heterogeneity. To visualize the distribution of gene expression variability, radial scatter plot was generated using ggplot2. Each point corresponds to a gene, with its radial distance representing the standard deviation of expression within each tumor type. The left semicircle displays MB variability while the right semicircle represents GBM variability.

Tissue microarray and immunohistochemistry

We reviewed hematoxylin–eosin slides and selected three representative areas per slide, often with high mitotic counts, while avoiding necrotic and hemorrhagic regions. Tissue cores (diameter, 2 mm) obtained from these areas were then inserted into recipient TMA blocks (SuperBioChips, Seoul, Republic of Korea).

IHC staining was performed on 2-μm-thick formalin-fixed and paraffin-embedded (FFPE) tissue microarray slides using an automated immunostaining system (BenchMark GX system; Ventana-Roche, Mannheim, Germany). The primary antibodies used in this study are summarized in Supplementary Table XX. For the positive control, internal positive control tissue or cells were used, and for the negative control, primary antibodies were omitted.

Proliferation assay

Cells were plated at a density of $3X10^3$ cells/well in 96-well plates containing DMEM (10% FBS, 1x P/S) for in vitro proliferation assays. The luminescence of viable cells was detected 2 days after plating using a CellTiter-Glo Luminescent Cell Viability Assay Kit according to the manufacturer's protocol (Promega). The luminescence signal was detected by a SpectraMax L

Microplate Reader (Molecular Device) according to the manufacturer’s protocol.

Supplementary figure legends

Supplementary Fig. 1. Status of medulloblastoma multiomic data.

Supplementary Fig. 2. Unsupervised clustering with single-platform data. a. Hierarchical clustering. b. NMF clustering. (Methylome, Transcriptome, Proteome)

Supplementary Fig. 3. Determination of medulloblastoma subtypes using multiomic cluster integration and comparison with SNF cluster

Supplementary Fig. 4. Comparison of subtypes between our own data and other previous researcher's data (Archer et al.,2018).

Supplementary Fig. 5. Comparison of subtypes between our own data and WHO subtype.

Supplementary Fig. 6. Expression patterns of known and newly selected subtype-specific markers of primary medulloblastoma (Leal, Evangelista and Paula et al., 2018).

Supplementary Fig. 7. Proteome functional terms enriched in each subtype.

Supplementary Fig. 8. Visualization of protein function and expression patterns for each medulloblastoma subtype using a protein-protein correlation network in other public dataset (Archer et al.,2018).

Supplementary Fig. 9. Mapping of medulloblastoma WHO subtypes on scRNA-seq data of normal cerebellar cellular differentiation using a diffusion map.

Supplementary Fig. 10. Proportion of neuronal cell types within medulloblastoma tissues (scRNA-seq from Michael Taylor's group). The bar chart visualizes the composition of neuronal cell lineages (RL, GCP, eCN/UBC, GN) across different medulloblastoma subtypes.

Supplementary Fig. 11. Proportion of neuronal cell type within each medulloblastoma patient.

The bar chart visualizes the composition of neuronal cell lineages (RL, GCP, eCN/UBC, GN) across different medulloblastoma subtypes

Supplementary Fig. 12. Expression patterns of representative neuronal differentiation markers on Diffusion map (scRNA-seq, Smith et al., 2022).

Supplementary Fig. 13. NEUROD1 function in medulloblastoma. a. Enrichment of NEUROD1 target gene expression in medulloblastoma. b. Higher correlation of neuronal proteins between RNA and proteins. c. RNA-Protein correlation of representative neuronal differentiation markers. d. Association of NEUROD1 expression with PFS.

Supplementary Fig. 14. Distribution of Pearson's correlation coefficients: between protein and RNA expression in functional groups (left, bottom) and between DNA methylation and RNA expression in functional groups (right)

Supplementary Fig. 15. The expression of representative protein markers by subtype.

Supplementary Fig. 16. Results of tissue microarray analysis (TMA).

Supplementary Fig. 17. Phosphoproteome analysis. a. Selection of phosphoproteins. b. Correaltion between representative kinase and substrate.

Supplementary Fig. 18. Grouping of patients with good prognosis. a. PCA analysis. b. survival analysis grouped by neuronal subtypes c. survival analysis grouped by synaptic signaling protein expression.

Supplementary Fig. 19. Different kinase activity among Group 4 subtypes (KSEA kinase z-score) and Kinase-substrate network enriched in Group 4 subtypes

Supplementary Fig. 20. Comparison of genome between primary and recurrent medulloblastoma

Supplementary Fig. 21. Similarity of primary and recurrent tumor pairs. Result of unsupersived clustering of primary and recurrent medulloblastoma samples (RNA-seq)

Supplementary Fig. 22. Cell to cell correlation network of primary and recurrent medulloblastoma samples (RNA-seq)

Supplementary Fig. 23. Recurrence-related expression pattern analysis (k-means clustering).

Supplementary Fig. 24. Changes (GSEA enrichment score) in enriched protein functions from primary to recurrent medulloblastoma.

Supplementary Fig. 25. Representative protein expression by recurrence

Supplementary Fig. 26. Cell to cell correlation network of medulloblastoma tissues and cell lines

Supplementary Fig. 27. Distribution of functional core protein expression (x-axis: change by recurrence, y-axis: change by Foretinib treatment)

Supplementary Fig. 28. Comparison of the average gene expression differences between primary and recurrent tumor samples in medulloblastoma and glioblastoma (GBM)

Supplementary Fig. 29. Summary of medulloblastoma progression. This figure underscores the diverse cellular origins of medulloblastoma subtypes, detailing their specific differentiation pathways and regulatory mechanisms, which are crucial for targeted therapeutic approaches. (SVZ: subventricular zone, VZ: ventricular zone, GCP: granule cell progenitor, UBC: unipolar brush cell, EGL: external granule layer)

Park et al. Supplementary Fig. 1

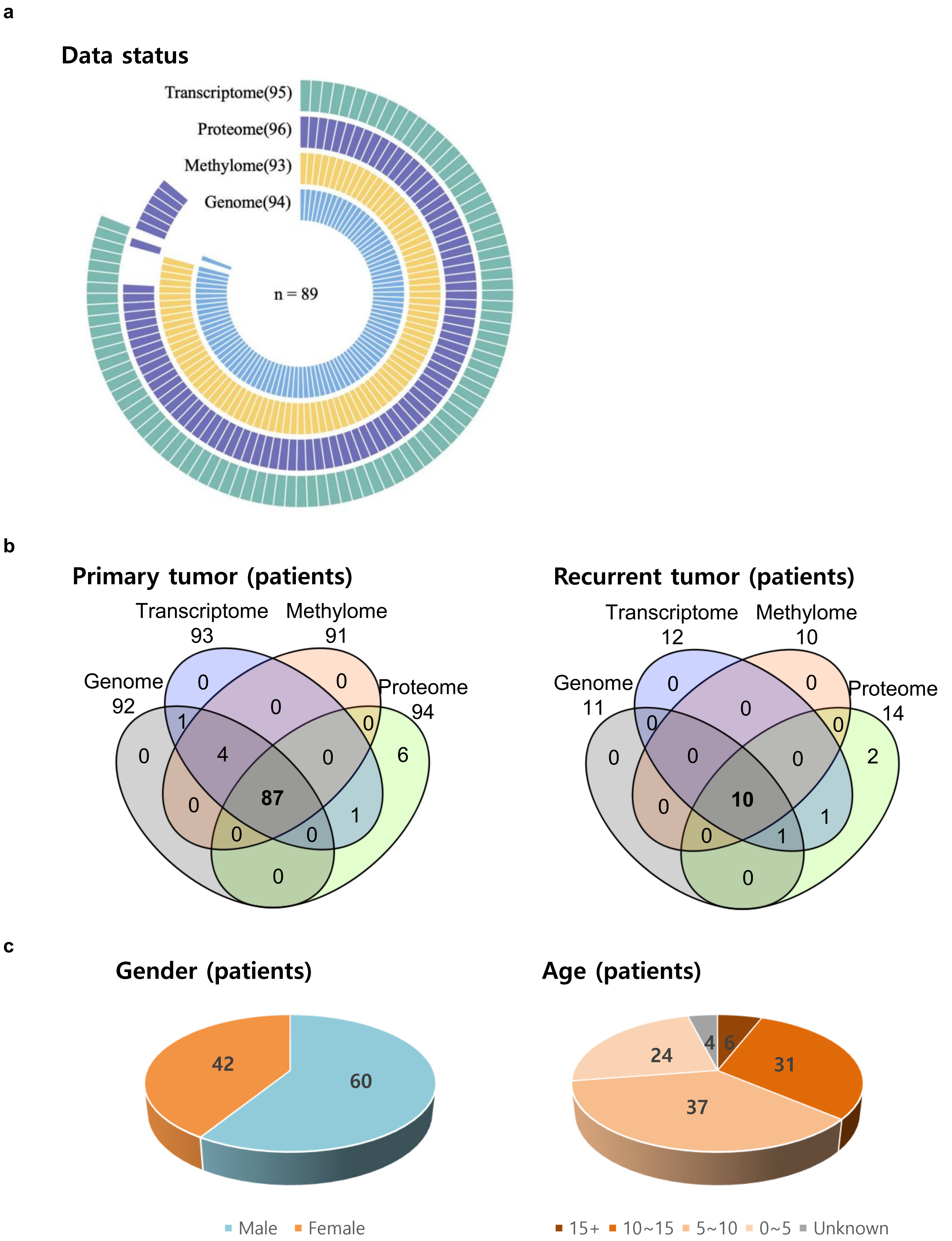

a

Unsupervised hierarchical clustering

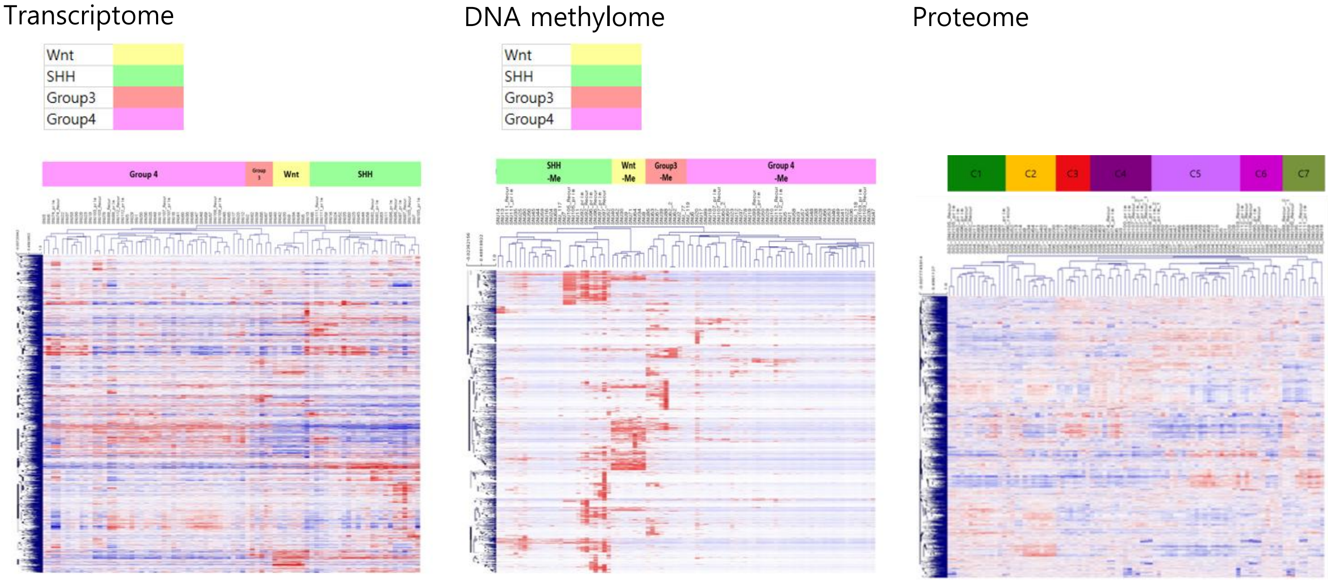

b

NMF clustering

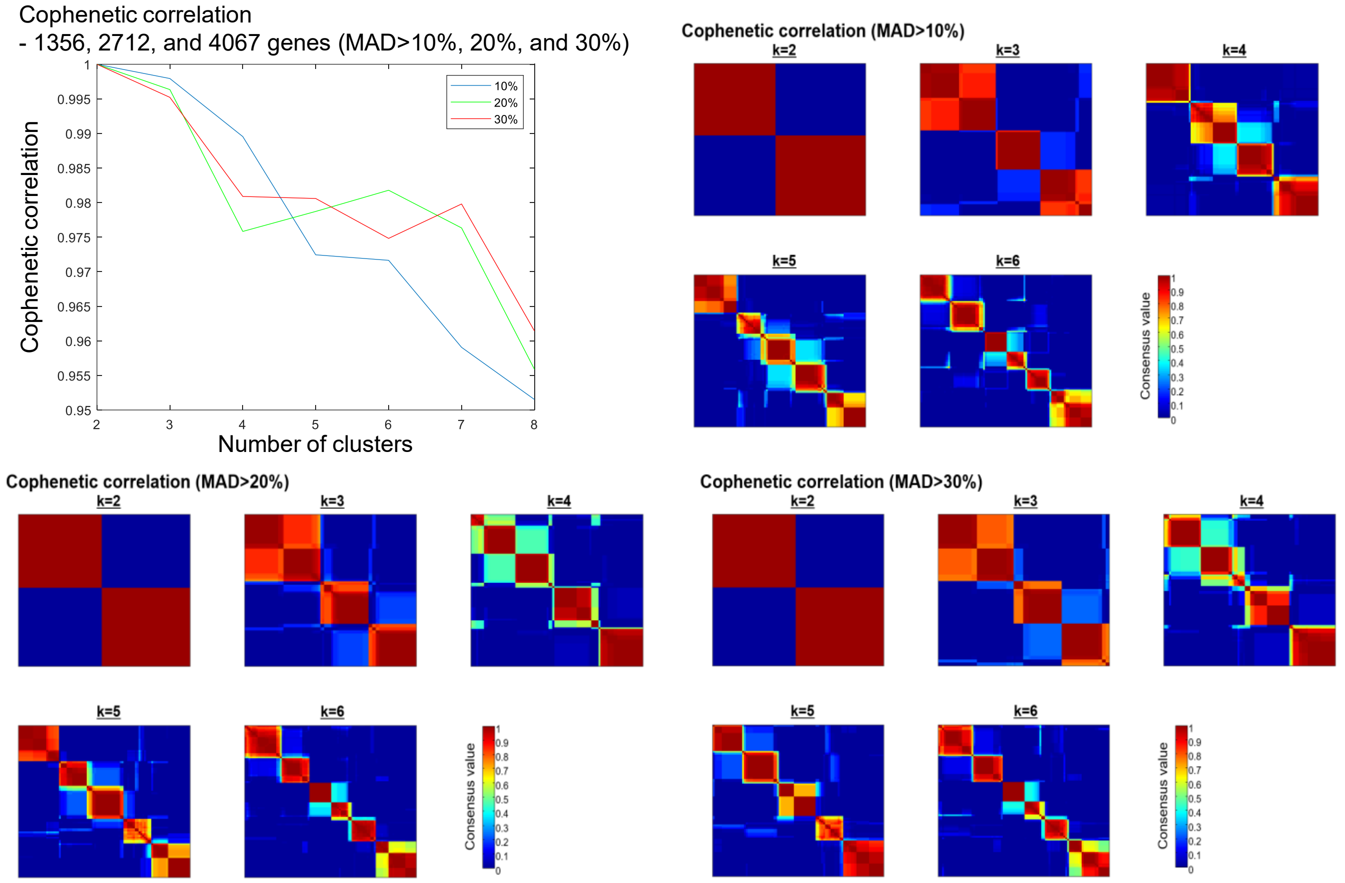

Park et al. Supplementary Fig. 3

Subtyping with multiome analysis

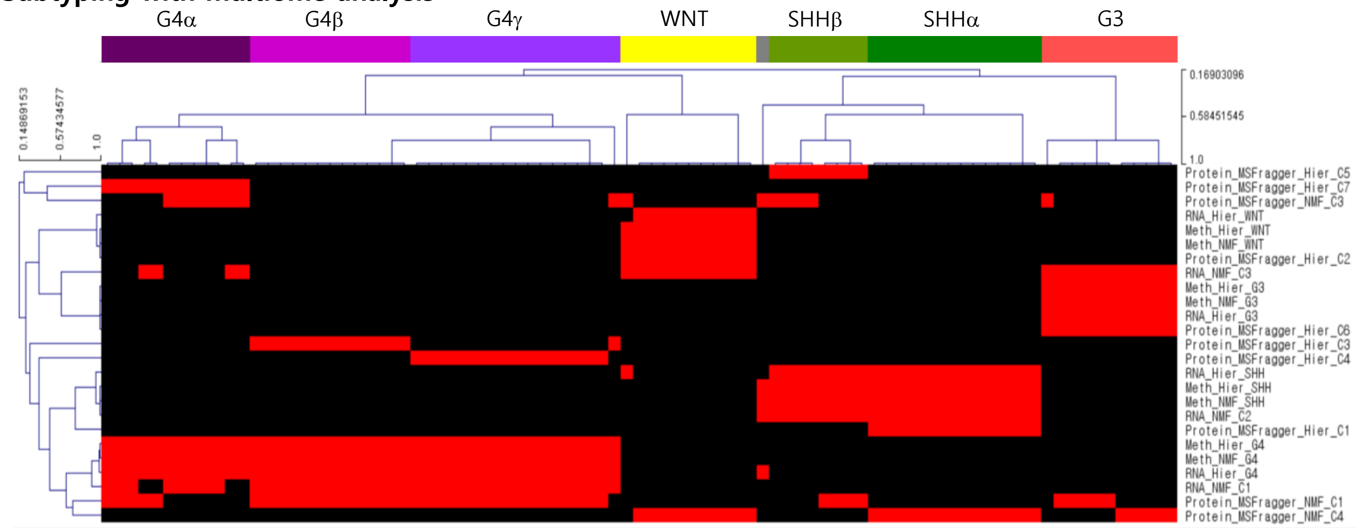

Sample migration by single platform clusters

Hierarchical clustering

NMF clustering

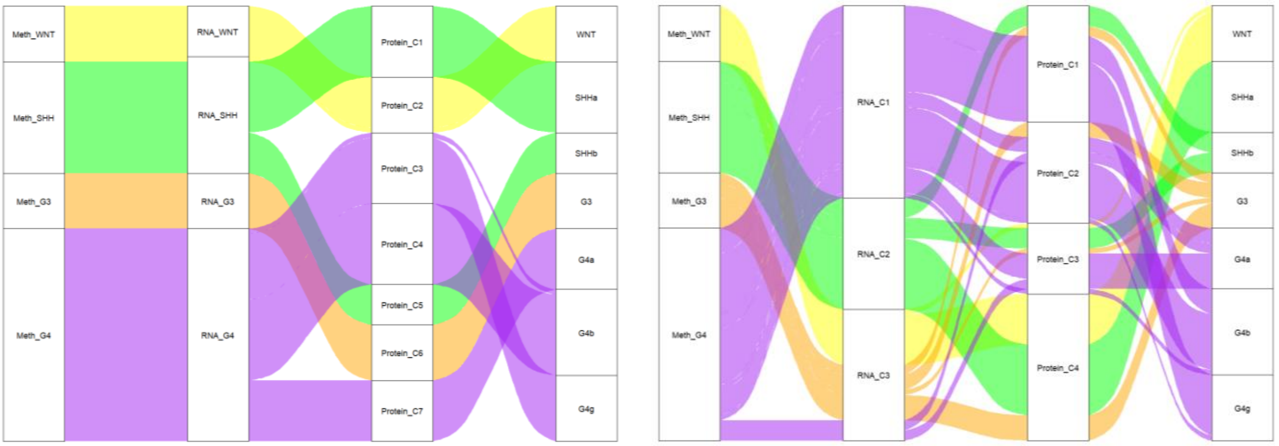

Comparison between Multiome clustering and SNF clustering

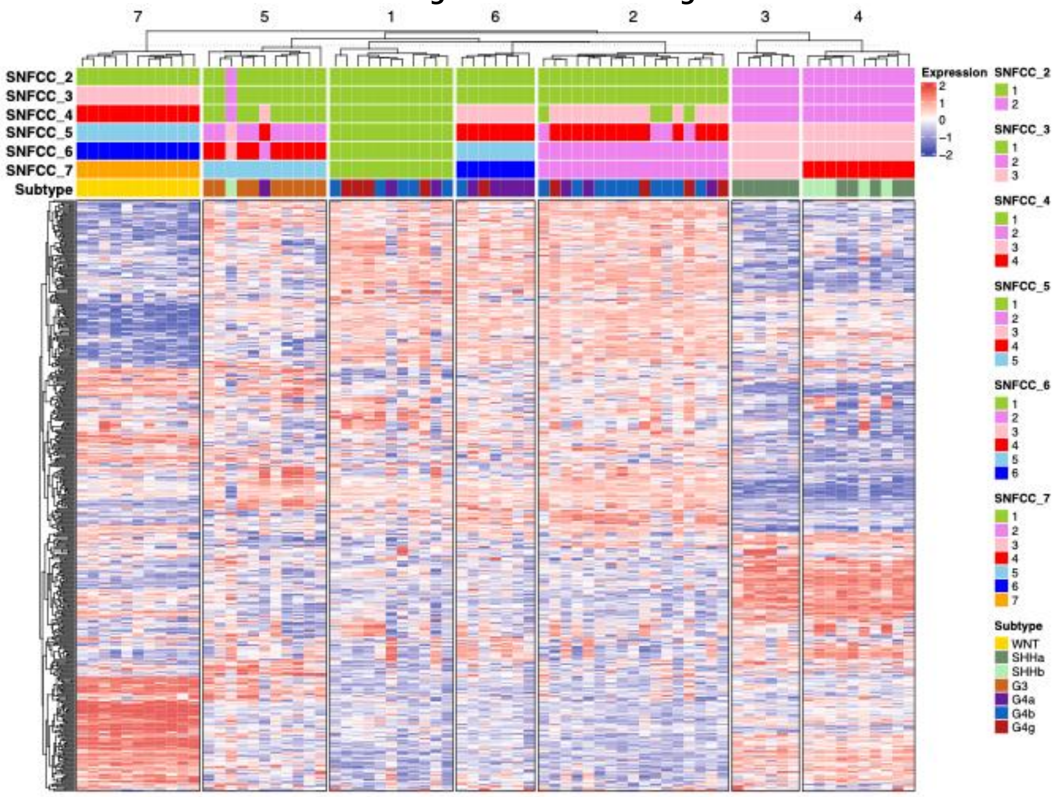

Correlation between subtypes (our own data vs. public dataset) (Archer et al.,2018)

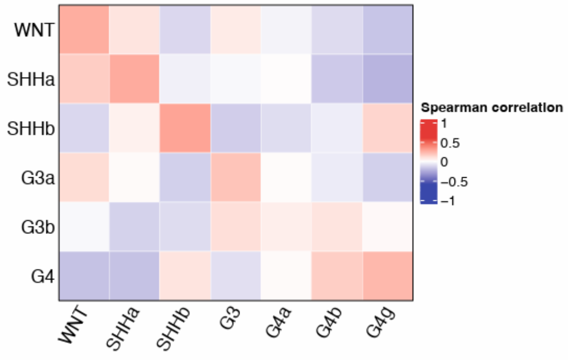

phosphoproteome

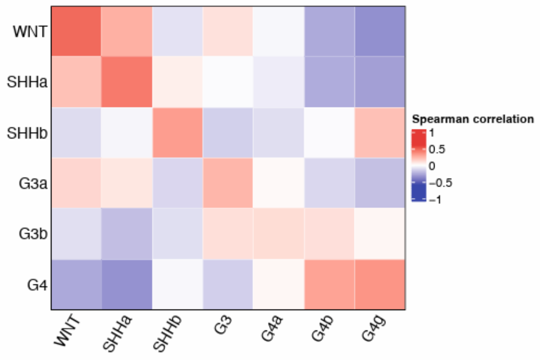

proteome

Sample migration from 12 subtypes by DNA methylation (Cavalli et al. (2017)) to our own 7 subtypes

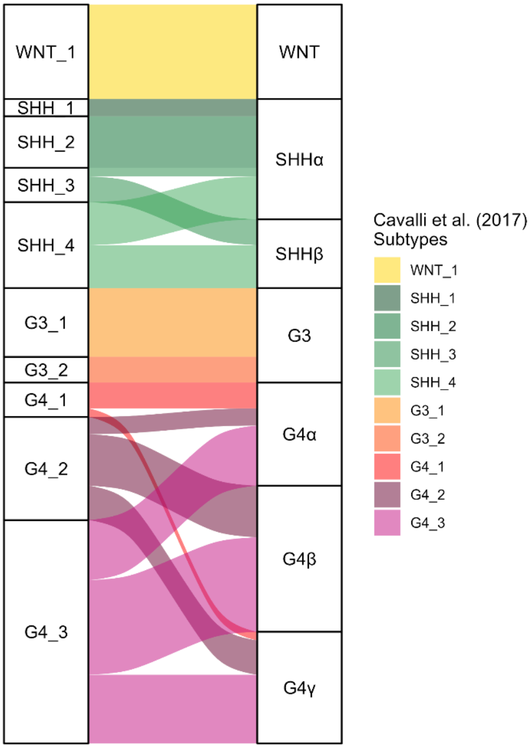

DNA methylation pattern of 1.1k markers (cg probes without SNP) from Cavalli et al. (2017)

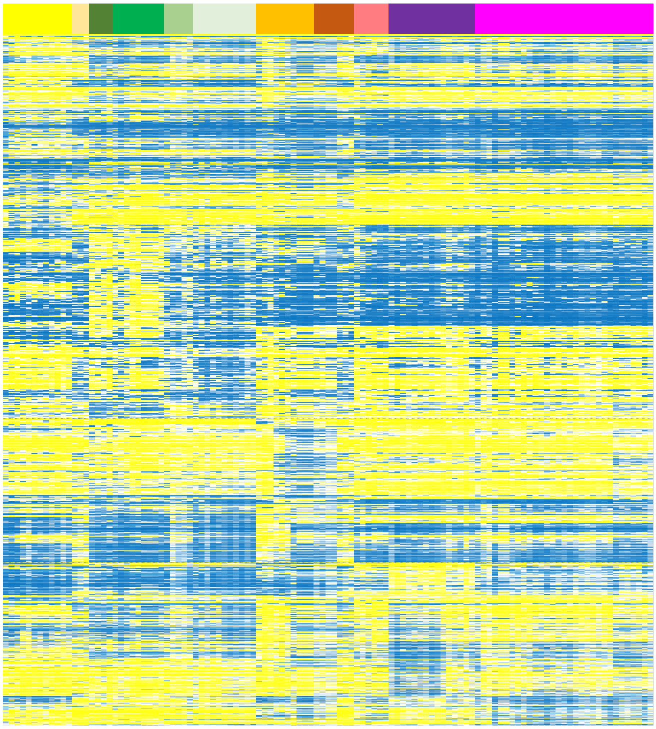

Park et al. Supplementary Fig. 5

Sample migration from 12 subtypes by DNA methylation (WHO classficaiton) to our own 7 subtypes

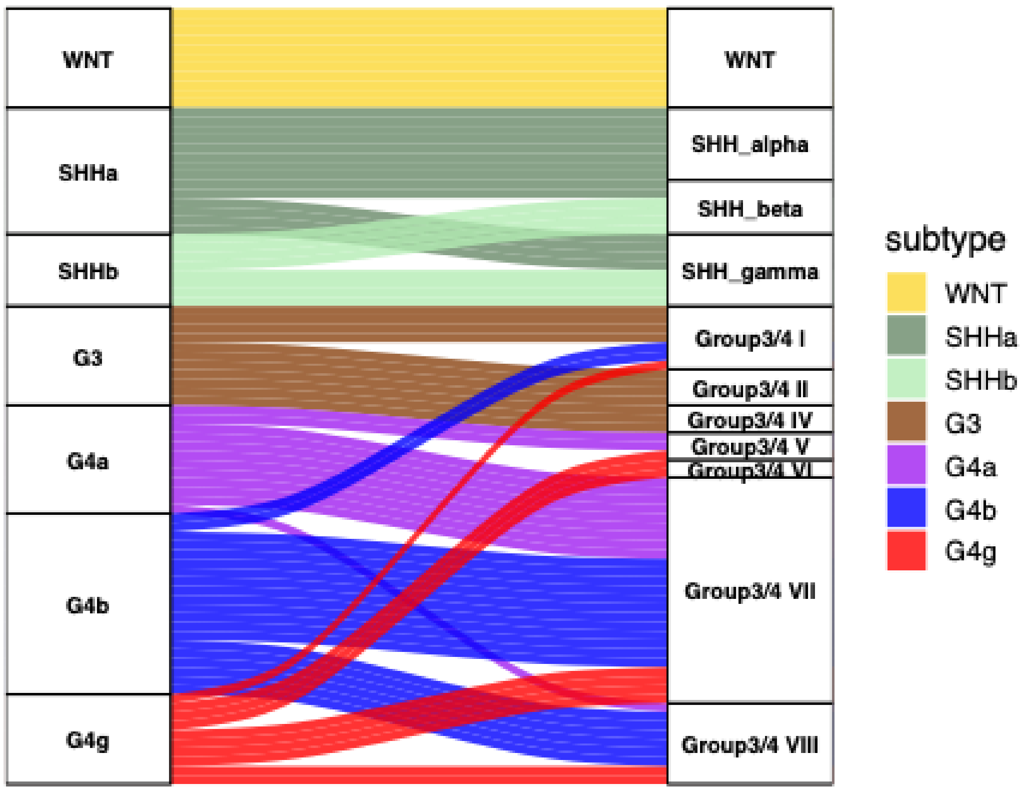

Mapping to our own 7 subtypes on WHO 12 subtypes

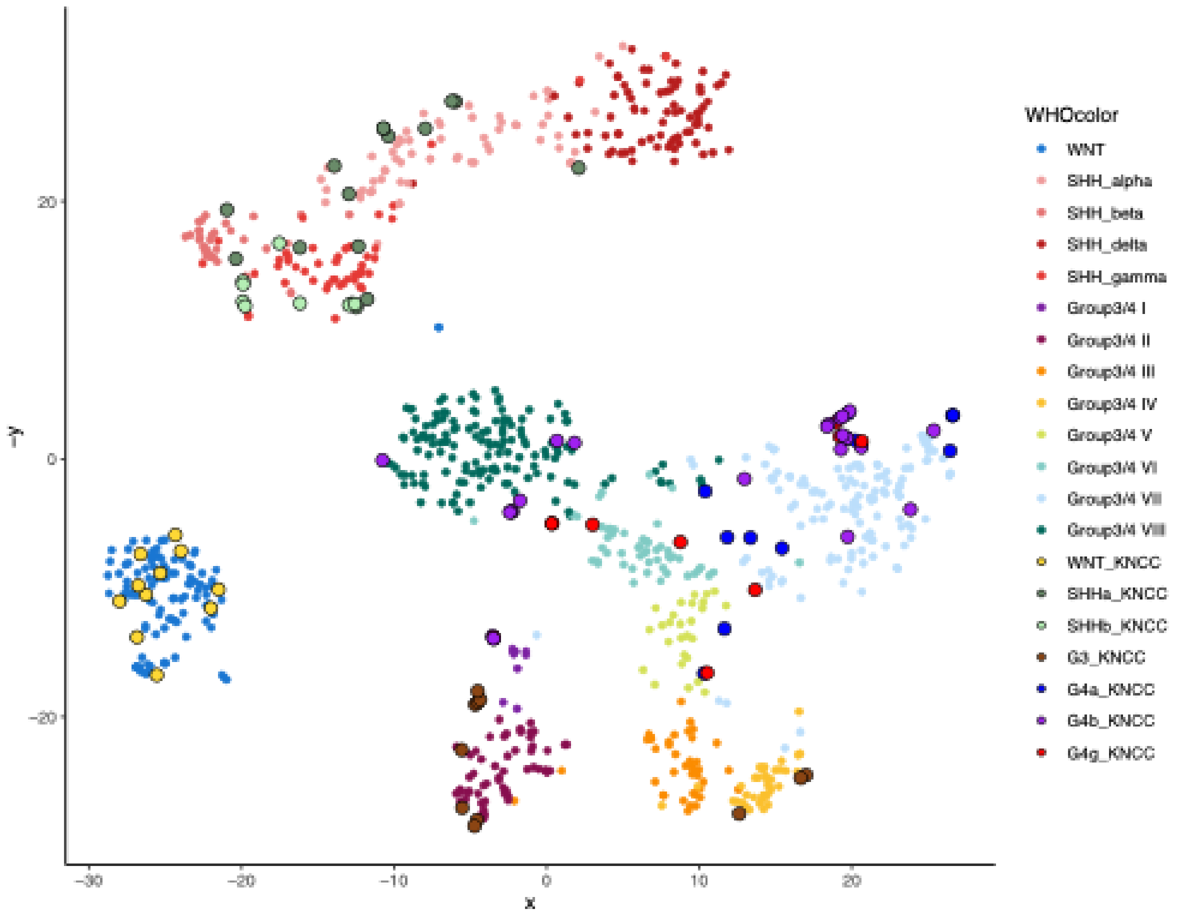

Park et al. Supplementary Fig. 6

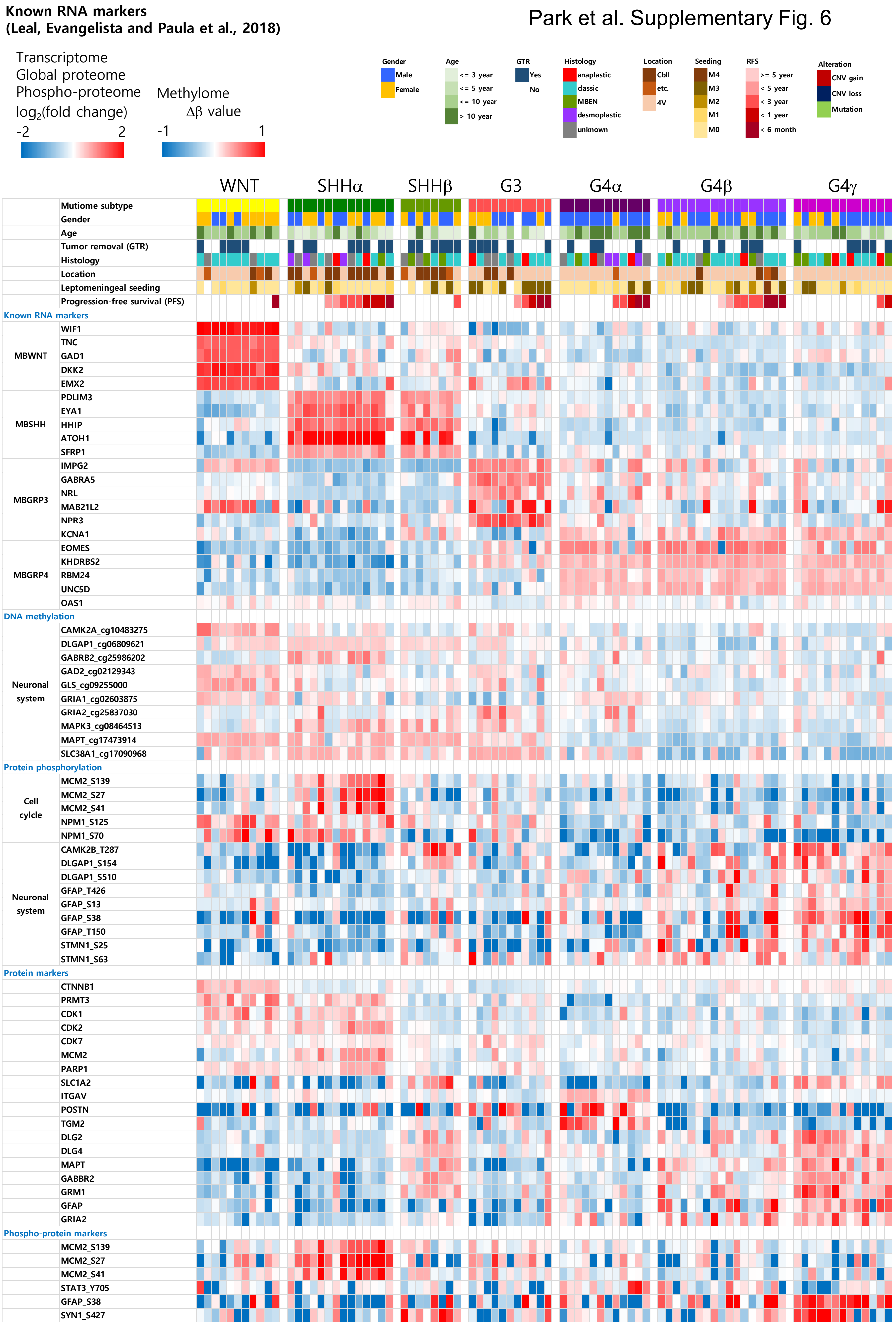

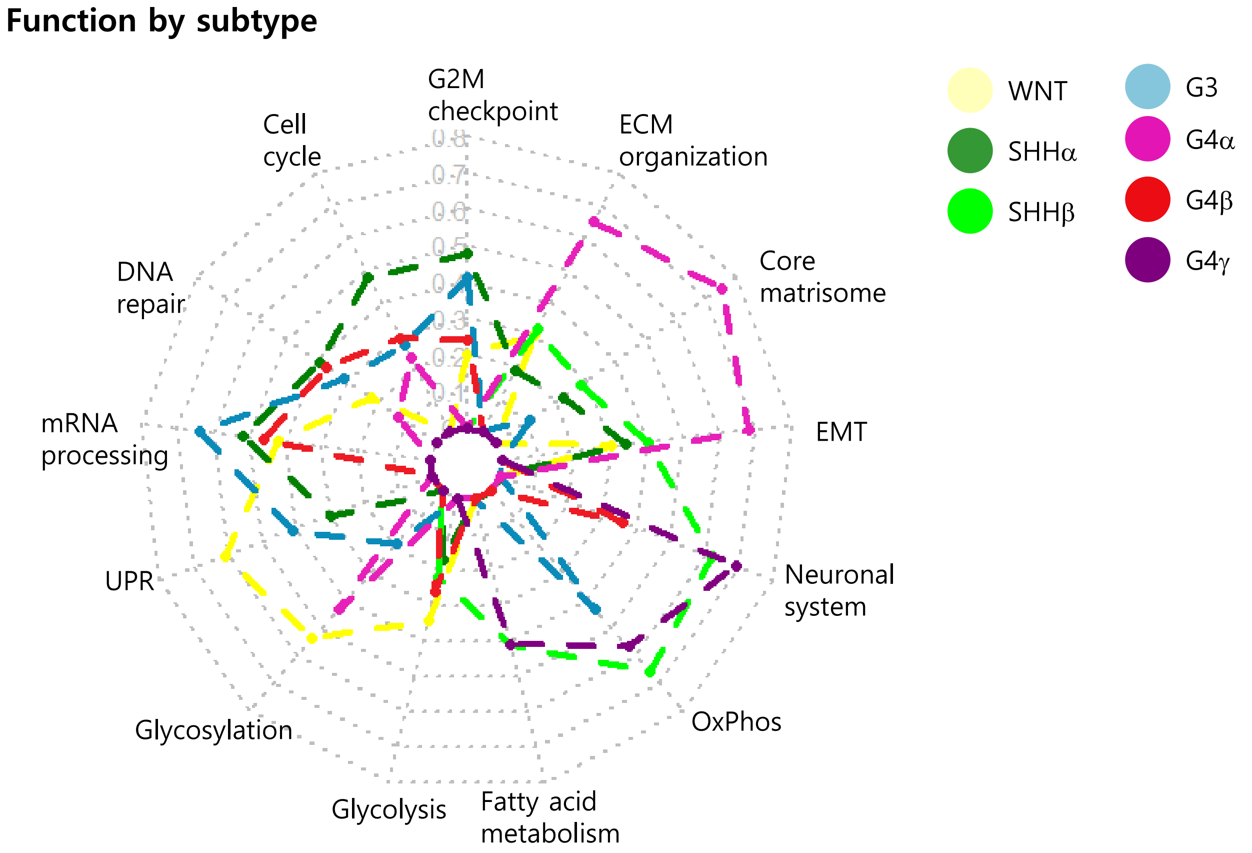
Function by subtype
G2M checkpoint
ECM organization
Core matrisome
EMT
Neuronal system
OxPhos
Fatty acid metabolism
Glycolysis
Glycosylation
UPR
mRNA processing
DNA repair
Cell cycle
0.8
0.7
0.6
0.5
0.4
0.3
0.2
0.1
WNT
SHHα
SHHβ
G3
G4α
G4β
G4γ

Correlation network and subtype-specific expression in public dataset (Archer et al.,2018)

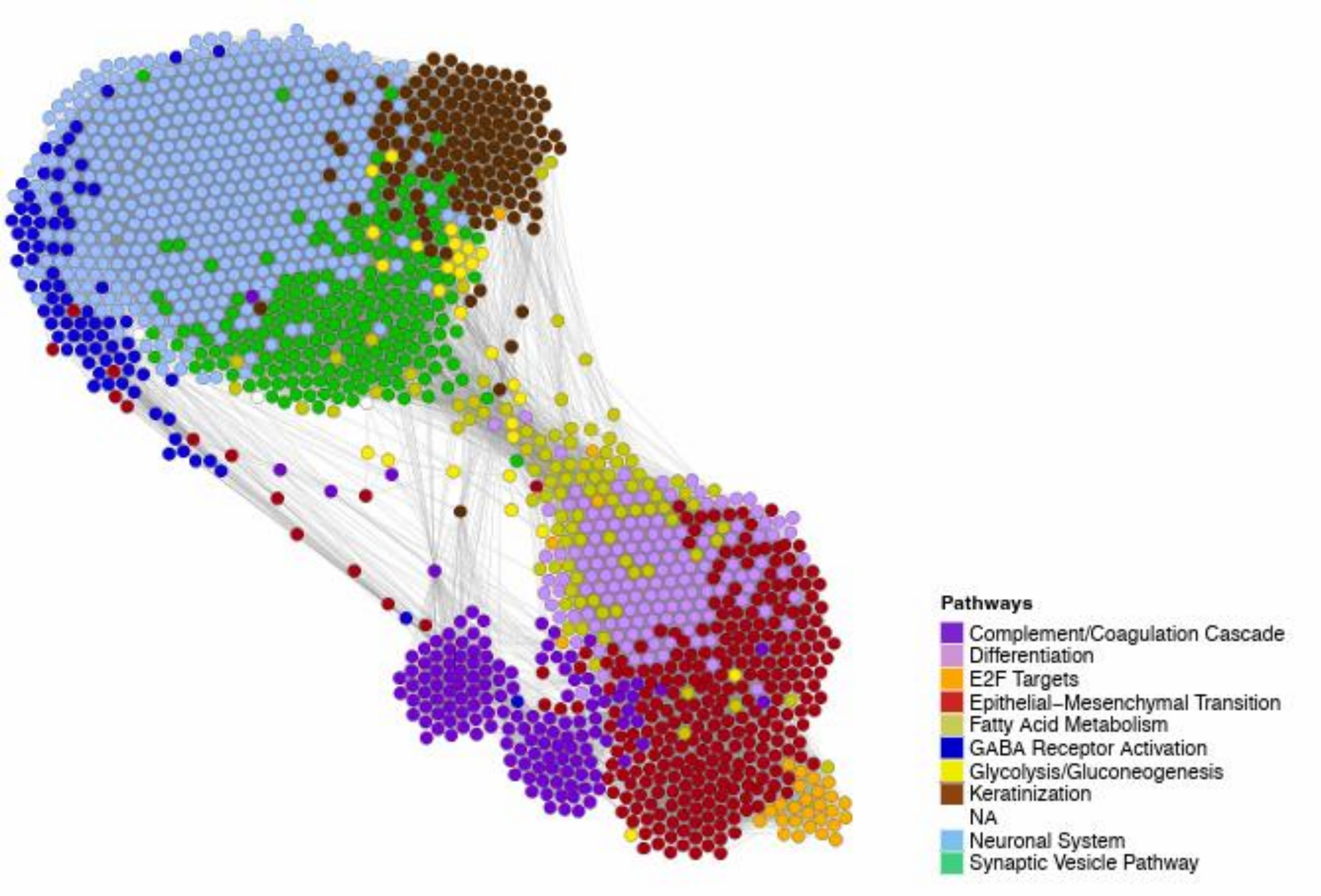

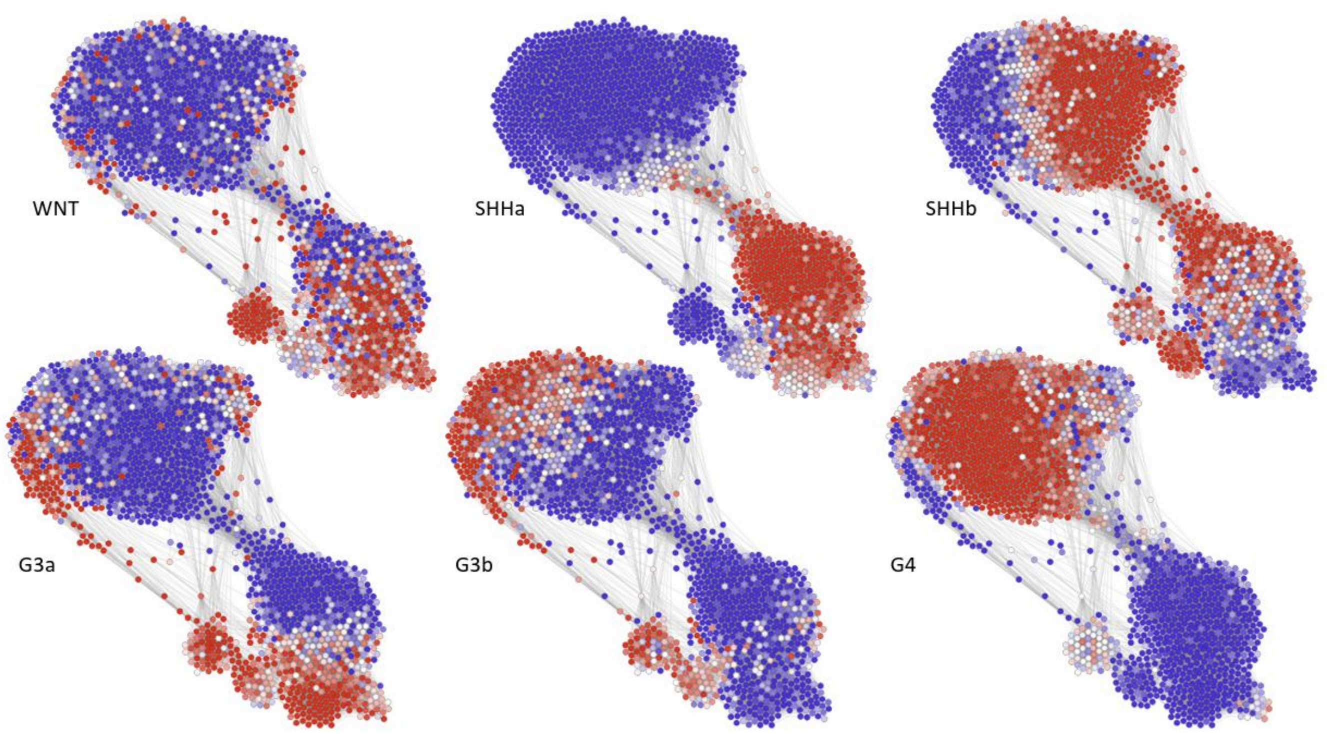

Mapping of WHO subtypes on scRNA-seq data (Aldinger et al, 366 genesets)

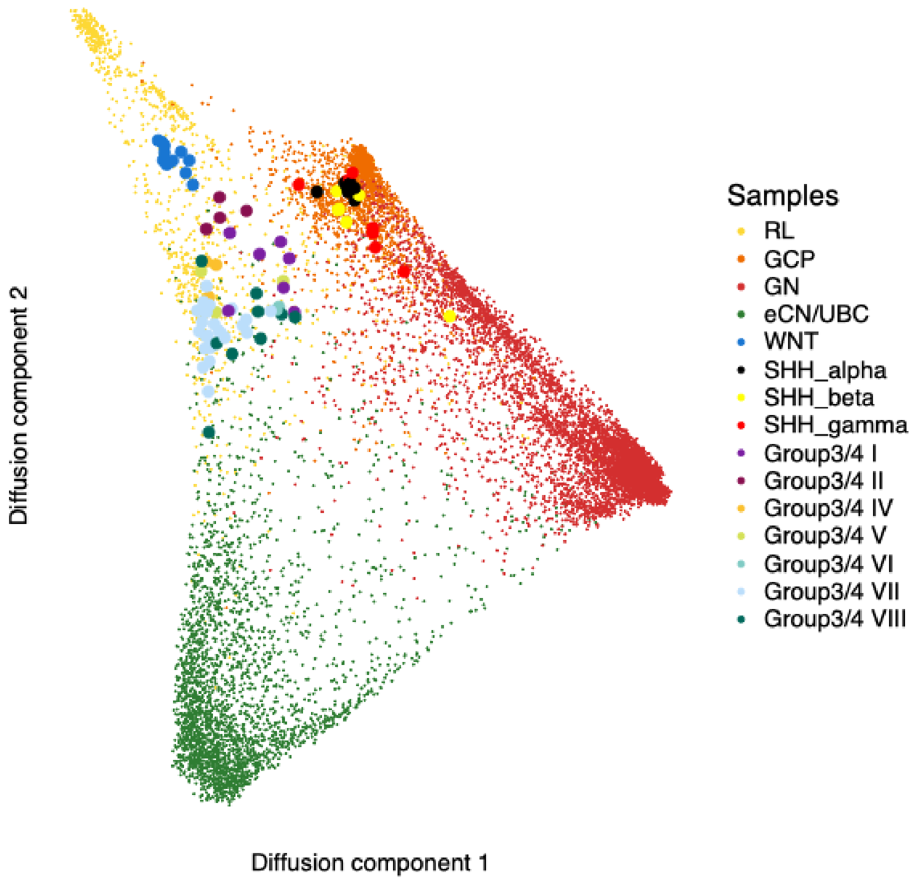

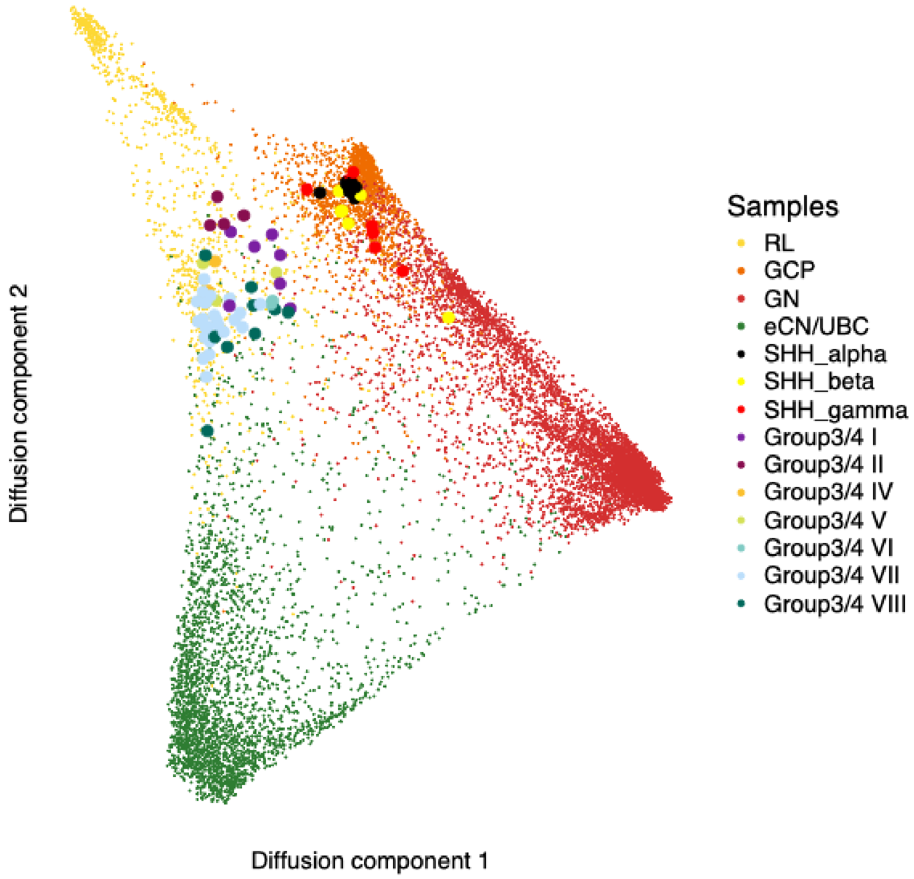

Proportion of cell lineage in medulloblastoma tissues
(scRNA-seq from Michael Taylor's group)

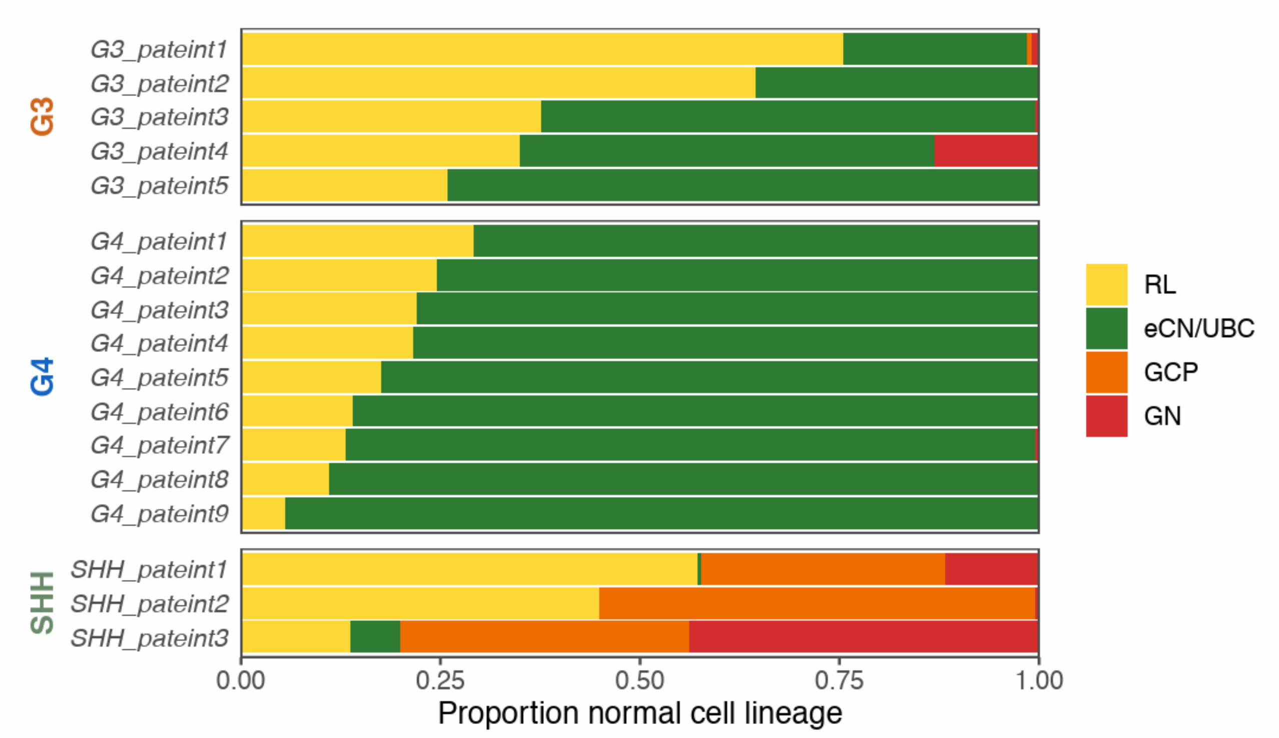

Proportion of cell lineage in each medulloblastoma tissue samples

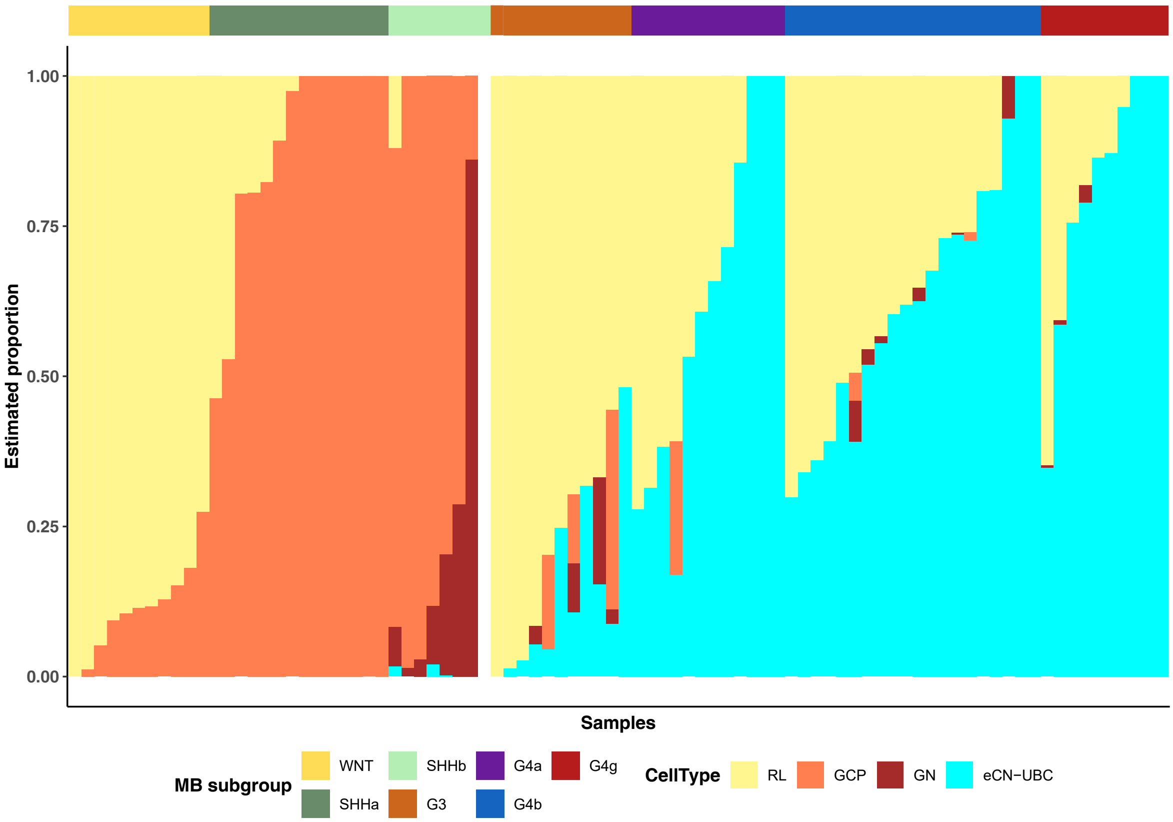

Park et al. Supplementary Fig. 12

Lineage-related gene expression on Diffusion map (scRNA-seq, Smith et al., 2022)

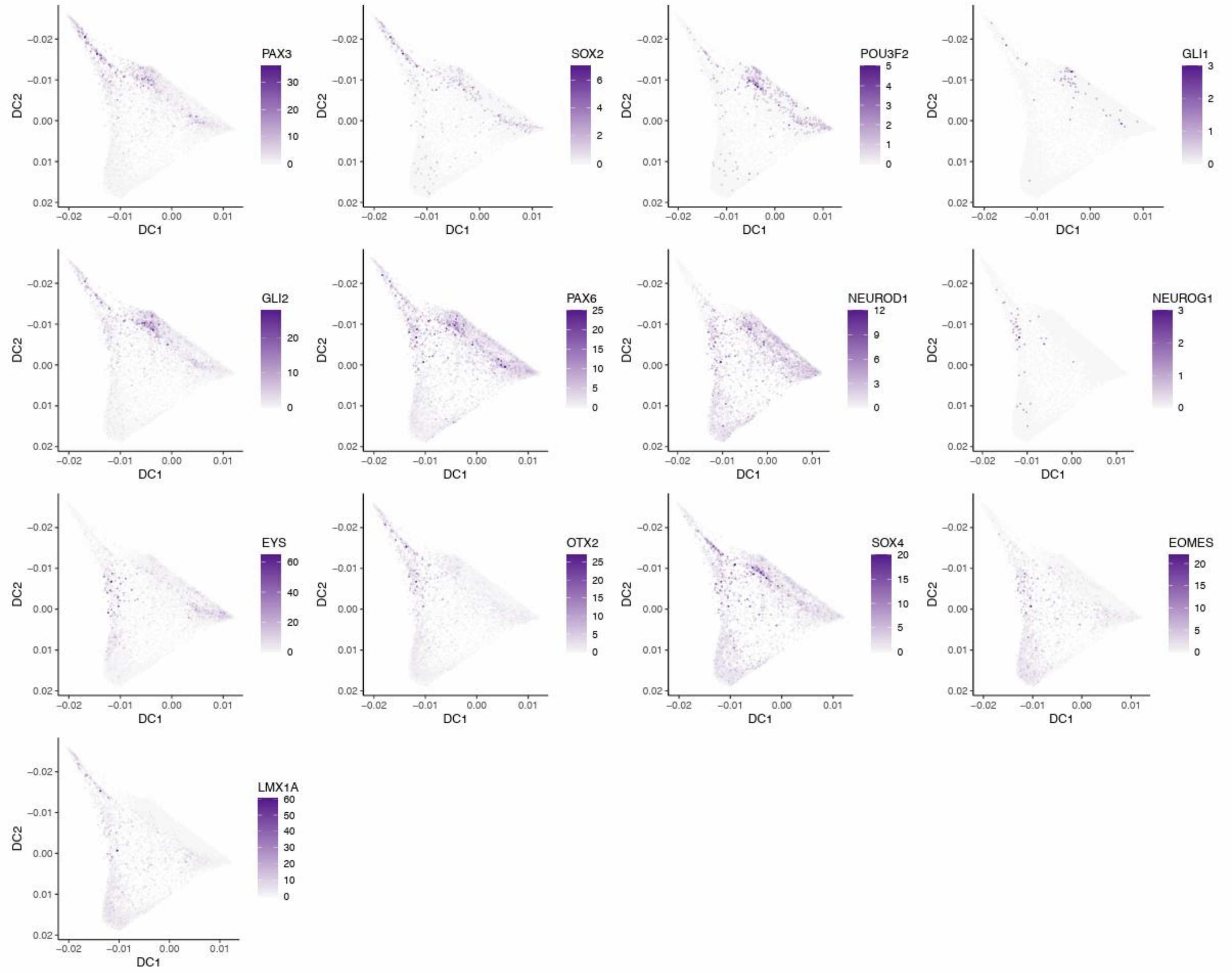

a

GSEA - NEUROD1 target gene expression

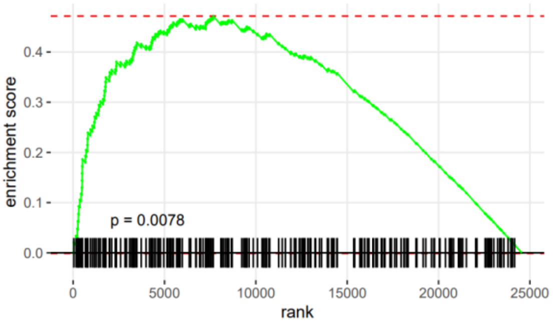

b

NEUROD1 RNA-neurotype 86 protein correlation (R)

p< 2.2 X 10^{-16} by t-test

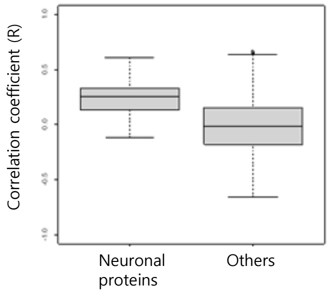

c

AMPH – NEUROD1 RNA
R= 0.5669745
p= 1.262 X 10^{-8}

SCNA – NEUROD1 RNA
R= 0.3653281
p= 0.0005428

SYN1 – NEUROD1 RNA
R= 0.3676445
p= 0.0004971

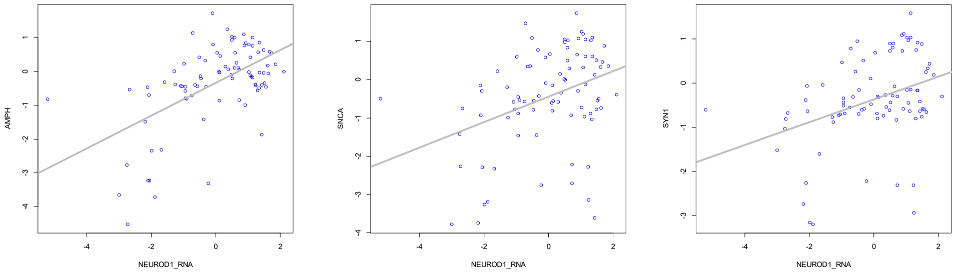

d

PFS in non-WNT patients
NEUROD1 RNA

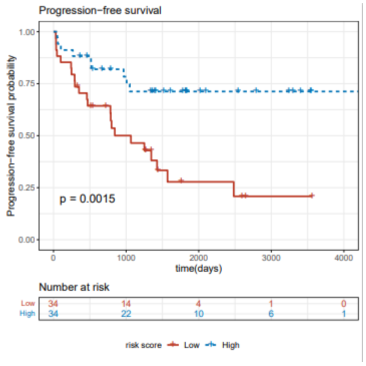

e

PFS in non-WNT patients
NEUROD1 target RNAs
(KCNA1, SCN2B, SLC1A2)

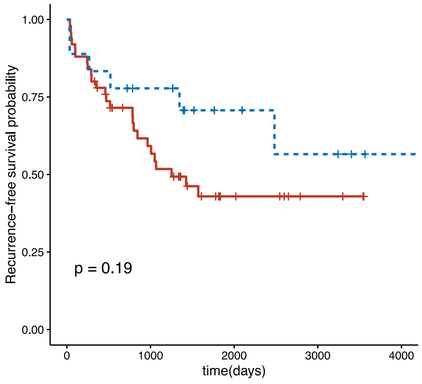

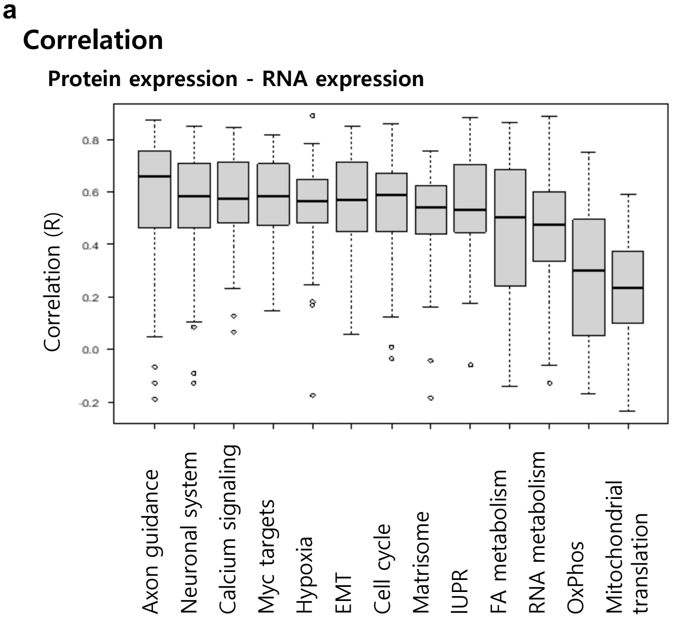
a
Correlation
Protein expression - RNA expression
Correlation (R)
Axon guidance
Neuronal system
Calcium signaling
Myc targets
Hypoxia
EMT
Cell cycle
Matrisome
UPR
FA metabolism
RNA metabolism
OxPhos
Mitochondrial translation

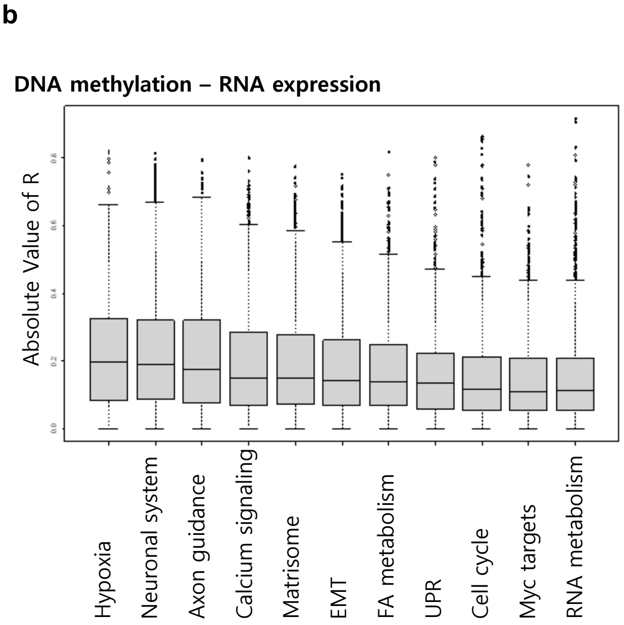
b
DNA methylation – RNA expression
Absolute Value of R
Hypoxia
Neuronal system
Axon guidance
Calcium signaling
Matrisome
EMT
FA metabolism
UPR
Cell cycle
Myc targets
RNA metabolism

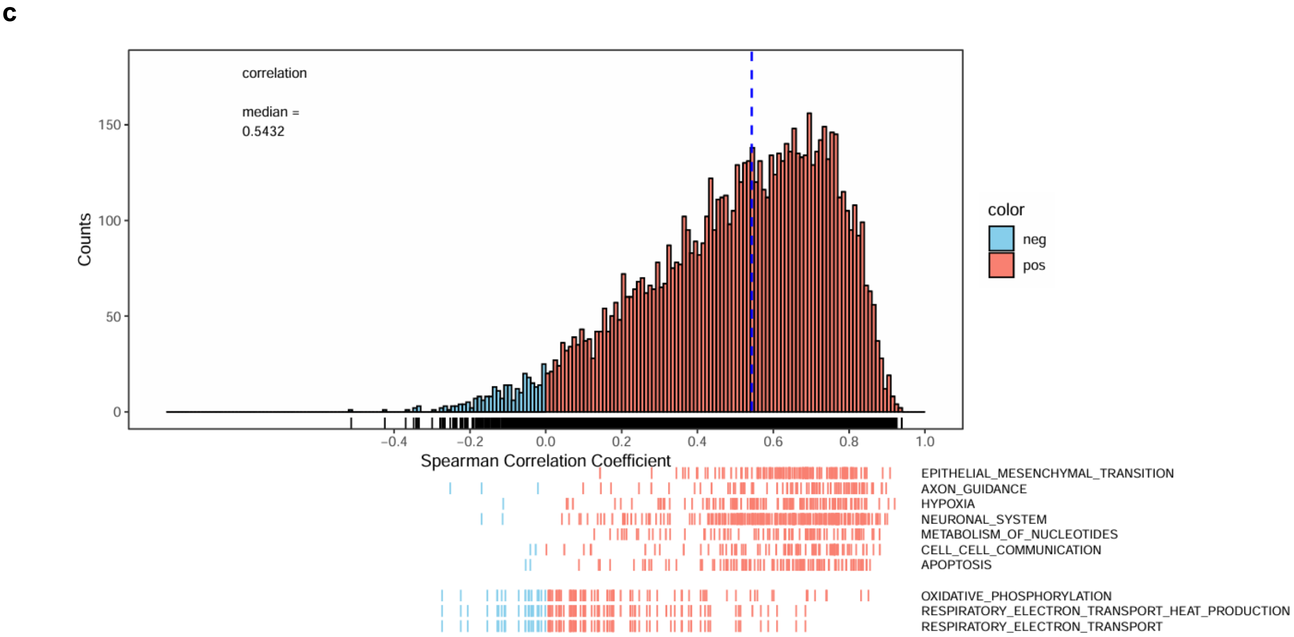
c
correlation
median = 0.5432
Counts
Spearman Correlation Coefficient
color
neg
pos
EPITHELIAL_MESENCHYMAL_TRANSITION
AXON_GUIDANCE
HYPOXIA
NEURONAL_SYSTEM
METABOLISM_OF_NUCLEOTIDES
CELL_CELL_COMMUNICATION
APOPTOSIS
OXIDATIVE_PHOSPHORYLATION
RESPIRATORY_ELECTRON_TRANSPORT_HEAT_PRODUCTION
RESPIRATORY_ELECTRON_TRANSPORT

Subtype-specific protein expression

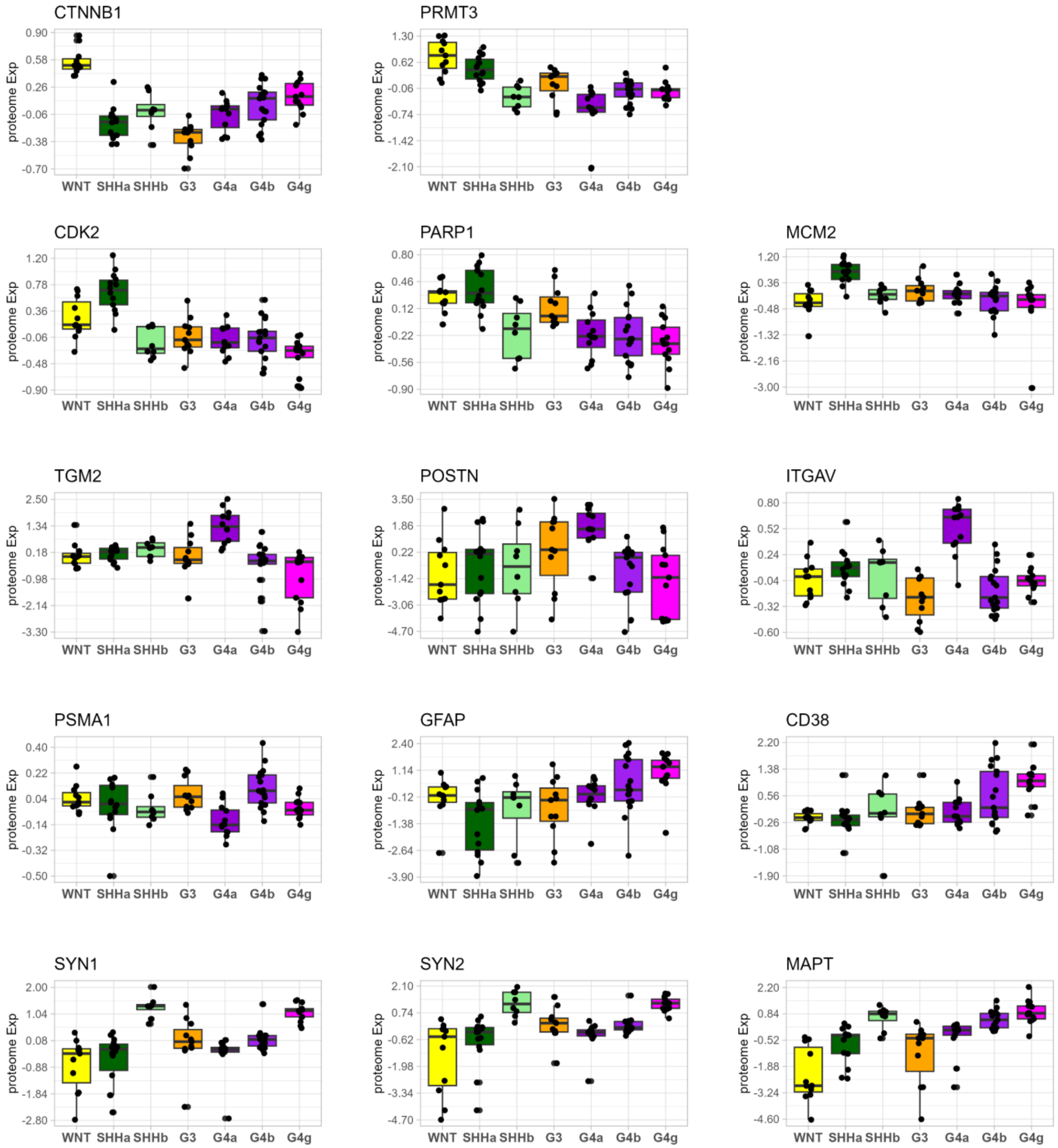

Tissue microarray - IHC (SHHa)

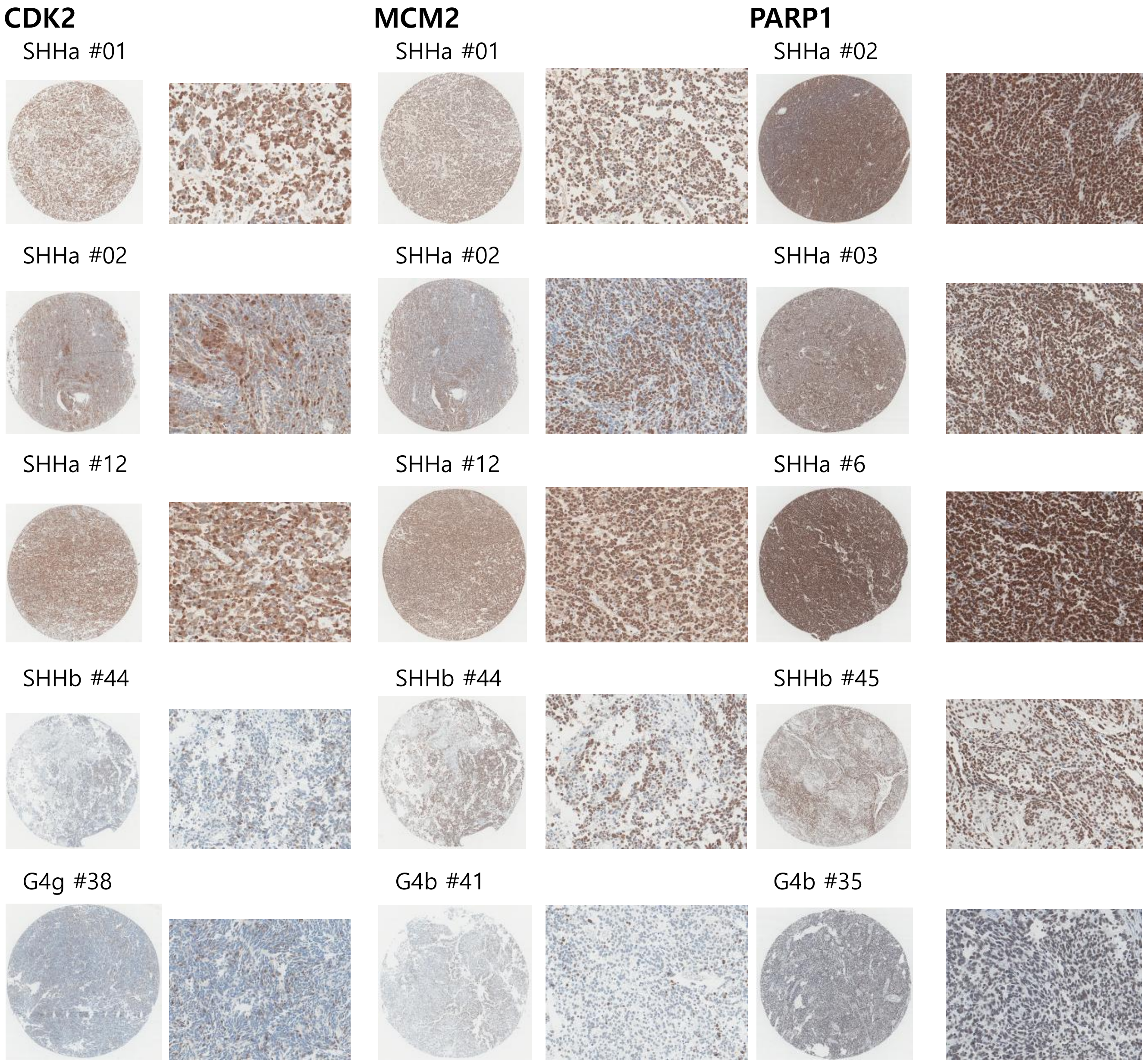

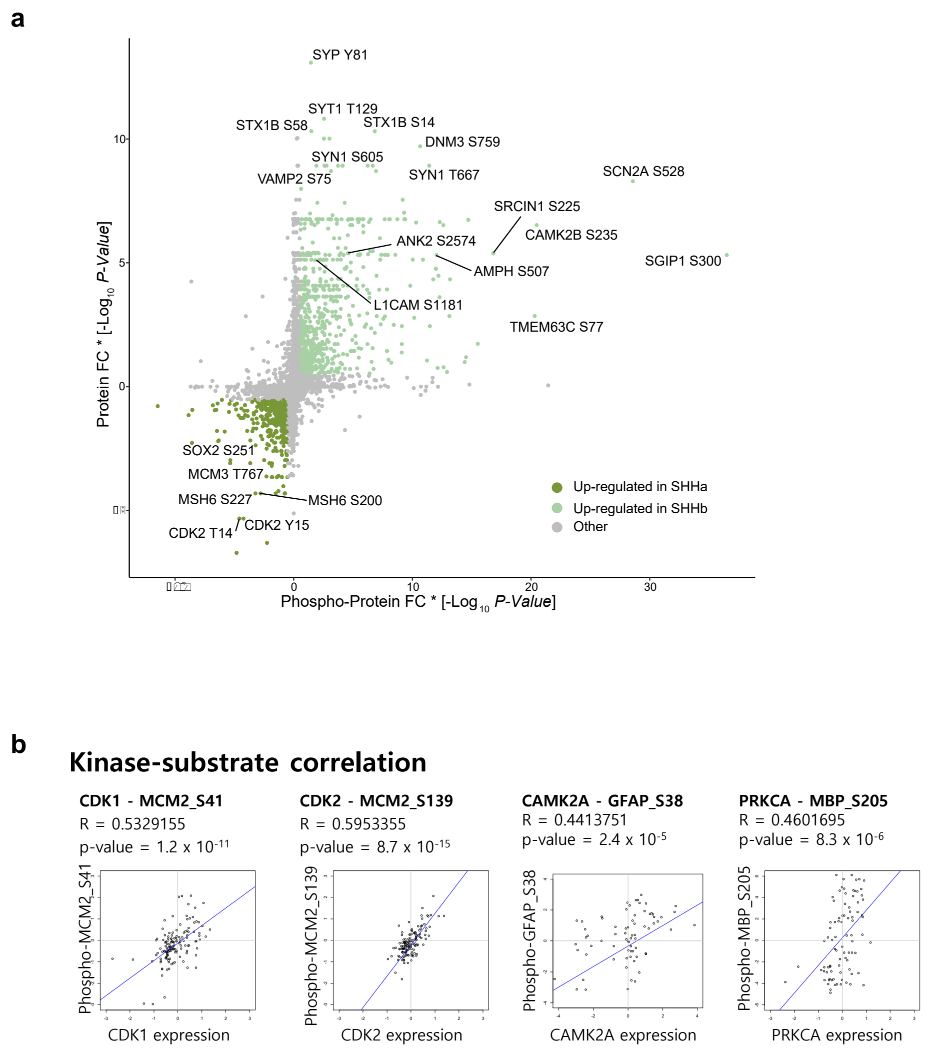
a
SYP Y81
SYT1 T129
STX1B S58
STX1B S14
DNM3 S759
SYN1 S605
SYN1 T667
SCN2A S528
VAMP2 S75
SRCIN1 S225
ANK2 S2574
CAMK2B S235
SGIP1 S300
AMPH S507
L1CAM S1181
TMEM63C S77
SOX2 S251
MCM3 T767
MSH6 S227
MSH6 S200
CDK2 T14
CDK2 Y15
Up-regulated in SHHa
Up-regulated in SHHb
Other
Protein FC * [-Log10 P-Value]
Phospho-Protein FC * [-Log10 P-Value]
b
Kinase-substrate correlation
CDK1 - MCM2_S41
R = 0.5329155
p-value = 1.2 x 10-11
CDK2 - MCM2_S139
R = 0.5953355
p-value = 8.7 x 10-15
CAMK2A - GFAP_S38
R = 0.4413751
p-value = 2.4 x 10-5
PRKCA - MBP_S205
R = 0.4601695
p-value = 8.3 x 10-6
Phospho-MCM2_S41
CDK1 expression
Phospho-MCM2_S139
CDK2 expression
Phospho-GFAP_S38
CAMK2A expression
Phospho-MBP_S205
PRKCA expression

Park et al. Supplementary Fig. 18

a PCA analysis with proteome

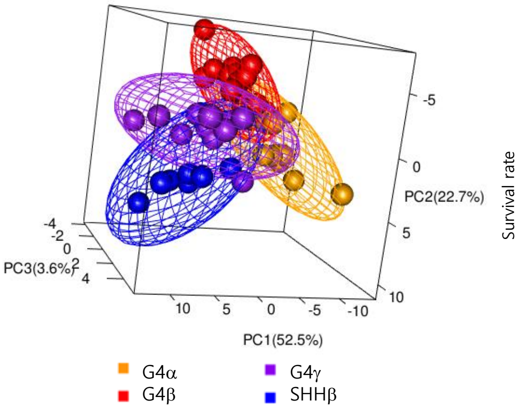

b Non-WNT medulloblastoma

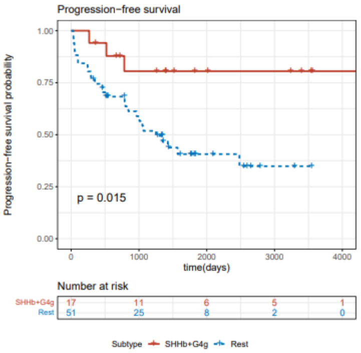

c Non-WNT medulloblastoma
Synaptic signaling proteins

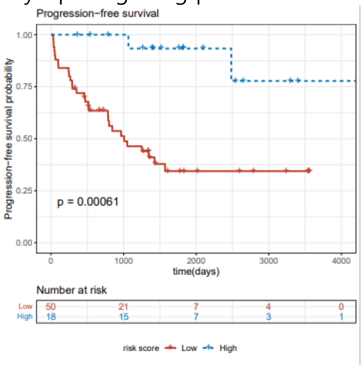

Kinase z-score (KSEA)

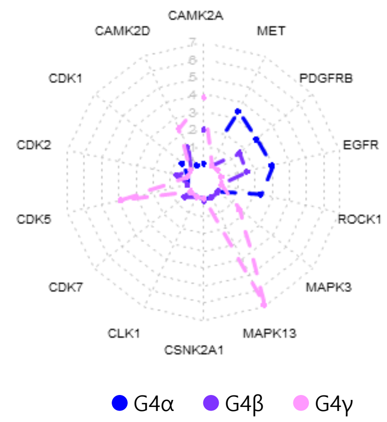

Kinase-substrate network

G4a

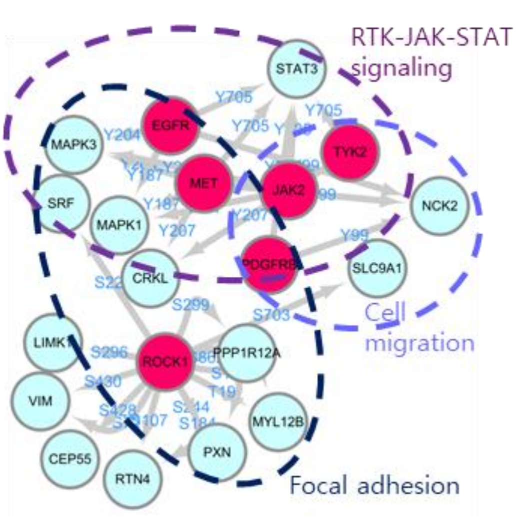

G4g

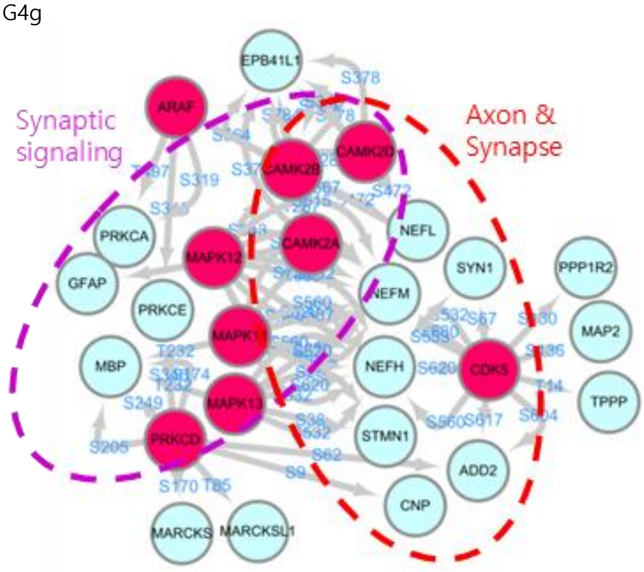

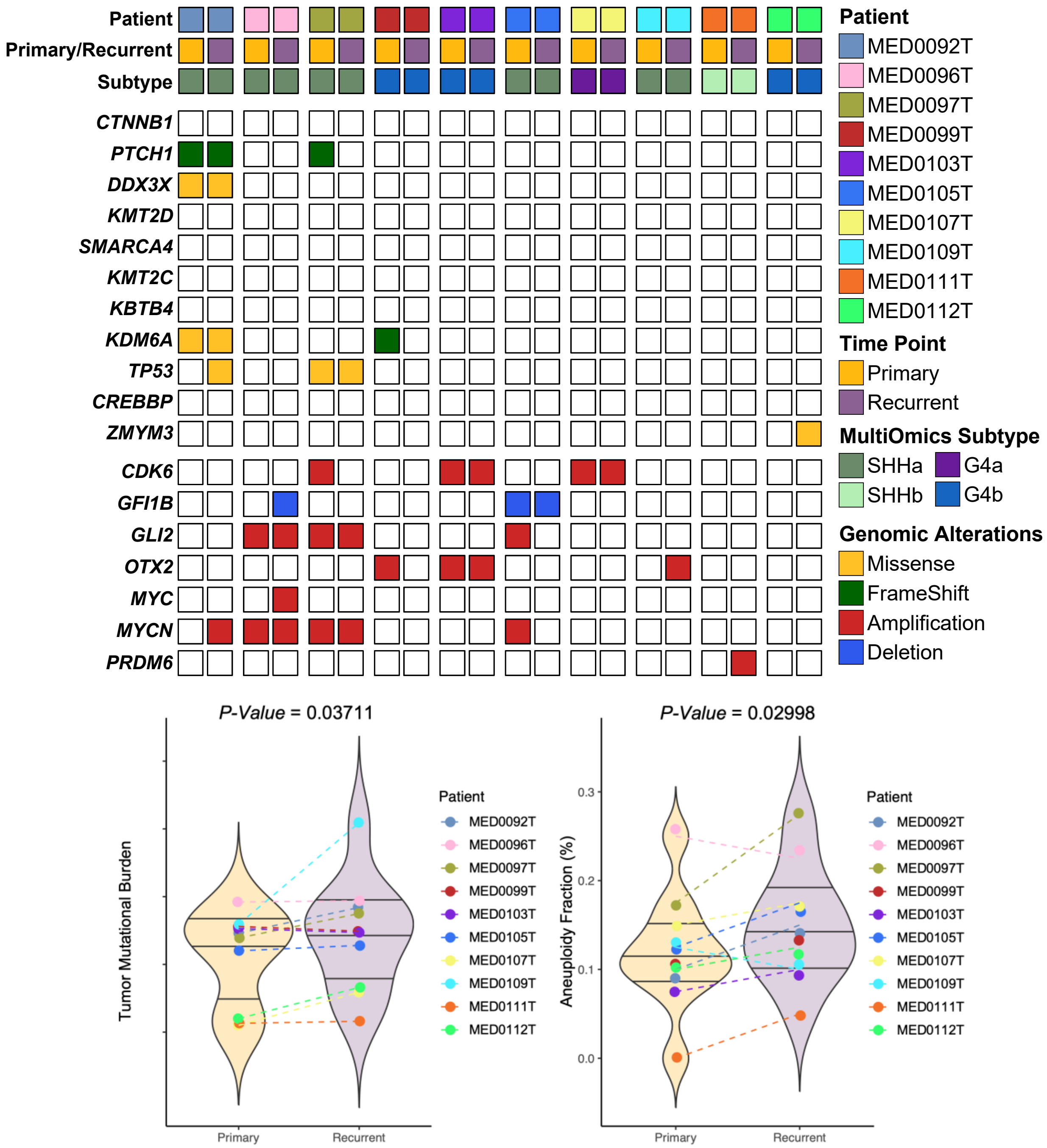
Patient
Primary/Recurrent
Subtype
CTNNB1
PTCH1
DDX3X
KMT2D
SMARCA4
KMT2C
KBTB4
KDM6A
TP53
CREBBP
ZMYM3
CDK6
GFI1B
GLI2
OTX2
MYC
MYCN
PRDM6
Patient
MED0092T
MED0096T
MED0097T
MED0099T
MED0103T
MED0105T
MED0107T
MED0109T
MED0111T
MED0112T
Time Point
Primary
Recurrent
MultiOmics Subtype
SHHa
G4a
SHHb
G4b
Genomic Alterations
Missense
FrameShift
Amplification
Deletion
P-Value = 0.03711
Tumor Mutational Burden
Primary
Recurrent
P-Value = 0.02998
Aneuploidy Fraction (%)
0.3
0.2
0.1
0.0

RNA-seq (Hierarchical clustering)

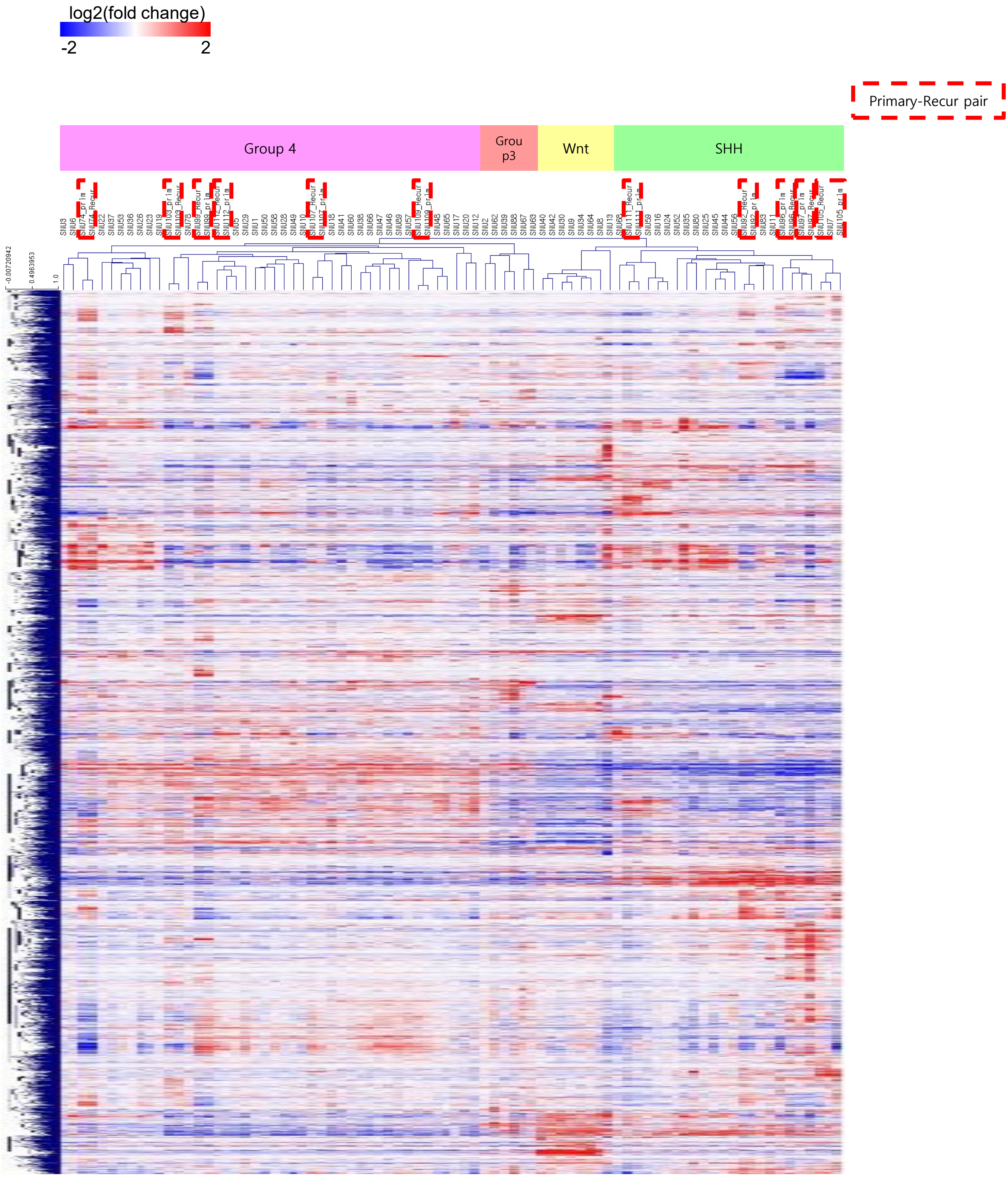

Cell type migration by recurrence on correlation network (RNA)

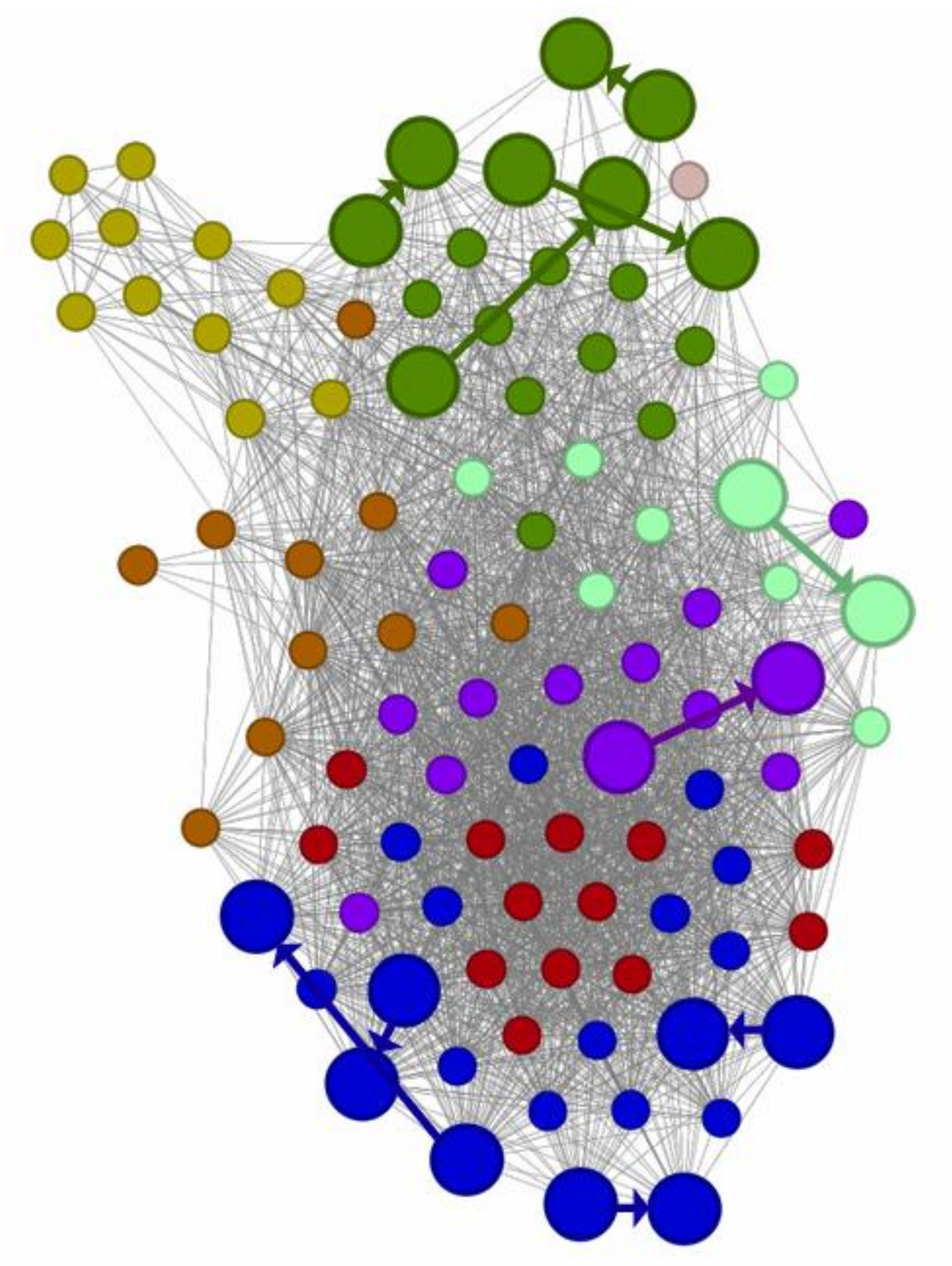

Park et al. Supplementary Fig. 23

NRP: non-recurrence primary
RPP: recurrence-potent primary

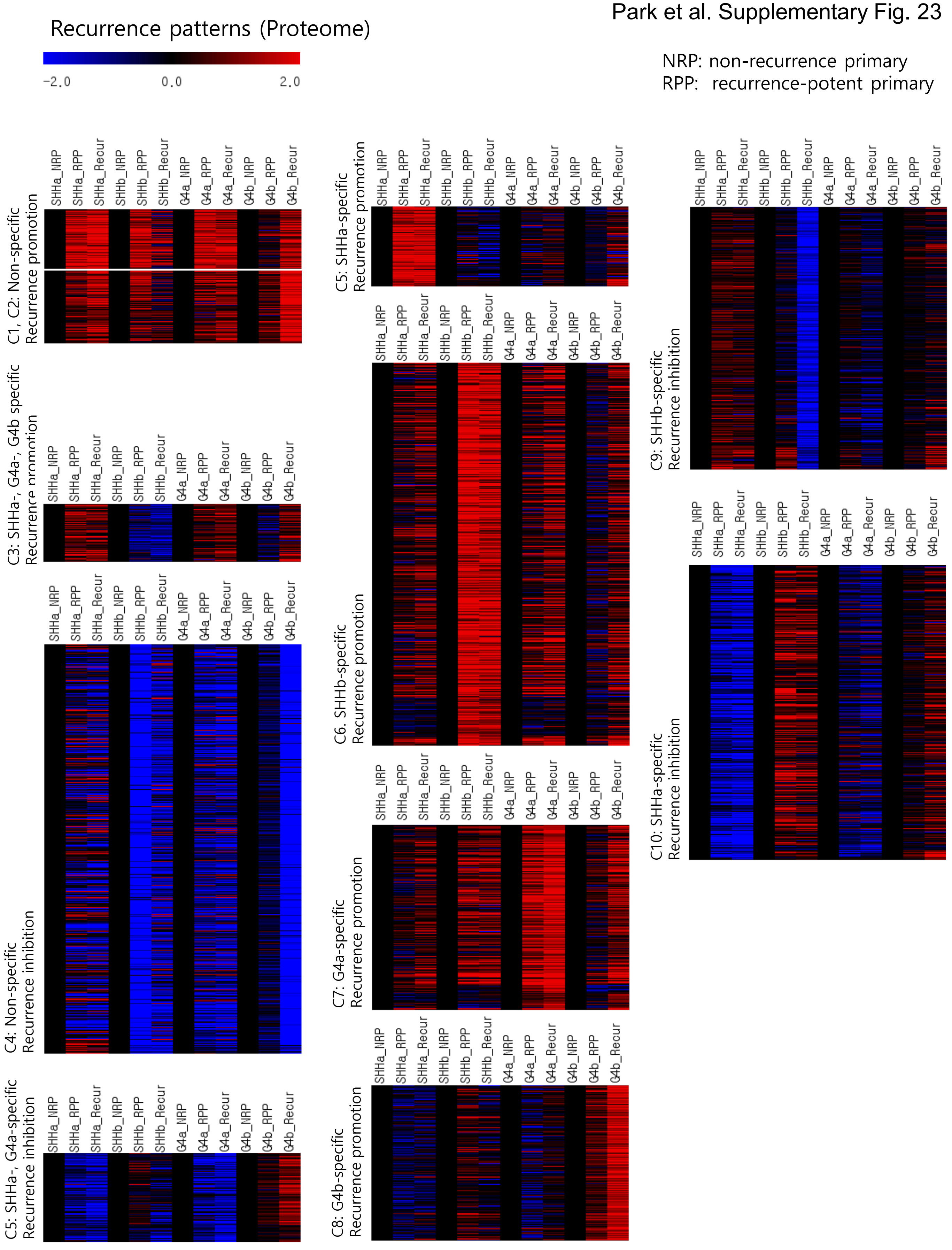

Park et al. Supplementary Fig. 24

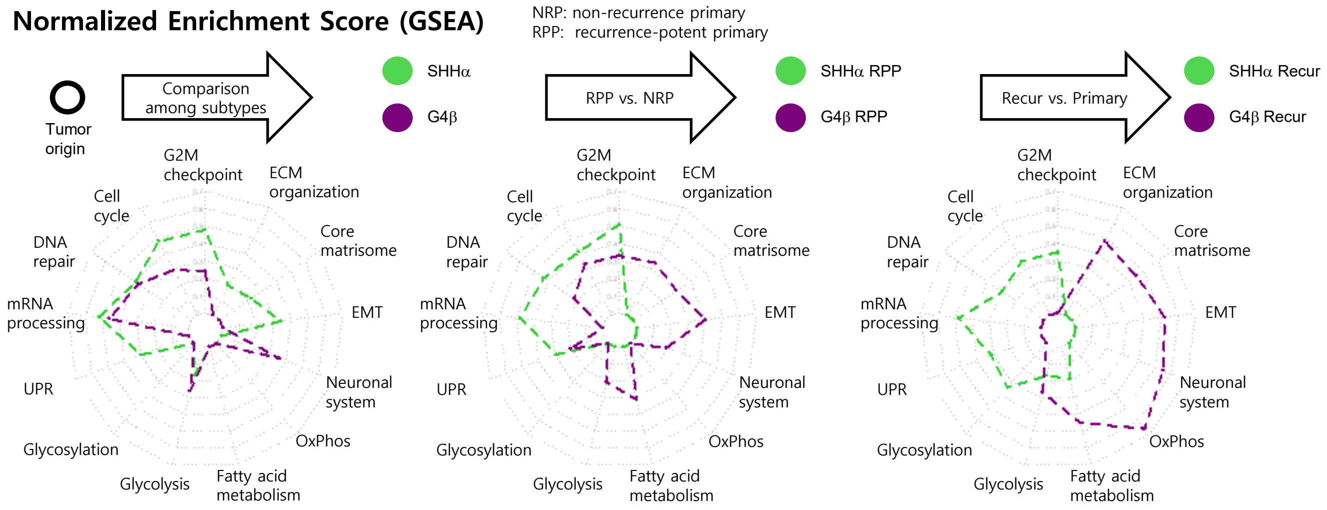

Representative protein expression by recurrence

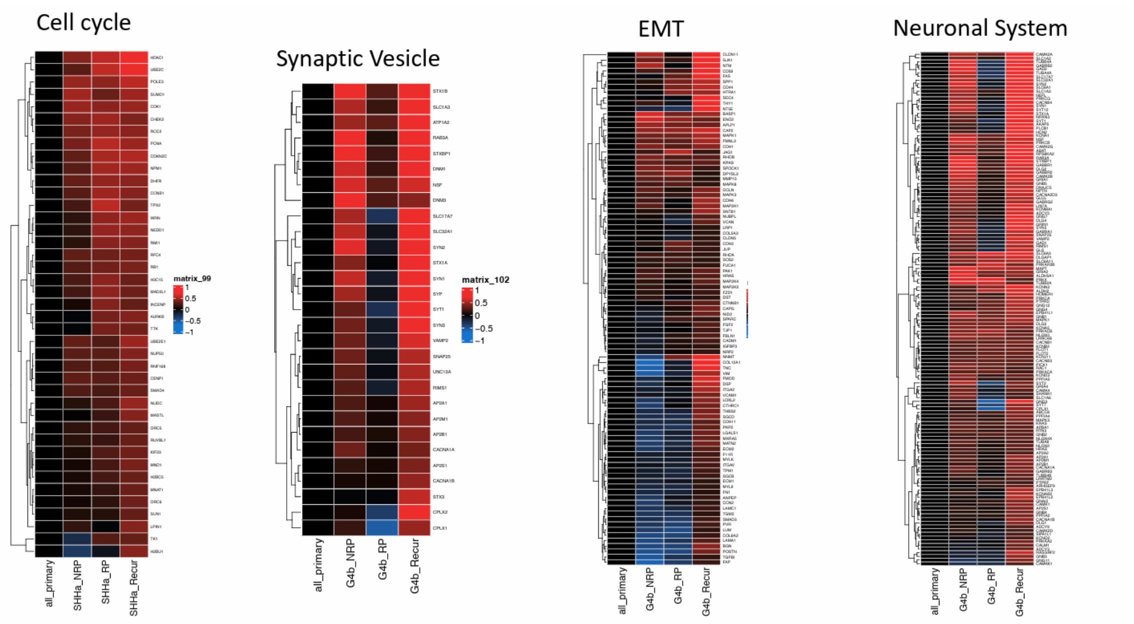

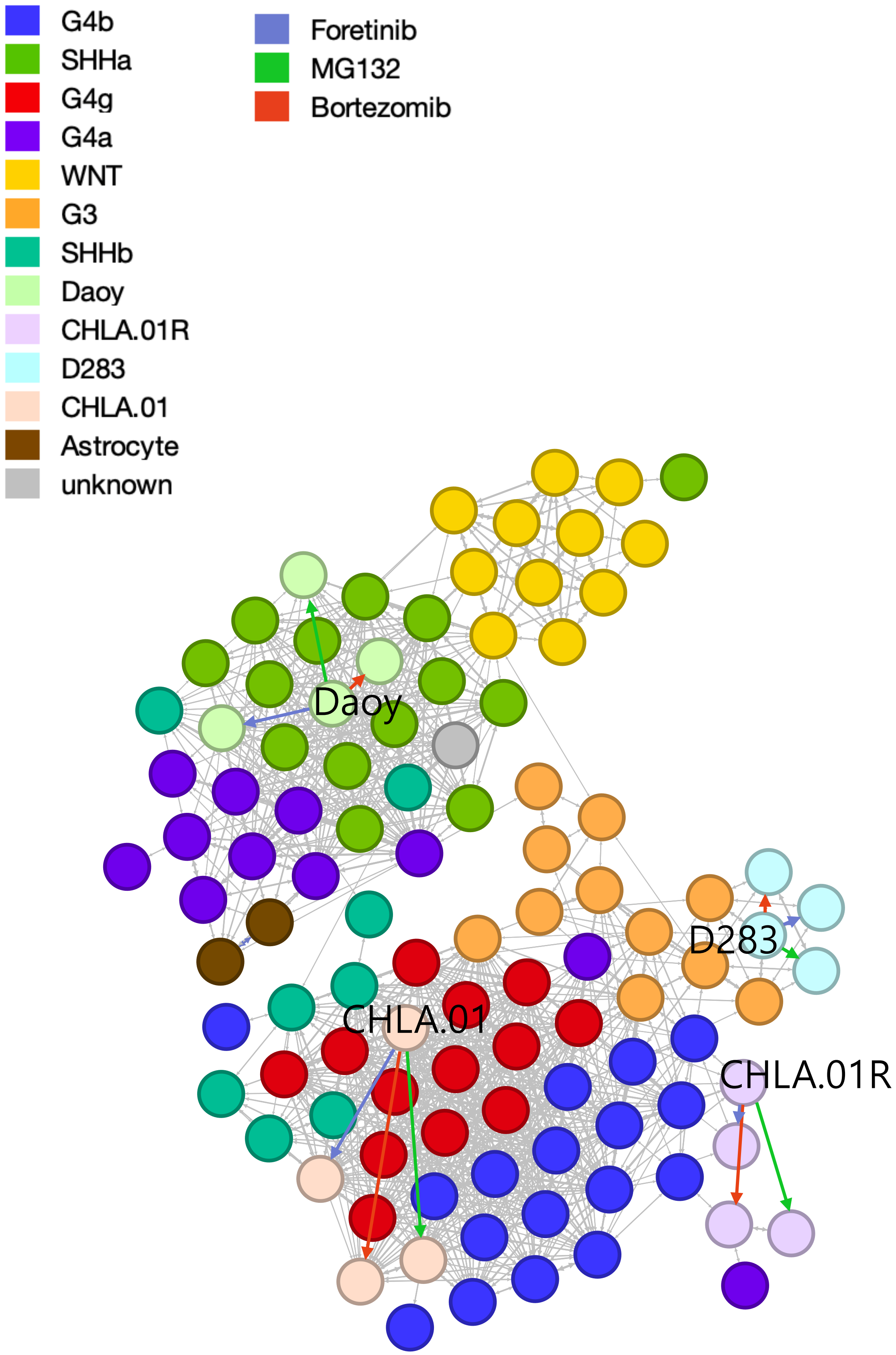
G4b
SHHa
G4g
G4a
WNT
G3
SHHb
Daoy
CHLA.01R
D283
CHLA.01
Astrocyte
unknown
Foretinib
MG132
Bortezomib
Daoy
D283
CHLA.01
CHLA.01R

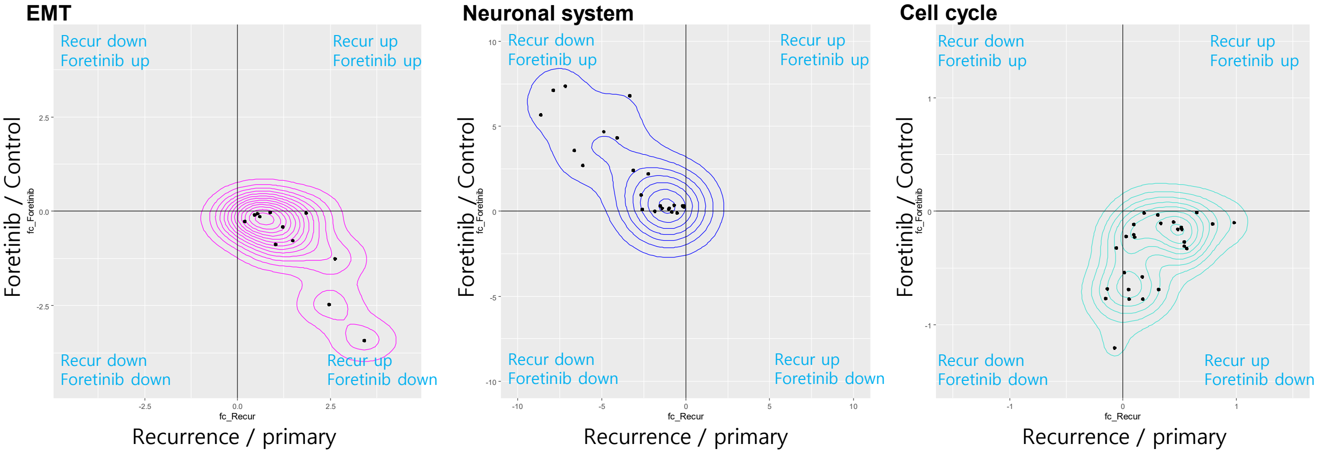
EMT
Recur down
Foretinib up
Recur up
Foretinib up
Recur down
Foretinib down
Recur up
Foretinib down
Foretinib / Control
fc_Foretinib
fc_Recur
Recurrence / primary
Neuronal system
Recur down
Foretinib up
Recur up
Foretinib up
Recur down
Foretinib down
Recur up
Foretinib down
Foretinib / Control
fc_Foretinib
fc_Recur
Recurrence / primary
Cell cycle
Recur down
Foretinib up
Recur up
Foretinib up
Recur down
Foretinib down
Recur up
Foretinib down
Foretinib / Control
fc_Foretinib
fc_Recur
Recurrence / primary

Park et al. Supplementary Fig. 28

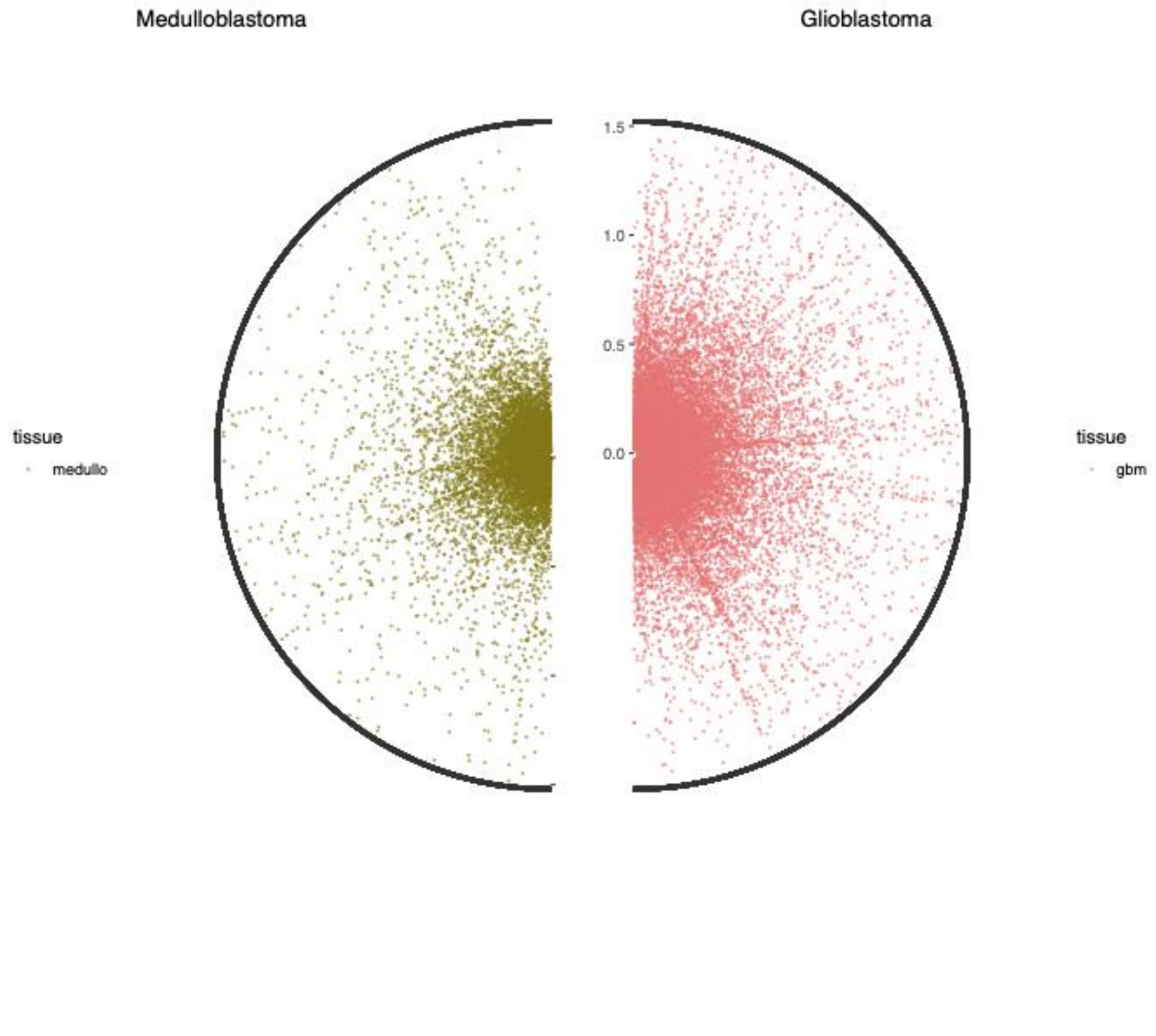

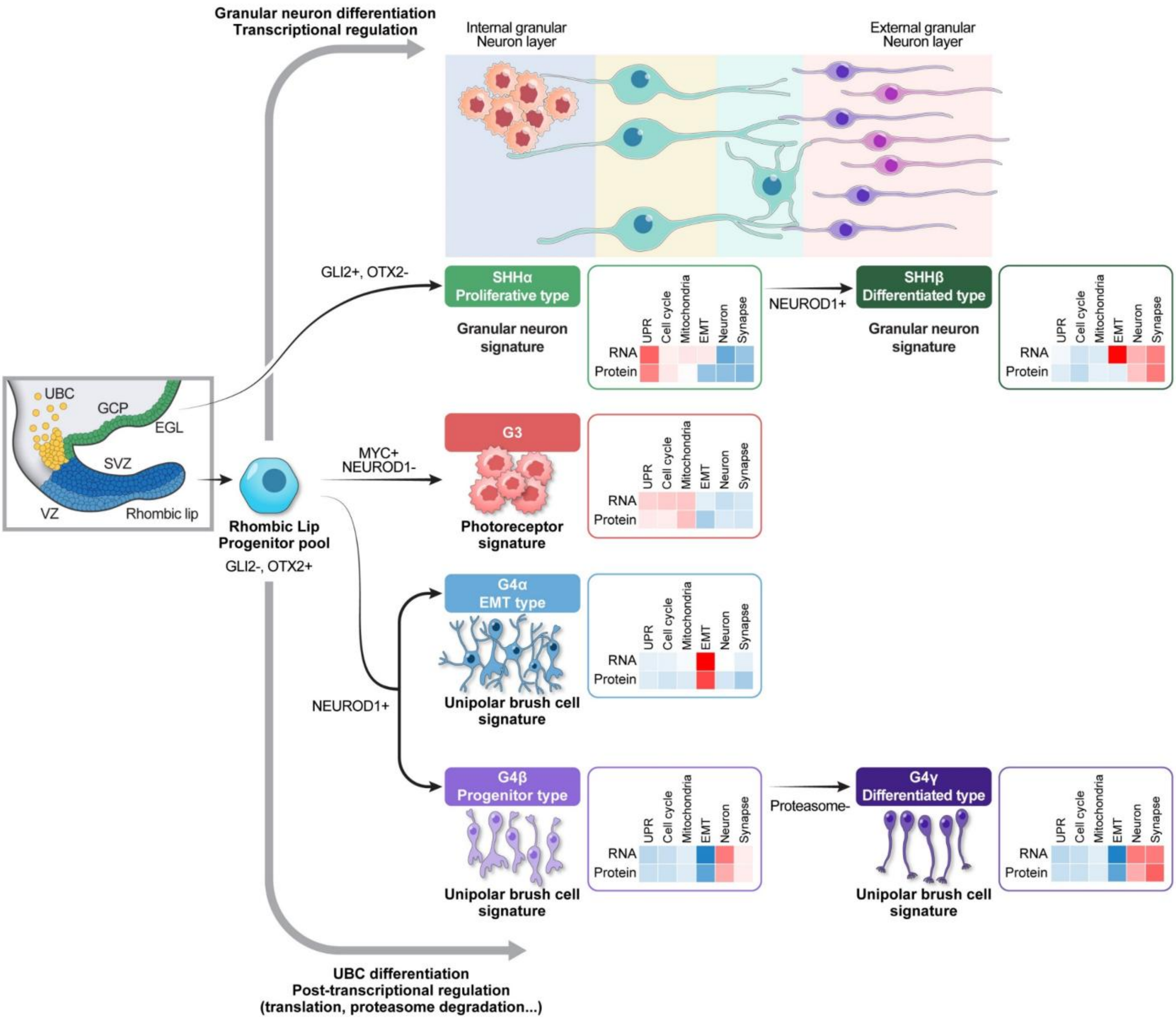

Granular neuron differentiation
Transcriptional regulation
Internal granular Neuron layer
External granular Neuron layer
GLI2+, OTX2-
SHHα Proliferative type
Granular neuron signature
UPR
Cell cycle
Mitochondria
EMT
Neuron
Synapse
RNA
Protein
NEUROD1+
SHHβ Differentiated type
Granular neuron signature
UBC
GCP
EGL
SVZ
VZ
Rhombic lip
Rhombic Lip Progenitor pool
GLI2-, OTX2+
MYC+
NEUROD1-
G3
Photoreceptor signature
G4α EMT type
Unipolar brush cell signature
NEUROD1+
G4β Progenitor type
Unipolar brush cell signature
Proteasome-
G4γ Differentiated type
Unipolar brush cell signature
UBC differentiation
Post-transcriptional regulation
(translation, proteasome degradation...)

Uncropped image (Fig. 5C)

Bortezomib

Synapsin-1

GAPDH

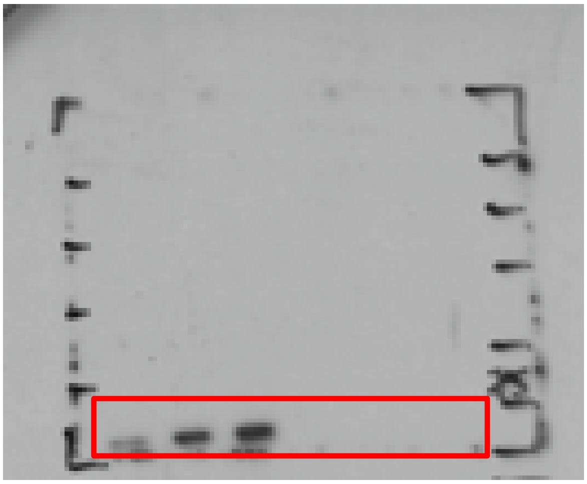

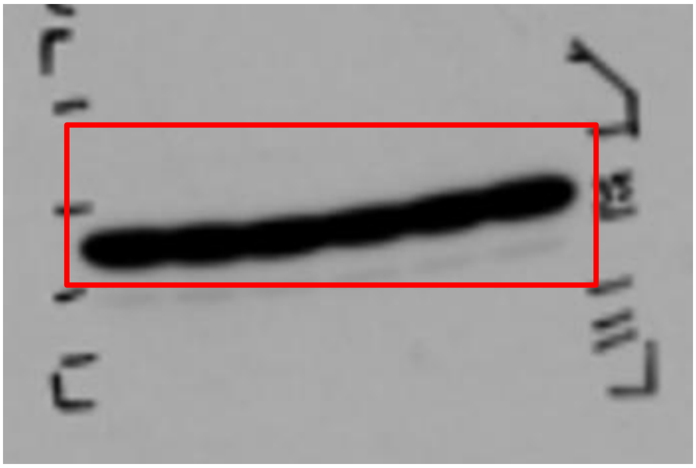

MG-132

Synapsin-1

GAPDH

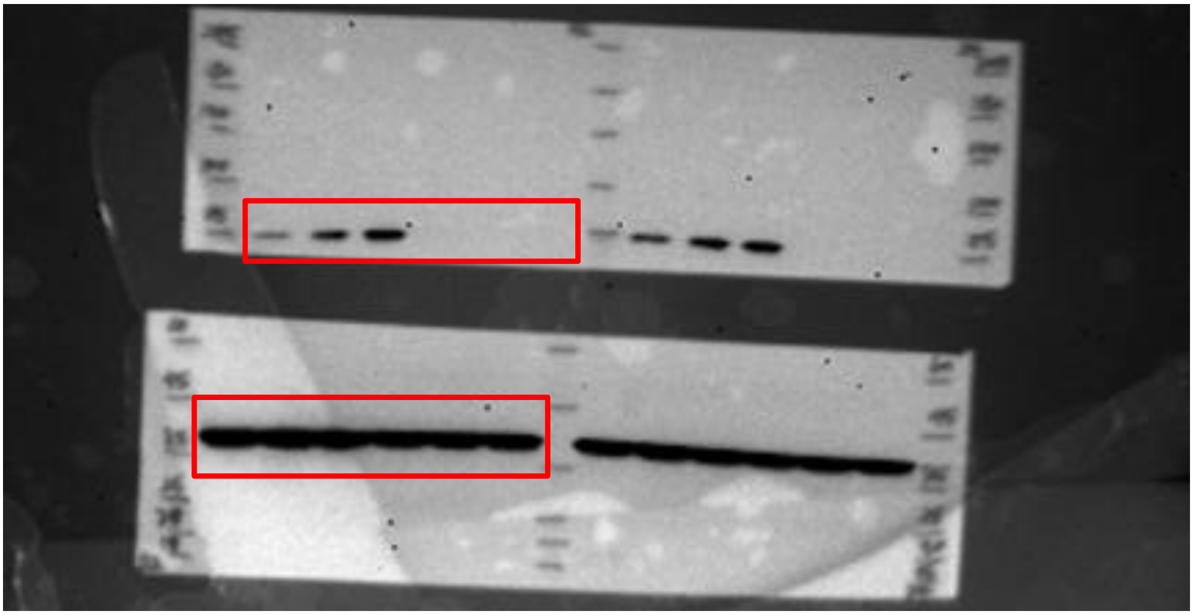

Supplement: Supplementary file 1 — Supplementary Information [file 12276_2026_1732_MOESM1_ESM.pdf]
